# Supermeres are functional extracellular nanoparticles replete with disease biomarkers and therapeutic targets

**DOI:** 10.1038/s41556-021-00805-8

**Published:** 2021-12-09

**Authors:** Qin Zhang, Dennis K. Jeppesen, James N. Higginbotham, Ramona Graves-Deal, Vincent Q. Trinh, Marisol A. Ramirez, Yoojin Sohn, Abigail C. Neininger, Nilay Taneja, Eliot T. McKinley, Hiroaki Niitsu, Zheng Cao, Rachel Evans, Sarah E. Glass, Kevin C. Ray, William H. Fissell, Salisha Hill, Kristie Lindsey Rose, Won Jae Huh, Mary Kay Washington, Gregory Daniel Ayers, Dylan T. Burnette, Shivani Sharma, Leonard H. Rome, Jeffrey L. Franklin, Youngmin A. Lee, Qi Liu, Robert J. Coffey

**Affiliations:** 1 Department of Medicine, Vanderbilt University Medical Center, Nashville, TN USA; 2 Section of Surgical Sciences, Vanderbilt University School of Medicine, Nashville, TN USA; 3 Department of Biostatistics, Vanderbilt University Medical Center, Nashville, TN USA; 4 Department of Cell and Developmental Biology, Vanderbilt University School of Medicine, Nashville, TN USA; 5 Division of Nephrology and Hypertension, Vanderbilt University Medical Center, Nashville, TN USA; 6 Proteomics Laboratory, Mass Spectrometry Research Center, Vanderbilt University School of Medicine, Nashville, TN USA; 7 Department of Biochemistry, Vanderbilt University School of Medicine, Nashville, TN USA; 8 Department of Pathology, Yale School of Medicine, New Haven, CT USA; 9 Department of Pathology, Microbiology and Immunology, Vanderbilt University Medical Center, Nashville, TN USA; 10 Department of Pathology and Laboratory Medicine, University of California at Los Angeles, Los Angeles, CA USA; 11 California NanoSystems Institute, University of California at Los Angeles, Los Angeles, CA USA; 12 Department of Biological Chemistry, David Geffen School of Medicine, University of California Los Angeles, Los Angeles, CA USA

**Keywords:** Mass spectrometry, Cellular imaging, Cell biology, Cancer models

## Abstract

Extracellular vesicles and exomere nanoparticles are under intense investigation as sources of clinically relevant cargo. Here we report the discovery of a distinct extracellular nanoparticle, termed supermere. Supermeres are morphologically distinct from exomeres and display a markedly greater uptake in vivo compared with small extracellular vesicles and exomeres. The protein and RNA composition of supermeres differs from small extracellular vesicles and exomeres. Supermeres are highly enriched with cargo involved in multiple cancers (glycolytic enzymes, TGFBI, miR-1246, MET, GPC1 and AGO2), Alzheimer’s disease (APP) and cardiovascular disease (ACE2, ACE and PCSK9). The majority of extracellular RNA is associated with supermeres rather than small extracellular vesicles and exomeres. Cancer-derived supermeres increase lactate secretion, transfer cetuximab resistance and decrease hepatic lipids and glycogen in vivo. This study identifies a distinct functional nanoparticle replete with potential circulating biomarkers and therapeutic targets for a host of human diseases.

## Abstract (web summary)

Zhang et al. identify and characterize supermeres as extracellular nanoparticles that exhibit unique biological and functional properties with potential prognostic and therapeutic value across distinct diseases.

## Main

There is an increasing appreciation for the heterogeneous nature of secreted extracellular vesicles (EVs) and non-vesicular (NV) nanoparticles^1–3^. Exosomes are 40–150 nm endosome-derived, lipid bilayer-enclosed small EVs (sEVs)^1,4,5^. A type of small (<50 nm) non-membranous extracellular nanoparticle, termed exomere, was recently identified^2^. Both exosomes and exomeres are released by most cells and tissues under both physiological and pathological conditions. Their production and content seem to be altered in a number of disease states, including neoplastic, cardiovascular, immunological and neurological disorders. However, intrinsic heterogeneity and variable methods of isolation pose major challenges to realizing their clinical potential.

This study was initially designed to provide a comprehensive proteomic and RNA analysis of clinically relevant cargo unique to exosomes and exomeres in a human colorectal cancer (CRC) cell line, DiFi, using an optimized strategy to purify sEVs^1^ and a simplified method to isolate exomeres^3^. We recently reported that high-speed ultracentrifugation of the sEV supernatant results in the isolation of amembranous nanoparticles identical in morphology and content to that reported in the original characterization of exomeres using asymmetric flow field-flow fractionation^2^. Early on in the study, we speculated that high-speed ultracentrifugation of the exomere supernatant might identify an additional population of nanoparticles and, indeed, we discovered a distinct nanoparticle that we have termed supermere (supernatant of exomeres).

Supermeres were morphologically and structurally distinct from exomeres as determined by fluid-phase atomic force microscopy (AFM). These nanoparticles displayed different cellular-uptake kinetics than sEVs and exomeres in vitro and exhibited a markedly greater uptake in vivo in all of the examined tissues compared with sEVs and exomeres. Many of the clinically relevant proteins (amyloid precursor protein (APP), cellular-mesenchymal-epithelial transition factor (MET), glypican 1 (GPC1), argonaute-2 (AGO2), TGFβ-induced (TGFBI), numerous glycolytic enzymes) and extracellular RNA (exRNA; miR-1246)) previously reported to be in exosomes, were highly enriched in supermeres. Notably, the majority of the exRNA was associated with supermeres rather than sEVs and exomeres. We identified three functional properties of cancer-derived supermeres: increased lactate secretion in recipient cells (a hallmark of the Warburg effect), transfer of cetuximab resistance to cetuximab-sensitive cells and altered liver metabolism following systemic injection. Supermeres in the circulation were detectable by optimized flow cytometry, opening up their investigation in liquid biopsies as sources of biomarkers and therapeutic targets.

We performed mass spectrometry of DiFi sEVs, exomeres and supermeres. The most abundant protein in highly purified sEVs was DPEP1, a glycophosphatidylinositol (GPI)-linked dipeptidase that has been reported to be upregulated in colorectal adenomas and CRC^6^. Diffuse localization of DPEP1 in a clinically well-annotated CRC tissue microarray (TMA) portended a worse outcome, and DPEP1 was increased in sEVs isolated from the plasma of patients with CRC compared with control individuals.

Together, this work identifies a functional extracellular nanoparticle that is morphologically and molecularly distinct from exosomes and is replete with potential biomarkers and targets for drug discovery. Moreover, we demonstrate the ability to isolate and inventory the contents of distinct populations of sEVs and nanoparticles so as to assign cargo to their correct carriers. These findings have important implications for cancer, Alzheimer’s disease, heart disease and coronavirus disease 2019 (COVID-19) infection.

## Results

### Supermeres display distinct uptake in vitro and in vivo

**Fig. 1:**
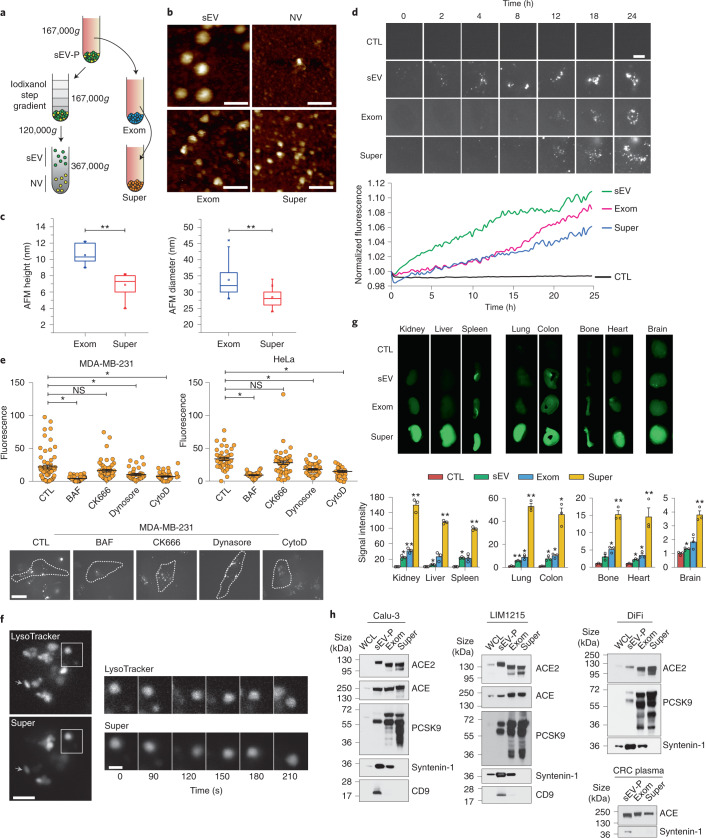
Supermeres display distinct uptake in vitro and in vivo. **a**, Simplified schematic illustration of the supermere isolation procedure. **b**, Representative fluid-phase AFM topographic images of sEVs (top left), NV fractions (top right), exomeres (bottom left) and supermeres (bottom right) derived from DiFi cells. Scale bars, 100 nm. **c**, Exomere and supermere heights (left) and diameters (right) measured by AFM (mean ± s.e.m). Height, *n* = 10; and diameter, *n* = 134, where *n* is the number of nanoparticles. For the boxplots, the centre lines mark the median, the box limits indicate the 25th and 75th percentiles, and the whiskers extend 1.5× the interquartile range from the 25th and 75th percentiles. **d**, Imaging of vesicle and particle uptake (top). MDA-MB-231 cells were incubated with PBS (CTL, control), or Alexa Fluor-647-labelled sEVs, exomeres or supermeres, and imaged every 15 min for 24 h using an instant SIM (iSIM) imaging system. Each field of view was averaged and normalized to the starting value (bottom); *n* = 3 fields of view for each 15 min time point. Data are representative of two independent experiments. Scale bar, 10 µm. **e**, Inhibition of cellular supermere uptake. Cells were pre-incubated with uptake inhibitors for 30 min before the addition of labelled supermeres. After a 24 h incubation, images were acquired using an iSIM imaging system (bottom). Data are the mean ± s.e.m. of *n* = 30 (MDA-MB-231) and 27 (HeLa) cells (top). Images are representative of three independent experiments. The dashed white lines represent the region of interest (ROI). Scale bar, 20 µm. BAF, bafilomycin A1; and CytoD, cytochalasin D. **f**, Supermere co-localization with endo/lysosomal compartments following uptake. MDA-MB-231 cells were incubated with labelled supermeres and stained with LysoTracker. Images were acquired using an iSIM imaging system. Data are representative of two independent experiments. A time montage of the regions in the white boxes on the left is shown (right). Scale bar, 5 µm (left) and 2 µm (right). **g**, Whole-organ imaging (top). Male C57BL/6 mice were intraperitoneally injected with labelled sEVs, exomeres or supermeres derived from DiFi cells. Their organs were harvested and analysed after 24 h. Data are the mean ± s.e.m. of *n* = 3 animals (bottom). **h**, Immunoblots of select proteins in the sEV-P, exomeres and supermeres derived from cell lines and a plasma sample from a patient with CRC. WCL, whole-cell lysate; exom, exomere; and super, supermere. Statistical significance was determined using a two-tailed Student’s *t*-test (**c**,**g**) or one-way analysis of variance (ANOVA) with Holm–Bonferroni correction (**e**); NS, not significant; *P < 0.01 and **P *<* 0.001. Source data

**Extended Data Fig. 1:**
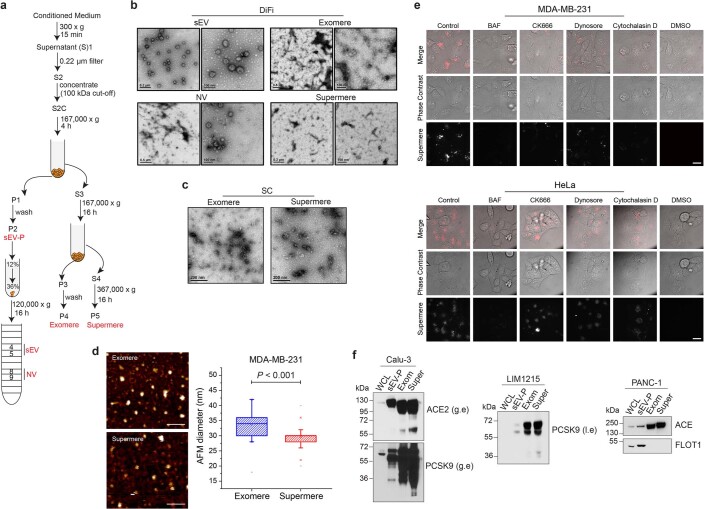
Supermeres are extracellular particles with distinct uptake *in vitro*. **a**, Schematic of the isolation procedure for the sEV-P, sEVs, NV, exomeres and supermeres. **b**, Negative stain transmission electron microscopy of DiFi-derived sEVs, NV, exomeres and supermeres. **c**, Negative stain transmission electron microscopy of SC-derived exomeres and supermeres. **d**, Representative fluid-phase AFM topographic images of exomeres and supermeres derived from MDA-MB-231 cells. Scale bar, 100 nm (left). Box plots of exomere and supermere diameters measured by AFM. *n* = 108 particles, **P < 0.001 (two-tailed *t*-test). For boxplots the centre lines mark the median; box limits indicate 25th and 75th percentiles; whiskers extend 1.5 times the interquartile range from 25th and 75th percentiles (right). **e**, Inhibition of cellular supermere uptake. MDA-MB-231 or HeLa cells were pre-incubated with indicated uptake inhibitors for 30 min before addition of Alexa Fluor-647-labelled supermeres. After 24 h incubation, bright-field and fluorescence images were acquired with iSIM. Scale bar, 20 µm. **f**, Immunoblot of selected proteins in Calu-3-, LIM1215-, and PANC-1-derived sEV-Ps, exomeres (exom) and supermeres (super). Thirty micrograms of protein from each fraction were analysed. WCL, whole-cell lysate. l.e, lower exposure; g.e, greater exposure. Source data

To determine whether other nanoparticle types remained after exomere depletion, we modified a sequential high-speed ultracentrifugation protocol (Fig. 1a). Crude sEV pellets (sEV-Ps) were prepared by ultracentrifugation and for some experiments, the sEV-P samples were further fractionated on high-resolution density gradients to separate highly pure vesicles (sEV) from NV components, as previously described^1^. Next, the exomeres were pelleted and the resulting supernatants were subjected to ultracentrifugation at 367,000*g* to obtain a pellet we termed supermere (Fig. 1a and Extended Data Fig. 1a). Fluid-phase AFM and transmission electron microscopy (TEM) imaging revealed that the morphological structure of supermeres was distinct from sEVs, NV nanoparticles and exomeres derived from two human CRC cell lines, DiFi and HCA-7-derived spiky colony (SC)^7^ (Fig. 1b and Extended Data Fig. 1b,c), and from the human breast cancer cell line MDA-MB-231 (Extended Data Fig. 1d). Under identical force and imaging conditions, supermeres exhibited smaller heights and diameters than other fractions (Fig. 1c and Extended Data Fig. 1d). Ellipsoid approximation of AFM-based volumes (essentially proportional to the mass) indicated that the volume of exomeres is about twice that of supermeres (approximately 5,894 nm^3^ versus 2,872 nm^3^, respectively).

To investigate uptake dynamics in vitro, we fluorescently labelled sEVs, exomeres and supermeres derived from DiFi cells and treated MDA-MB-231 cells for 24 h. Supermeres and exomeres displayed significantly slower cellular uptake compared with sEVs (Fig. 1d). To examine the potential mechanisms of supermere uptake, we pretreated MDA-MB-231 and HeLa cells with inhibitors that block different cellular-uptake pathways before adding fluorescently labelled supermeres. In both cell types, treatment with bafilomycin A caused the greatest inhibition of supermere accumulation, suggesting that macropinocytosis is a potential mechanism, although inhibitors targeting endocytosis also significantly reduced internalization (Fig. 1e and Extended Data Fig. 1e). Previous studies have shown that sEVs can enter endolysomal compartments, which influence the release of vesicle content^8,9^ or degradation^10^. Following internalization, we observed that some supermeres were present in the endolysosomal compartments (Fig. 1f). We next investigated the biodistribution and organ uptake of these different fractions in vivo. We labelled sEVs, exomeres and supermeres with near-infra-red dye and injected these into the peritoneum of C57BL/6 mice. The signal intensity was greatest in the kidney, liver and spleen of the supermere-injected mice; however, uptake was also high in the lung, colon, bone and heart (Fig. 1g). Although we observed little uptake of sEVs and exomeres in the brain, as previously reported^2^, the uptake of supermeres in the brain was significant (Fig. 1g), which suggests that supermeres can cross the blood–brain barrier.

Given that there was considerable uptake of supermeres in the lung and heart, we investigated related proteins in supermeres. We recently identified ACE2—the receptor for severe acute respiratory syndrome coronavirus 2 (SARS-CoV-2)—in sEVs and exomeres^11^. We found that supermeres derived from lung cancer (Calu-3) and CRC (LIM1215 and DiFi) cell lines had similar levels of ACE2 as exomeres (Fig. 1h and Extended Data Fig. 1f). The peptidase ACE was also present in supermeres from cell lines and plasma. ACE is a central component of the renin–angiotensin system that controls blood pressure but it also functions in innate and adaptive immunity^12^ (Fig. 1h and Extended Data Fig. 1f). Another cardiovascular-related protein, PCSK9, is a circulating serine protease that degrades low-density-lipoprotein receptors, regulating the circulating levels of low-density lipoprotein^13^. PCSK9 showed a similar distribution to ACE2 and ACE in the fractions isolated from Calu-3, LIM1215 and DiFi cells (Fig. 1h and Extended Data Fig. 1f). Together, these findings demonstrate that supermeres are secreted nanoparticles with a distinct morphology. These nanoparticles circulate in vivo, are efficiently taken up in multiple organs and contain cargo relevant to cardiovascular disease.

### Supermeres have distinct proteomes with high levels of TGFBI

**Fig. 2:**
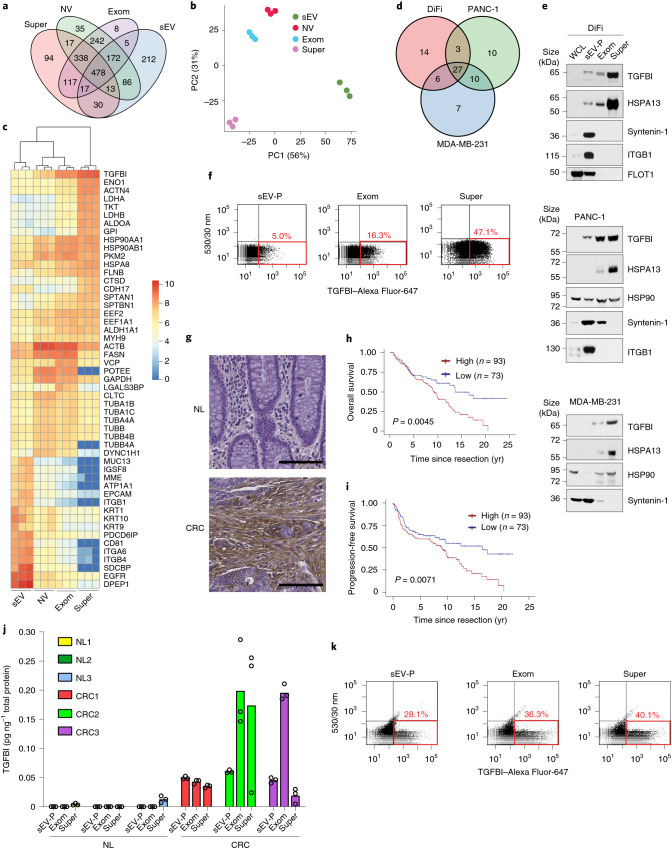
Supermeres exhibit a distinct proteome with high levels of TGFBI. **a**, Venn diagram of unique and common proteins identified in DiFi-derived sEVs, NV fractions, exomeres and supermeres. **b**, Principal component (PC) analysis of normalized DiFi proteomic mass spectral counts. **c**, Heatmap of the top-20 most abundant proteins in each of the samples from DiFi cells. **d**, Venn diagram of unique and common top-50 most abundant proteins identified in supermeres derived from DiFi, PANC-1 and MDA-MB-231 cells. **e**, Immunoblot of representative proteins in DiFi- (top), PANC-1- (middle) and MDA-MB-231-derived (bottom) supermeres. Equal quantities (30 µg) of protein from each fraction were analysed. **f**, FAVS analysis of the TGFBI levels in the sEV-P (left), exomeres (middle) and supermeres (right) derived from DiFi cells. **g**, Immunohistochemical staining of TGFBI expression in normal (NL) colon and CRC tissue samples. Data are representative of three independent experiments. Scale bars, 100 µm. **h**,**i**, Overall (**h**) and progression-free (**i**) survival analysis of patients with CRC with different levels of TGFBI (that is, high versus low) using the Kaplan–Meier method; data were compared between the two marker groups using a two-sided log-rank test. **j**, ELISA analysis of the TGFBI levels in supermeres derived from plasma from control individuals (NL1–3) and patients with CRC. Data are the mean of *n* = 3 technical replicates. **k**, FAVS analysis of the TGFBI levels in sEV-Ps, exomeres and supermeres derived from the plasma of patients with CRC. **f,k**, The red boxes indicate TGFBI-positive particles. The percentages indicate the percent of particles that contain TGFBI above the detection limit. WCL, whole-cell lysate; exom, exomere; and super, supermere. Source data

**Extended Data Fig. 2:**
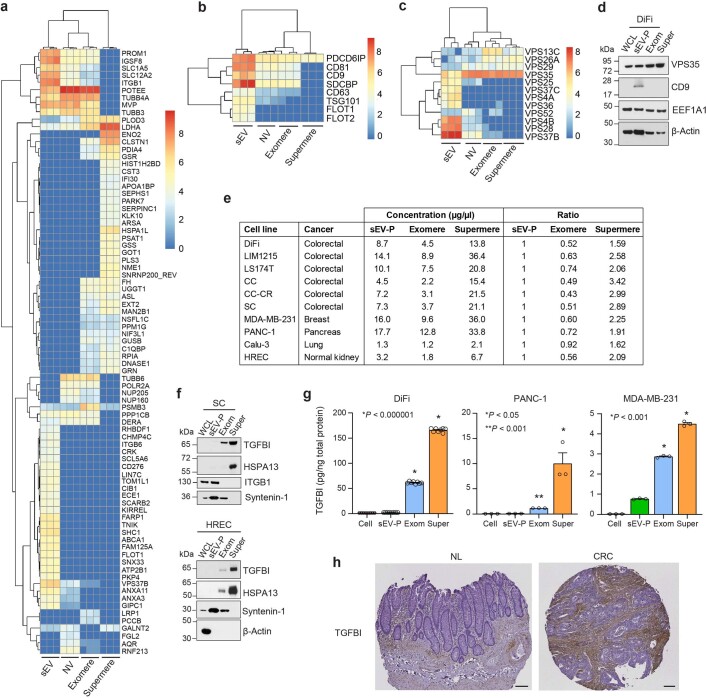
Supermeres exhibit distinct proteomic profiles. **a**, Heatmap of top-25 differentially expressed proteins in sEVs, NV, exomeres and supermeres from DiFi cells, based on normalized spectral counts. **b**,**c**, Heatmap of the relative abundance of select conventional sEV markers (**b**) and vacuolar protein sorting proteins (VPS) in sEVs, NV, exomeres and supermeres from DiFi cells (**c**). **d**, Immunoblot analysis of VPS35 in DiFi cells (WCL), the sEV-P, exomeres (exom) and supermeres (super). **e**, Protein concentrations and ratios of the sEV-P, exomeres and supermeres produced from cell lines in equal volumes. Note that the size of the sample preparations (number of cell culture plates) is not equal between different cell lines. **f**, Immunoblot analysis of SC and HREC cells (WCL), the sEV-P, exomeres and supermeres. **g**, ELISA analysis of TGFBI levels in DiFi, PANC-1 and MDA-MB-231 cells, the sEV-P, exomeres and supermere. Data are mean ± s.e.m. *n* = 8 for DiFi, and *n* = 3 for PANC-1 and MDA-MB-231. **h**, Immunohistochemical staining of TGFBI expression in normal colon (NL) and colorectal cancer (CRC) tissue samples. Representative images are shown. Scale bar, 100 µm. Source data

We performed liquid chromatography-coupled tandem mass spectrometry (LC–MS/MS) on gradient-purified sEVs, NV fractions, exomeres and supermeres. The proteomic profile of supermeres was distinct from that of sEVs, NV fractions and exomeres, with NV fractions and exomeres showing a marked overlap (Fig. 2a,b and Supplementary Table 1). For the top-20 most abundant proteins in each of the samples (Fig. 2c) and for the top-25 most differentially expressed proteins (Extended Data Fig. 2a), supermeres were highly enriched in proteins involved in metabolism, whereas classical exosomal markers were enriched in sEVs (Extended Data Fig. 2a,b and Supplementary Table 1). Exomeres, NV fractions and supermeres had a marked enrichment of retromer-complex components—VPS35, VPS29 and VPS26A—which mediate retrograde transport of cargo proteins (Extended Data Fig. 2c,d). Across all the different cell types, the yield of supermeres was higher than that of sEVs and the exomere yield was the lowest (Extended Data Fig. 2e). TGFBI was the most abundant protein identified in DiFi supermeres and the second-most abundant in PANC-1 supermeres, whereas the glycolytic enzyme ENO1 was the most abundant in the PANC-1 and MDA-MB-231 supermeres (Fig. 2c,d and Supplementary Table 2,3). The presence of TGFBI in supermeres was confirmed by immunoblotting (Fig. 2e and Extended Data Fig. 2f), ELISA (Extended Data Fig. 2g) and fluorescence-activated vesicle sorting (FAVS) analysis (Fig. 2f). The heat shock protein HSPA13 was enriched in supermeres from DiFi, PANC-1, MDA-MB-231, SC and human renal epithelial (HREC) cells, suggesting that HSPA13 may be a useful marker protein for supermeres (Fig. 2e and Extended Data Fig. 2f). The heat shock protein HSP90 was highly abundant in supermeres but less specific than HSPA13 (Fig. 2c,e and Supplementary Table 3). We next examined TGFBI immunohistochemical staining in a clinically well-annotated CRC TMA. Compared with normal colonic tissue, TGFBI immunoreactivity was greatly increased in CRC, predominantly in the stroma (Fig. 2g and Extended Data Fig. 2h). The overall (Fig. 2h) and progression-free (Fig. 2i) survival was lower for patients whose CRC tumours had high TGFBI staining in a clinically well-annotated TMA compared with those with low levels of TGFBI, as determined by Kaplan–Meier survival analysis. Higher levels of TGFBI (determined by ELISA) were found in all extracellular fractions isolated from the plasma of three patients with CRC compared with those from control individuals (Fig. 2j). TGFBI was detected by FAVS analysis in supermeres isolated from the plasma of an individual with CRC (Fig. 2k). In summary, supermeres display distinct proteomic profiles and TGFBI may be a potential biomarker for CRC.

### Supermeres increase lactate and transfer drug resistance

**Fig. 3:**
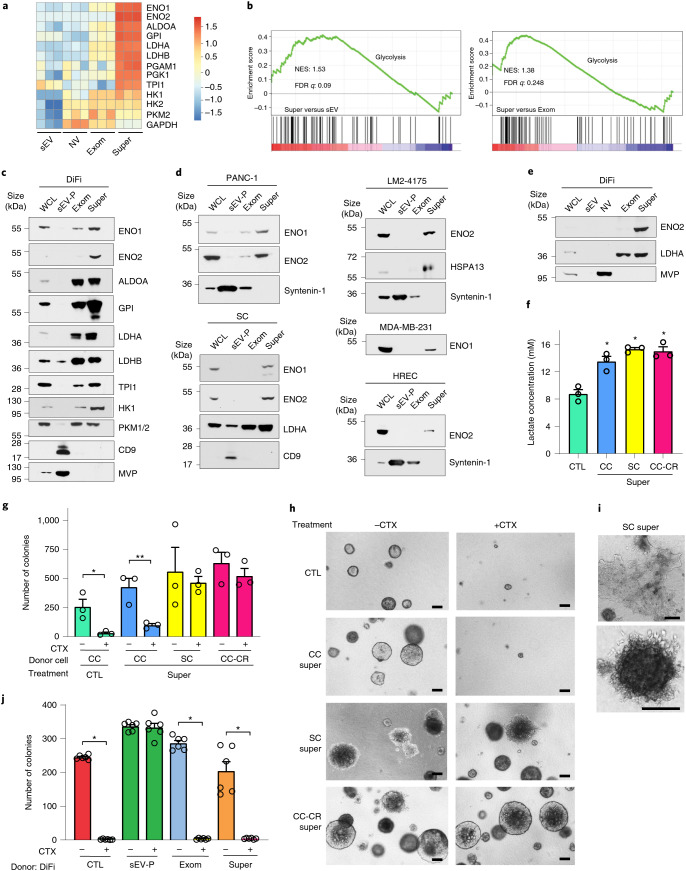
Supermeres increase lactate release and transfer cetuximab resistance. **a**, Heatmap of normalized spectral counts for select proteins and enzymes involved in glycolysis in sEVs, NV fractions, exomeres and supermeres from DiFi cells. **b**, GSEA analysis of pathways enriched in metabolic enzymes for supermeres versus sEVs (left) and supermeres versus exomeres (right) from DiFi cells. NES, normalized enrichment score; and FDR, false discovery rate. **c**,**d**, Immunoblot analysis of select metabolic enzymes and proteins involved in glycolysis in cells and extracellular samples derived from DiFi (**c**) as well as PANC-1, SC, LM2-4175, MDAM-MB-231 and HREC (**d**) cells. **e**, Immunoblot analysis of ENO2 and LDHA in DiFi whole-cell lysate as well as high-resolution density gradient-fractionated sEVs, NV fractions, and exomeres and supermeres. **c**–**e**, Equal quantities (30 µg) of protein from each fraction were analysed. **f**, Lactate release of CC cells treated with PBS (control) or 50 µg ml^−1^ supermeres derived from CC, SC or CC-CR cells as the mean ± s.e.m. of *n* = 3 independent treatments. **g**, Growth analysis of CC colonies in 3D collagen and treated with 50 µg ml^−1^ supermere derived from CC, SC or CC-CR cells in the presence or absence of cetuximab for 14 d. Colony counts plotted as the mean ± s.e.m. of *n* = 3 independent samples. **h**, Representative images of CC colonies from **g**. **i**, Representative low (top) and high (bottom) magnification images of CC colonies treated with SC supermeres. **h**,**i**, Scale bars, 200 µm. **j**, Growth analysis of DiFi colonies in 3D collagen and treated with 50 µg ml^−1^ sEV-Ps, exomeres and supermeres derived from DiFi cells in the presence or absence of cetuximab for 14 d. Colony counts plotted as the mean ± s.e.m. of *n* = 6 independent experiments. **f**,**g**,**j**, *P < 0.01, **P < 0.001; two-tailed Student’s *t*-test. Exom, exomere; super, supermere; WCL, whole-cell lysate; CTL, control; and CTX, cetuximab. Source data

**Extended Data Fig. 3:**
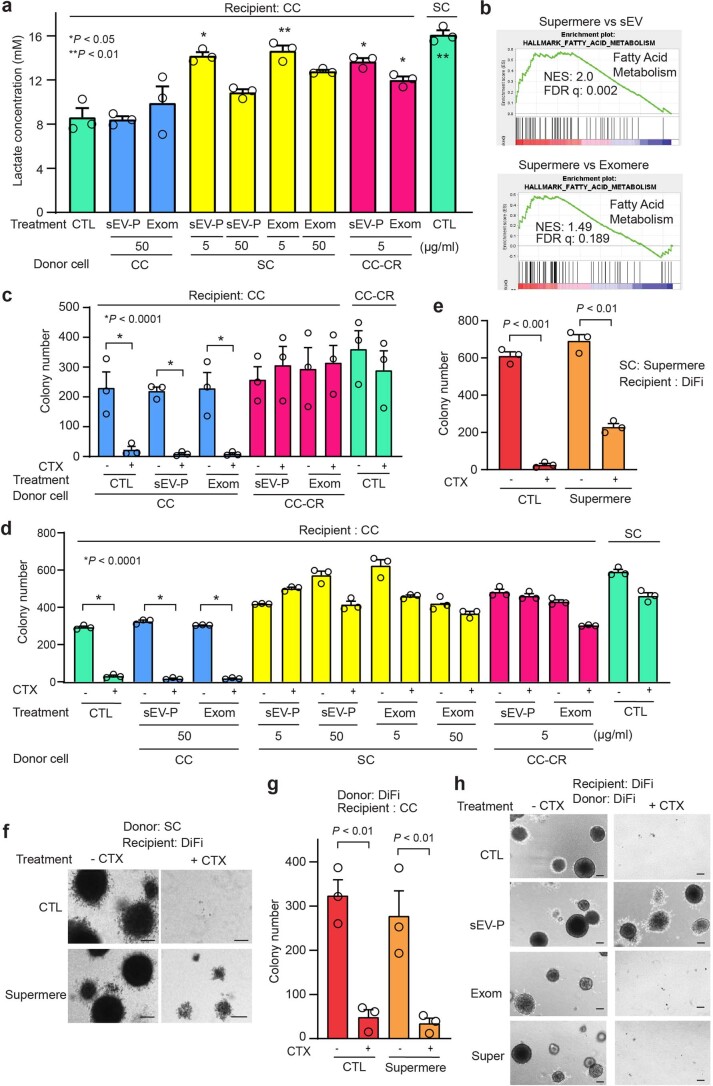
Supermeres increase lactate release and transfer drug resistance. **a**, Lactate release from CC cells treated with PBS (CTL), or 5 or 50 µg/ml of the sEV-P or exomeres derived from CC, SC or CC-CR cells is plotted. Data are mean ± s.e.m. *n* = 3 biological replicates. **b**, GSEA analysis of pathways enriched in metabolic enzymes for supermeres versus sEVs (top) and supermeres versus exomeres (bottom) from DiFi cells **c**, CC colony growth analysis in 3D collagen treated with 50 µg/ml of CC or CC-CR-derived sEV-P or exomeres in the presence or absence of cetuximab (CTX) for 14 days. Colony counts are plotted (mean ± s.e.m). *n* = 3 biological replicates. **d**, CC colony growth analysis in 3D collagen treated with 5 or 50 µg/ml of the sEV-P or exomeres derived from CC, SC, or CC-CR cells in the presence or absence of CTX for 14 days. Colony counts are plotted (mean ± s.e.m). *n* = 3 biological replicates. **e**, DiFi colony growth analysis in 3D collagen treated with 25 µg/ml of supermeres derived from SC cells in the presence or absence of CTX for 14 days. Colony counts are plotted (mean ± s.e.m). *n* = 3 biological replicates. **f**, Representative images of DiFi colonies from (**e**). Scale bar, 200 µm. **g**, CC colony growth analysis in 3D collagen treated with 25 µg/ml of supermeres derived from DiFi cells in the presence or absence of CTX for 14 days. Colony counts are plotted (mean ± s.e.m). *n* = 3 biological replicates. *P < 0.01 (two-tailed *t*-test). **h**, Representative images of DiFi colonies from (Fig. 3j). Scale bar, 200 µm. Source data

We previously reported that mutant KRAS exosomes derived from CRC cells can alter the metabolic state of the tumour microenvironment^14^. Given that glycolytic enzymes were enriched in supermeres (Fig. 2c and Supplementary Table 3), we examined the metabolic machinery further. Enrichment analysis of proteins that were differentially expressed revealed that many enzymes involved in glycolysis were highly enriched in supermeres compared with sEVs and exomeres (Fig. 3a,b) in addition to enzymes involved in fatty-acid metabolism (Extended Data Fig. 3b). ENO2 in particular was highly associated with supermeres (Fig. 3c–e). The marked enrichment of glycolytic enzymes prompted us to examine whether supermeres could alter the metabolism of recipient cells by increasing lactate release, a hallmark of the Warburg effect^15^. Treatment with supermeres derived from cystic colony (CC), cetuximab-resistant CC (CC-CR)^16^ and SC cells greatly increased lactate secretion in CC cells (Fig. 3f). Furthermore, the SC and CC-CR cell-derived sEV-Ps and exomeres also increased lactate release in CC cells (Extended Data Fig. 3a), indicating that release of both EVs and nanoparticles can influence the tumour microenvironment.

Increased lactate secretion has been linked to epidermal growth factor receptor (EGFR) and MET drug resistance^17^. Initially, we tested the ability of supermeres from cetuximab-resistant cells (SC and CC-CR) to transfer resistance to cetuximab-sensitive cells (CC) cultured in a three-dimensional (3D) environment of type-1 collagen^7,16^. After exposure to CC cell-derived supermeres, CC cells in 3D culture remained sensitive to the growth-inhibition effects of cetuximab (Fig. 3g,h). In contrast, after exposure to SC or CC-CR supermeres, the growth of CC cells was no longer inhibited by cetuximab (Fig. 3g,h). Transfer of cetuximab resistance was also observed when CC cells were treated with sEV-Ps and exomeres from SC and CC-CR cells (Extended Data Fig. 3c,d). The addition of supermeres from SC and CC-CR cells led CC colonies to morphologically resemble the donor cell (Fig. 3h). Some of the CC colonies treated with SC cell-derived supermeres displayed a spreading or migratory phenotype and others exhibited multiple protrusions (Fig. 3i). Furthermore, SC supermeres also transferred cetuximab resistance to highly cetuximab-sensitive DiFi cells (Extended Data Fig. 3e,f). DiFi supermeres failed to confer resistance to CC cells treated with cetuximab (Extended Data Fig. 3g). As expected, neither exomeres nor supermeres from DiFi cells conferred cetuximab resistance to DiFi cells (Fig. 3j and Extended Data Fig. 3h); however, addition of DiFi sEV-Ps did confer resistance to DiFi cells treated with cetuximab (Fig. 3j and Extended Data Fig. 3h).

In summary, supermeres are functional nanoparticles enriched in glycolytic enzymes. They can increase lactate release in recipient cells and are able to transfer drug resistance.

### Supermeres are enriched in shed membrane proteins

**Fig. 4:**
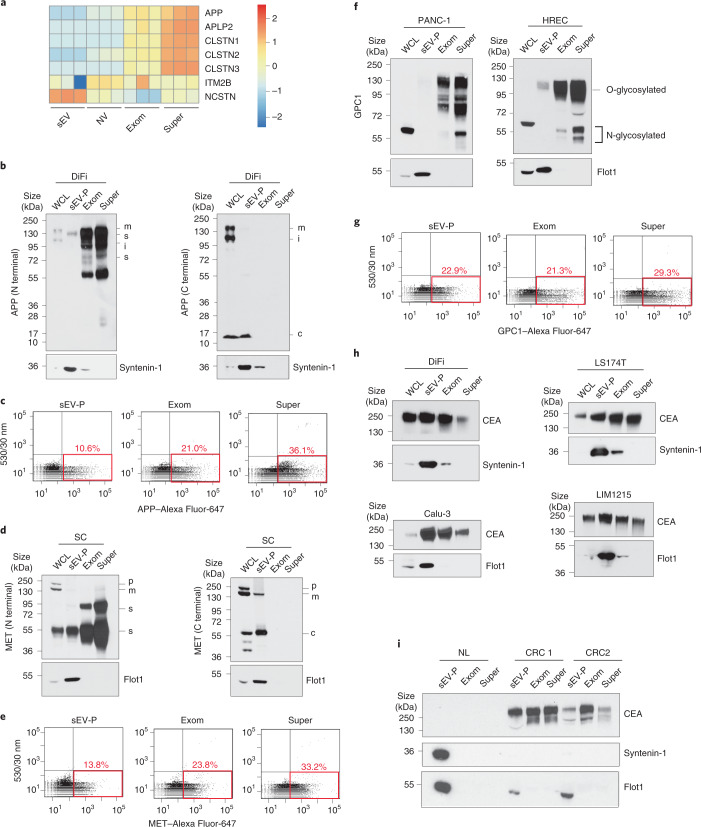
Supermeres are enriched in shed membrane proteins. **a**, Heatmap of normalized spectral counts of APP and other select membrane proteins involved in Alzheimer’s disease. **b**, Immunoblot analysis of APP in the whole-cell lysate, sEV-P as well as exomeres and supermeres of DiFi cells using N-terminal (left) and C-terminal (right) APP antibodies. c, C-terminal APP fragment; i, immature APP; m, mature APP; and s, soluble APP. **c**, FAVS analysis of APP in the sEV-P (left), exomeres (middle) and supermeres (right) of DiFi cells. **d**, Immunoblot analysis of MET in SC cells and corresponding extracellular samples using both N-terminal (left) and C-terminal (right) MET antibodies. c, C-terminal MET fragment; p, pro-form MET; m, mature MET; s, soluble MET. **e**, FAVS analysis of MET in the DiFi sEV-P, exomeres and supermeres using MET antibody directly conjugated to Alexa Fluor-647. **f**, Immunoblot analysis of GPC1 in the whole-cell lysate, sEV-P, exomeres and supermeres derived from PANC-1 (left) and HREC (right) cells using a rabbit monoclonal antibody. **g**, FAVS analysis of GPC1 in the sEV-P (left), exomeres (middle) and supermeres (right) of DiFi cells. **h**, Immunoblot analysis of CEA in whole-cell lysates, sEV-Ps, exomeres and supermeres derived from DiFi (top left), LS174T (top right), LIM1215 (bottom right) and Calu-3 (bottom left) cells. **i**, Immunoblot analysis of CEA in the sEV-Ps, exomeres and supermeres isolated from control individuals (NL) and plasma from patients with CRC. **c,e,g**, The red boxes indicate APP-, GPC-1- or MET-positive particles, respectively. The percentages indicate the percent of particles that contain APP, GPC-1 or MET, respectively, above the detection limit. **b**,**d**,**f**,**h**,**i**, Equal quantities (30 µg) of protein from each fraction were analysed. Exom, exomere; super, supermere; WCL, whole-cell lysate. Source data

**Extended Data Fig. 4:**
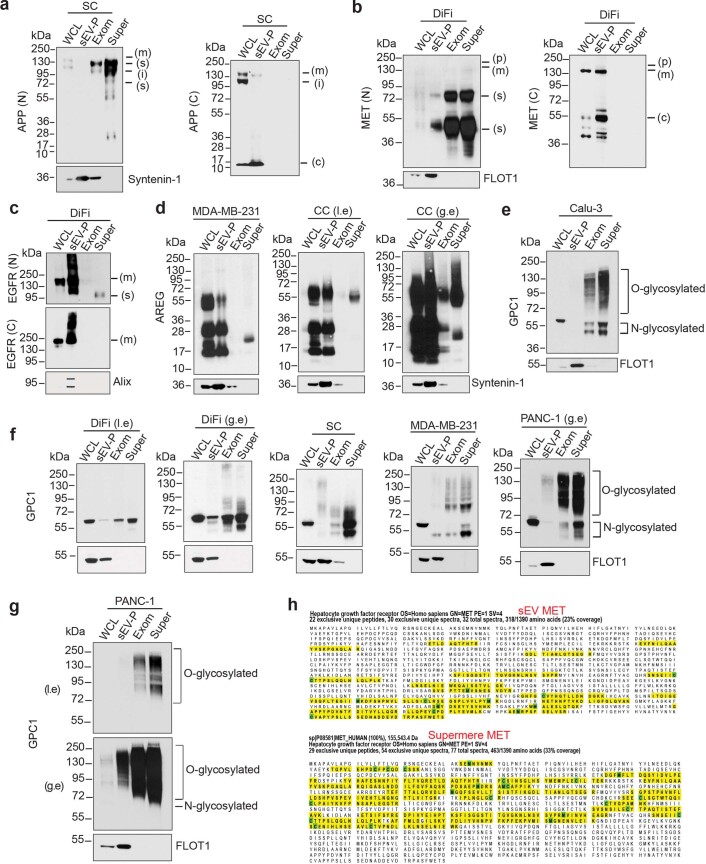
Supermeres and exomeres are highly enriched with clinically relevant shed membrane proteins. **a**, Immunoblot analysis of APP in SC cells, the sEV-P, exomeres (exom) and supermeres (super) using N-terminal (N, left) and C-terminal (C, right) APP antibodies. (i), immature APP; (m), mature APP; (s), soluble APP. **b**, Immunoblot analysis of MET in DiFi cells, the sEV-P, exomeres and supermeres, using N-terminal (N, left) and C-terminal (C, right) MET antibodies. (c), C-terminal fragment MET; (p), pro-form MET; (s), soluble MET. **c**, Immunoblot analysis of EGFR in DiFi cells, the sEV-P, exomeres and supermeres, using N-terminal (N) and C-terminal (C) EGFR antibodies. m, membrane; s, soluble. **d**, Immunoblot analysis of AREG in MDA-NB-231 cells and the sEV-P, exomeres and supermeres (left), and in CC cells and the sEV-P, exomeres and supermeres with short (left) and long exposure (right), using an N-terminal AREG antibody. g.e, greater exposure; l.e, lower exposure. **e**, Immunoblot analysis of GPC1 in Calu-3 cells, the sEV-P, exomeres and supermeres using a rabbit monoclonal GPC1 antibody. **f**, Immunoblot analysis of GPC1 in DiFi, SC, MDA-MB-231 and PANC-1 cells, and the sEV-P, exomere and supermere fractions, using a rabbit monoclonal GPC1 antibody. The PANC-1 immunoblot is a longer exposure of the corresponding membrane in Fig. 4f. **g**, Immunoblot analysis of GPC1 in PANC-1 cells, and the sEV-P, exomeres and supermeres, using a rabbit polyclonal GPC1 antibody with short (upper) and long exposure (lower) of the immunoblot. **h**, MET sequence in DiFi-derived sEV and supermere identified by mass spectrometry. Source data

Given that there was a marked uptake of supermeres in the brain (Fig. 1g), we examined APP, as carboxy (C)-terminal fragments of this protein have been reported in exosomes^18^. The transmembrane precursor protein APP is cleaved by secretases to generate soluble APPs, C-terminal fragments and amyloid beta, essential to the pathogenesis of Alzheimer’s disease^19^. APP and other Alzheimer’s disease-associated membrane proteins underwent ectodomain shedding and were highly enriched in supermeres (Fig. 4a and Supplementary Table 1). The enrichment of ectodomain APP in exomeres and supermeres derived from both DiFi and SC cells as well as the confinement of full-length APP to cells and sEVs was confirmed by immunoblotting with antibodies specific to ectodomain (amino (N)-terminal) or cytoplasmic (C-terminal) epitopes (Fig. 4b and Extended Data Fig. 4a). Enrichment of APP in supermeres was further confirmed by FAVS analysis (Fig. 4c).

MET, a receptor tyrosine kinase that is dysregulated in many cancers^20^, has been proposed to increase the metastatic behaviour of primary tumours via exosomes^21^. Proteomics data indicated that full-length MET was present in sEVs, whereas only peptides covering the ectodomain were present in supermeres and exomeres (Extended Data Fig. 4h). Immunoblotting with antibodies specific to ectodomain (N-terminal) or cytoplasmic (C-terminal) epitopes of MET revealed that the shed ectodomain of MET was highly enriched in supermeres released from SC (Fig. 4d) and DiFi cells (Extended Data Fig. 4b), whereas full-length MET was only detected in cells and sEVs. These results were confirmed with FAVS analysis (Fig. 4e). Shed ectodomains of EGFR were observed in supermeres released from DiFi cells (Extended Data Fig. 4c) and the ectodomain of the EGFR ligand amphiregulin (AREG) was observed in supermeres from MDA-MB-231 and CC cells (Extended Data Fig. 4d).

GPC1 is a GPI-anchored heparan sulfate proteoglycan that is overexpressed in several cancers, including pancreatic cancer and CRC, and exosomal GPC1 in the plasma is reported to be a sensitive and specific biomarker for the early detection of pancreatic cancer^22,23^. However, we found that different forms of GPC1 were far more associated with exomere and supermere nanoparticles released from pancreatic cancer cells (PANC-1) and normal HRECs (Fig. 4f). Similar results were observed for the Calu-3, DiFi, SC and MDA-MB-231 cell lines (Extended Data Fig. 4e–g). Validation was obtained by FAVS analysis (Fig. 4g). CEA, another GPI-anchored protein, is used in the clinic as a biomarker to monitor tumour recurrence in CRC patients^24^. CEA was present in sEVs, exomeres and supermeres derived from DiFi, LS174T, LIM1215 and Calu-3 cells (Fig. 4h). Furthermore, CEA was highly enriched in plasma sEVs, exomeres and supermeres from patients with CRC but was not detected in plasma from control individuals (Fig. 4i).

In summary, exomere and supermere nanoparticles are enriched in many shed, clinically relevant, membrane proteins—including APP, MET, GPC1, CEA, EGFR, AREG, ACE and ACE2—and can be detected by optimized flow cytometry.

### Distinct expression of small exRNAs in supermeres

**Fig. 5:**
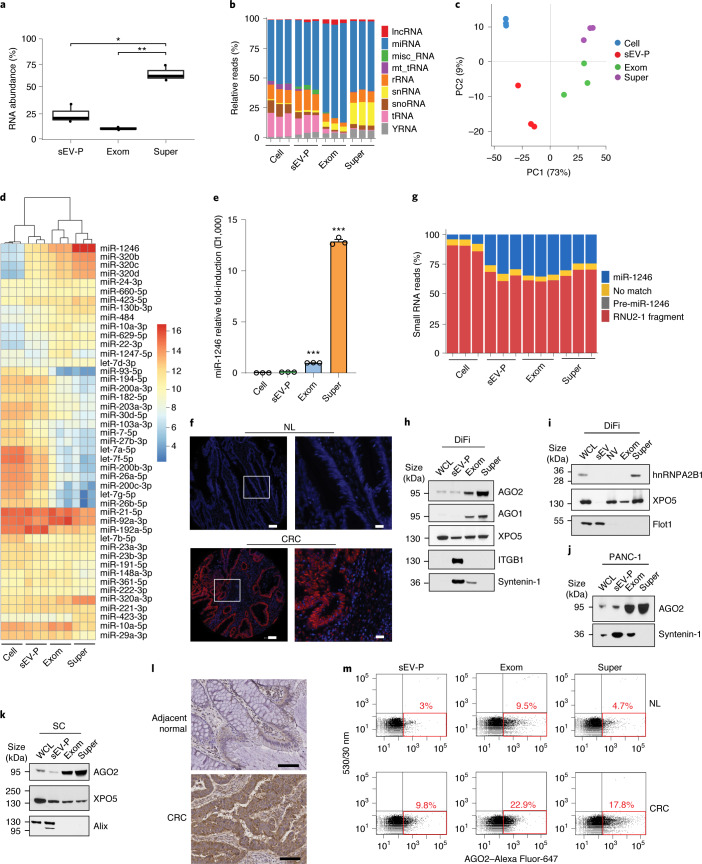
Distinct expression of small exRNAs in supermeres. **a**, Relative RNA abundance in the sEV-P, exomeres and supermeres of DiFi cells. Two-tailed Student’s *t*-test. For the boxplots, the centre lines mark the median, the box limits indicate the 25th and 75th percentiles, and the whiskers extend 1.5× the interquartile range from the 25th and 75th percentiles; *n* = 3 independent samples. **b**, Percentage of small-RNA reads mapped small noncoding RNA for DiFi cells, the sEV-P, exomeres and supermeres following RNA-seq. misc_RNA, miscellaneous RNA; mt_tRNA; mitochondrial tRNA; rRNA, ribosomal RNA; snoRNA, small nucleolar RNA; *n* = 3 independent samples. **c**, Principal component (PC) analysis of normalized miRNA reads for DiFi cells, the sEV-P, exomeres and supermeres following RNA-seq; *n* = 3 independent samples. **d**, Heatmap of the top-25 most abundant miRNAs across DiFi cells and extracellular compartments. **e**, Expression levels of miR-1246, determined by quantitative PCR with reverse transcription analysis, in DiFi cells and extracellular compartments. Data are the mean ± s.e.m. of *n* = 3 independent samples. Two-tailed Student’s *t*-test. **f**, Fluorescence in situ hybridization staining of miR-1246 in normal human colonic tissue (NL; top) and CRC (bottom) from a TMA. Representative data from three independent experiments are shown. Scale bar, 100 µm (left) and 20 µm (right; magnified view of the region in the white box). **g**, Percentage of normalized DiFi small-RNA reads containing the miR-1246 sequence. **h**–**k**, Immunoblots of representative RNA-binding proteins identified in extracellular compartments derived from DiFi (**h**,**i**), PANC-1 (**j**) and SC cells (**k**). Equal quantities (30 µg) of protein from each fraction were analysed. **l**, Immunohistochemical staining of AGO2 expression in adjacent normal colon and CRC samples. Representative images are shown. Scale bars, 100 µm. **m**, FAVS analysis of the AGO2 levels in the plasma of control individuals (NL) and patients with CRC. The red boxes indicate AGO2-positive particles. The percentages indicate the percent of particles that contain AGO2 above the detection limit. Representative results are shown. *n* = 3 independent experiments. Exom, exomere; super, supermere; WCL, whole-cell lysate. *P *<* 0.05, **P *<* 0.01 and ***P < 0.001. Source data

**Extended Data Fig. 5:**
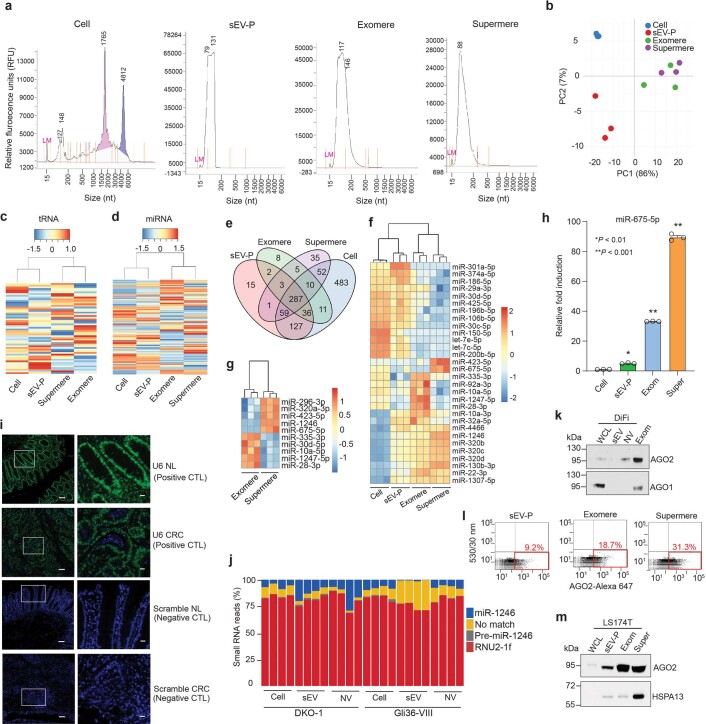
Characterization of small RNAs associated with different fractions. Bioanalyzer size profile of RNAs isolated from DiFi cells, the sEV-P, exomeres and supermeres. **b**, Principal component analysis of tRNAs in DiFi cells, the sEV-P, exomeres and supermeres. **c**, Heatmap of tRNA analysis in DiFi cells, the sEV-P, exomeres and supermeres. **d**, Heatmap of miRNAs analysis in DiFi cells, the sEV-P, exomeres and supermeres. **e**, Venn diagram of miRNAs identified in DiFi cells, the sEV-P, exomeres and supermeres. *n* = 3 biological replicates. **f**, Heatmap of top 10 differentially expressed miRNAs. Scale bar indicates intensity. DESeq2 was used to detect differential expression among samples. **g**, Heatmap of top 5 differentially expressed miRNAs in DiFi exomeres and supermeres. Scale bar indicates intensity. **h**, qRT-PCR analysis of miR-675-5p expression in DiFi cells, the sEV-P, exomeres and supermeres relative to U6. The mean Ct value for miR-675-5p and U6 are displayed in the table. Data are mean ± s.e.m. *n* = 3 biological replicates. **i**, Representative FISH staining of positive control of U6 (green) and negative control (CTL) of scrambled miRNA (green) in human normal tissue (NL) and colorectal cancer (CRC) tumours on a tissue microarray with DAPI (blue). Scale bars, 100 µm (left) and 20 µm (right). **j**, Percentage of normalized small RNA reads containing the miR-1246 sequence in cells, sEVs and NV fraction derived from DKO-1 and Gli36 vIII cells (dataset from Jeppesen et al. 2019, https://www.cell.com/cell/article/S0092-8674(19)30212-0/fulltext). **k**, Immunoblot analysis of AGO1 and AGO2 expression in DiFi cells, sEVs, NV and exomeres. WCL, whole-cell lysate; sEV, small extracellular vesicle; NV, non-vesicular; exom, exomere. **l**, FAVS analysis of AGO2 expression in the DiFi sEV-P, exomeres and supermeres. **m**, Immunoblot analysis of AGO2 expression in LS174T cells, sEVs, NV and exomeres. Source data

We next examined the RNA content of cells and extracellular carriers. Extracellular vesicle-associated exRNAs, especially microRNAs (miRNAs), have attracted attention due to their diverse biological functions and potential as cancer biomarkers^1,25–27^. The relative abundance of exRNAs in supermeres was significantly higher than in exomeres and the sEV-P (Fig. 5a). The small exRNAs associated with DiFi cells and their extracellular compartments displayed distinct patterns of distribution (Extended Data Fig. 5a,b). Among the RNA populations, miRNAs were the dominant RNA species (Fig. 5b), with exomeres containing the highest relative level of miRNAs (79%). A high percentage of transfer RNA (tRNA) was seen for cells and sEV-Ps (Fig. 5b and Extended Data Fig. 5c). Supermeres displayed a distinctive small exRNA repertoire with a relatively high percentage of small nuclear RNAs (snRNAs) compared with exomeres, sEV-Ps and cells. Supermeres exhibited distinct miRNA profiles (Extended Data Fig. 5d and Supplementary Table 4) and some miRNAs were detected solely in one extracellular carrier type (Extended Data Fig. 5e). The miRNA expression patterns of exomeres and supermeres were closely related but distinct from cells and sEV-Ps (Fig. 5c). Examination of the top-ten differentially expressed miRNAs revealed that some miRNAs were mostly present in cells with limited secretion (Extended Data Fig. 5f). The most highly abundant and enriched miRNAs in exomeres included miR-92a-3p, miR-1247-5p and miR-10a-5p (Fig. 5d and Extended Data Fig. 5f,g). The expression of supermere-enriched miR-1246 and miR-675 was validated (Fig. 5e, Extended Data Fig. 5h and Supplementary Table 5).

By far, the most abundant and most differentially expressed miRNA in supermeres (as determined by RNA sequencing (RNA-seq)) was miR-1246, with a 1,024-fold change in expression levels compared with cells (Extended Data Fig. 5g and Supplementary Table 4). Overexpression of miR-1246 has been observed in several cancer types, including lung and liver^28,29^. To investigate the clinical relevance of miR-1246, we performed fluorescence in situ hybridization of miR-1246 in CRC tumour and normal tissues on a TMA. The expression of miR-1246 was predominantly nuclear in tumour and stromal cells (Fig. 5f and Extended Data Fig. 5i). In normal epithelial cells, staining was either weak or undetectable. The strong nuclear miR-1246 staining in CRC tissue is consistent with it originating from cleavage of spliceosomal U2 snRNA^30^. Many other small RNAs in supermeres are derived from the extended RNU2 family, which includes many gene copies and pseudogenes. Despite the divergence of sequences among the RNU2 family members, the miR-1246 sequence is conserved in many family members apart from RNU2-1 (Supplementary Table 6). Furthermore, the majority of the miR-1246 sequences detected in both cells and extracellular compartments were derived from RNU2-1, and not from the proposed miR-1246 precursor (89 and 68% in the cells and supermeres, respectively; Fig. 5g), consistent with previous datasets (Extended Data Fig. 5j) and reports^31^. Given that the proposed pre-miR-1246 sequence was undetectable in both DiFi cells and extracellular compartments, our data thus support a previous finding that exosomal miR-1246 is generated through a Drosha- and Dicer-independent pathway^31^. The mature miR-1246 sequence reads were highly abundant in supermeres compared with cells (Fig. 5g and Supplementary Table 7), which provides further support for miR-1246 serving as a potential exRNA biomarker.

Several mechanisms have been proposed for sorting miRNAs into exosomes. The RNA-binding proteins, Y-box protein 1 (YBX1), sumoylated hnRNPA2B1 and argonaute proteins (AGO1–4) have all been reported to mediate exosomal mRNA secretion^32–34^. However, we and others have demonstrated that AGO1–4 are enriched in gradient-purified NV fractions and exomeres^1,3,26,35^. The observed abundance of RNAs in supermeres correlated with the proteomic data showing that supermeres were highly enriched in ribonucleoproteins, including argonaute proteins (Supplementary Table 1). AGO1 and AGO2 were enriched in DiFi cell-derived exomeres and supermeres but were not detected in high-resolution density gradient-purified sEVs (Fig. 5h, Extended Data Fig. 5k and Supplementary Table 1). Analysis by FAVS confirmed that the expression level of AGO2 in DiFi supermeres was higher than in the sEV-P (Extended Data Fig. 5l). AGO2 was also highly enriched in supermeres derived from PANC-1, SC and LS174T cells compared with cells and sEV-Ps (Fig. 5j,k and Extended Data Fig. 5m). CRC tissues displayed strong positive staining for AGO2 compared with the adjacent normal colonic mucosa (Fig. 5l). Furthermore, the level of AGO2 detected by FAVS in supermeres and exomeres isolated from the plasma of patients with CRC was higher than that from control individuals (Fig. 5m). Sumoylation of the ribonucleoprotein hnRNPA2B1 has been attributed to miRNA sorting into exosomes, including sorting of miR-1246 (refs. ^36,37^). However, hnRNPA2B1 was only detected in DiFi cells and supermeres (Fig. 5i and Supplementary Table 1), which suggests that the involvement of hnRNPA2B1 in miRNA sorting can be attributed to supermeres. Exportin-5 (XPO5) exports pre-miRNA from the nucleus to the cytoplasm^38^. XPO5 was enriched in extracellular NV fractions, exomeres and supermeres but was not detected in gradient-purified sEVs, suggesting that XPO5 may be involved in sorting of miRNAs to NV extracellular nanoparticles (Fig. 5h,i,k and Supplementary Table 1). Many known RNA-binding proteins^39,40^ were found to be enriched specifically in NV fractions, exomeres and supermeres rather than sEVs (Supplementary Table 8).

In summary, supermeres display a distinct signature of small exRNAs with very high expression of specific miRNAs, including miR-1246, and supermeres are enriched for the miRNA-binding proteins AGO1, AGO2, hnRNPA2B1 and XPO5. High levels of AGO2 secretion in exomeres and supermeres may be a common feature of cancer cells.

### Supermeres affect the levels of liver lipids and glycogen

**Fig. 6:**
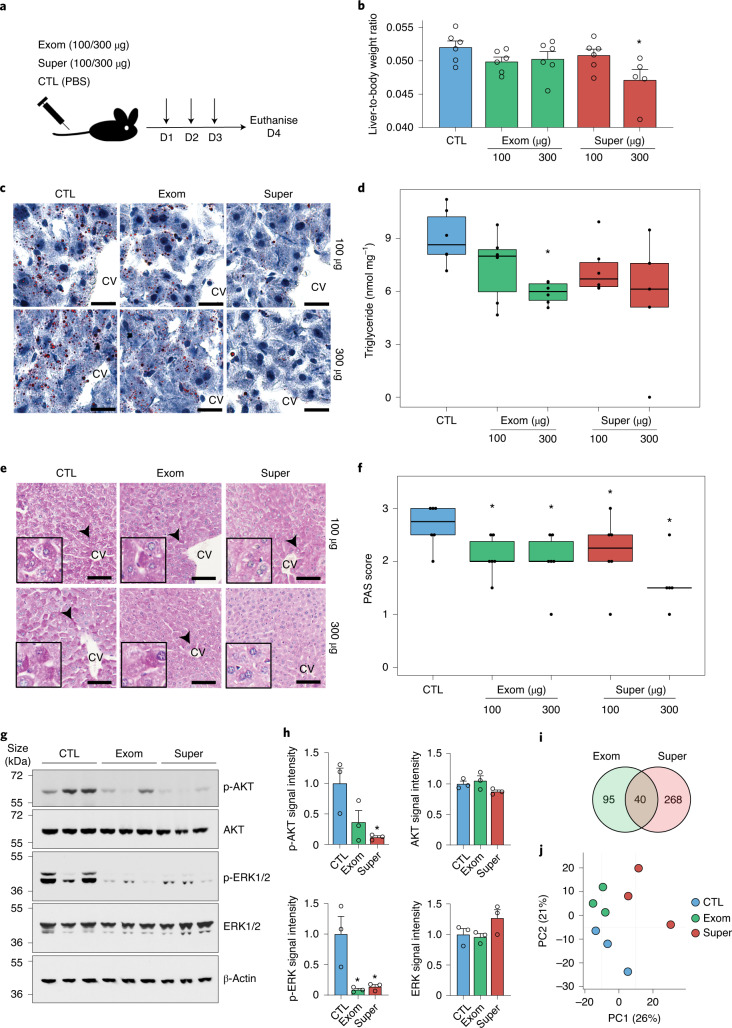
Supermeres affect the in vivo levels of liver lipids and glycogen. **a**, Schematic of the mouse treatment experiments. D, day. **b**, Liver-to-body weight ratio of mice following PBS, exomere or supermere treatments. Two-sided Kruskal–Wallis test, followed by Dunn’s post-hoc test. Data are the mean ± s.e.m. of *n* = 6 animals. **c**, Oil red O staining of mouse livers following three consecutive injections with PBS (left) or exomeres (middle) and supermeres (right) derived from DiFi cells. The livers were harvested 24 h after the last injection. Scale bars, 20 µm. **d**, Level of triglycerides in liver tissue following injection with exomeres or supermeres derived from DiFi cells. **e**, Periodic acid–Schiff staining of formalin-fixed paraffin-embedded (FFPE) liver tissue following injection with exomeres or supermeres derived from DiFi cells. There were significant differences between experimental groups by pathology scoring of hepatocytes containing darker magenta deposits of polysaccharides (arrowheads; *P* = 0.038, two-sided Kruskal–Wallis test). Representative images are shown. Inset: magnified view with a diameter of approximately 90 µm. Scale bars, 100 µm. **f**, Histological scoring of liver sections stained with periodic acid–Schiff (PAS). The sections were scored double-blinded (0–3) for intensity and homogeneity by two liver pathologists. The liver sections from the mice injected with 300 µg of supermeres showed decreased scores in comparison to the other treatment groups. **d**,**f**, Two-sided Wilcoxon rank-sum test; *n* = 6 animals. For the boxplots, the centre lines mark the median, the box limits indicate the 25th and 75th percentiles, and the whiskers extend 1.5× the interquartile range from the 25th and 75th percentiles. **g**, Immunoblot of select proteins in mouse liver lysates after treatment with PBS (control) or 300 µg of exomeres or supermeres. **h**, Levels of proteins detected by immunoblot. Data are the mean ± s.e.m. of *n* = 3 animals. One-way ANOVA, followed by Holm–Bonferroni correction. **i**, Venn diagram of unique and common genes that are differentially expressed compared with the control (PBS) group between exomere- and supermere-treated mice. The criteria for inclusion of a differentially expressed gene were fold change > 1.5 and FDR < 1.0. **j**, Principal component (PC) analysis of gene expression in the mouse liver cells following treatment. Exom, exomere; super, supermere; CV, centrilobular vein; and CTL, control. *P < 0.05. Source data

**Extended Data Fig. 6:**
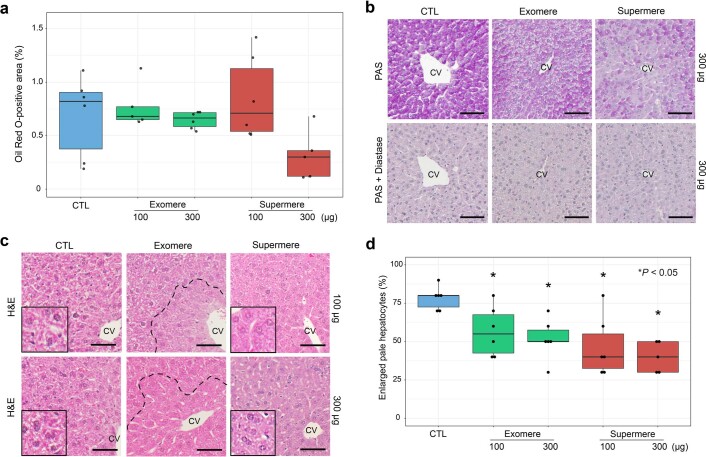
Organ biodistribution of supermeres and effects on liver *in vivo*. **a**, Histological scoring of liver sections stained with Red Oil O. Significance assessed by two-sided Wilcoxon rank-sum. *n* = 5–6 animals. For boxplots, the centre lines mark the median; box limits indicate 25th and 75th percentiles; whiskers extend 1.5 times the interquartile range from 25th and 75th percentiles. CTL, control. **b**, Periodic acid-Schiff (PAS) staining of formalin-fixed, paraffin-embedded (FFPE) liver tissue with or without glycogen digestion by diastase. CV, centrilobular vein. Scale bar, 100 µm. **c**, Hematoxylin and eosin (H&E) staining of FFPE liver tissue following injection with exomeres or supermeres derived from DiFi cells. PBS-injected control mice showed larger areas of enlarged hepatocytes with vacuolated cytoplasm, which extended to the edge of the CV. Exomere and supermere-injected mice had a reduction of these large hepatocytes in the centrilobular area as delimited by the hyphenated line, when compared to the PBS control groups (two-sided Kruskal–Wallis, *P* = 0.01). Enlarged inset diameter, approximately 68 µm. Scale bar, 75 µm. **d**, Histological scoring of liver H&E sections for the percentage of enlarged hepatocytes. For boxplots, the centre lines mark the median; box limits indicate 25th and 75th percentiles; whiskers extend 1.5 times the interquartile range from 25th and 75th percentiles. *n* = 5–6 animals. The most significant reduction was between the supermere 300 µg group and the PBS controls. *P < 0.05 (two-sided Wilcoxon rank sum). Source data

Given that supermeres were enriched for proteins involved in metabolism (Fig. 3a–e), with the liver as a major target for supermere biodistribution (Fig. 1g), we examined the acute effects on the liver following systemic delivery of supermeres. Mice were injected with supermeres or exomeres via the tail vein (Fig. 6a). No gross effects on the liver were observed, but a supermere-selective decrease in the liver-to-body ratio was observed (Fig. 6b). We subsequently found a reduction in the number and size of hepatic lipid droplets following injection with both supermeres and exomeres (Fig. 6c and Extended Data Fig. 6a) as well as a trend towards lower triglyceride concentrations in liver tissue (Fig. 6d). Following supermere or exomere treatment, hepatocytes also displayed a significant reduction in glycogen levels (Fig. 6e,f and Extended Data Fig. 6b). Whereas control mice exhibited uniformly pale, large hepatocytes, blinded scoring confirmed a significant reduction in enlarged and pale hepatocytes in the mice that were treated with supermeres and exomeres (Extended Data Fig. 6c,d), especially around the centrilobular veins that comprise metabolic zone 3, which is particularly active in glycolysis and lipogenesis^41^. AKT and ERK1/2 signalling are known to regulate glucose and lipid metabolism^42,43^. In accordance with the in vivo observations, there was a significant reduction in phosphorylated (p)-AKT and p-ERK1/2 in liver cells following supermere treatment (Fig. 6g,h). We performed RNA-seq analysis of whole liver tissue, and gene set enrichment analysis (GSEA) revealed that exomere and supermere injections significantly downregulated cholesterol homeostasis, fatty-acid metabolism, oxidative phosphorylation and adipogenesis pathways, potentially accounting for the effects we observed in the liver (Supplementary Tables 9 and 10). Interestingly, both the supermere- and exomere-treated mice also had a marked downregulation of hepatic mTORC1 signalling, a major nutrient-sensitive regulator of growth^44^. Despite these overall similarities, there were significant differences in gene expression between the two groups (Fig. 6i,j), suggesting selectivity of these effects. In summary, exomeres and supermeres have potent and distinct effects on hepatic glucose and lipid metabolism, probably by modulation of AKT and ERK1/2 signalling.

### DPEP1 and CD73 are potential CRC biomarkers in exosomes

**Fig. 7:**
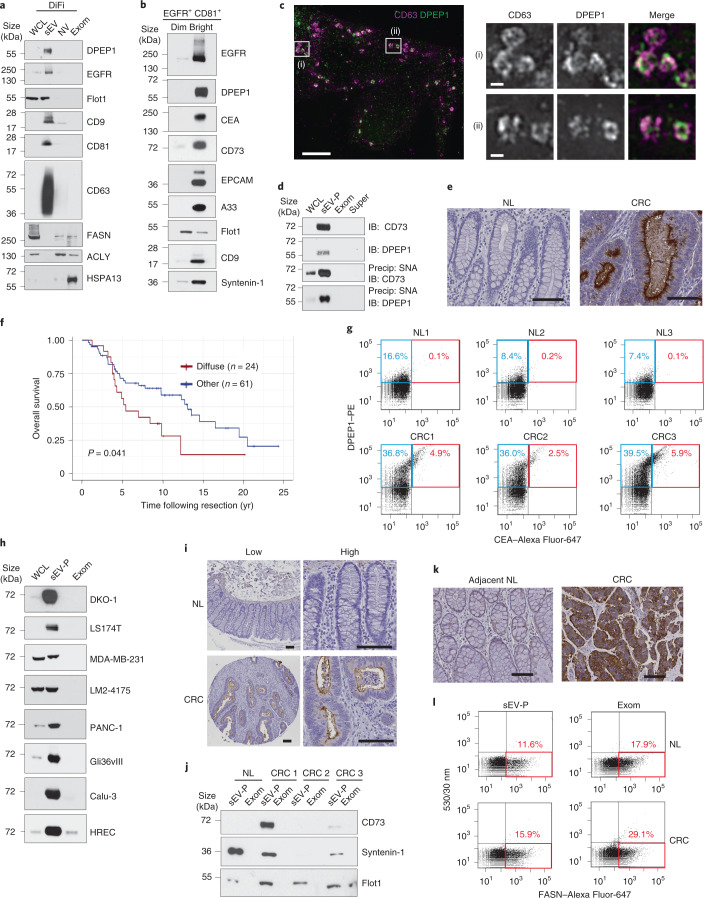
DPEP1 and CD73 are potential CRC biomarkers in exosomes. **a**, Immunoblot of representative proteins identified in the whole-cell lysates, sEVs, NV fractions and exomeres of DiFi cells. **b**, Immunoblot of representative proteins identified in sEVs sorted by FAVS based on the expression of EGFR and CD81. The same number of sorted vesicles (1.5 × 10^6^) were analysed for each sample. **c**, Localization of endogenous CD63 and DPEP1 in DiFi cells imaged using 3D structured illumination microscopy (SIM). 1.8 µm *z*-stack projection (left). Magnified views of the regions in the white squares are shown (right). Data are representative of two independent experiments. Scale bars, 5 µm (left) and 500 nm (right). **d**, Level of α2,6-sialylated DPEP1 and CD73 detected in the whole-cell lysates, sEV-Ps, exomeres and supermeres of DiFi cells. IB, immunoblot; precip, precipitation. **e**, Immunohistochemical staining of DPEP1 expression in normal colon (NL) and CRC tissue samples. Data are representative of three independent experiments. Scale bar, 100 µm. **f**, Overall survival analysis of patients with CRC comparing their DPEP1-staining patterns (diffuse versus others) using the Kaplan–Meier method; data were compared between marker groups using a two-sided log-rank test. **g**, FAVS analysis of the levels of DPEP1 and CEA in the sEV-Ps from the plasma of control individuals and patients with CRC using anti-DPEP1 directly conjugated to phycoerythrin (PE). The blue boxes indicate DPEP1^+^ sEVs and the red boxes indicate DPEP1^+^CEA^+^ double-positive sEVs. **h**, Immunoblot analysis of CD73 expression in cells (whole-cell lysates), sEV-Ps and exomeres from different cell lines. **i**, Immunohistochemical staining of CD73 expression in normal colon and CRC tissue samples. Low (left) and high (right) magnification images. Data are representative of three independent experiments. Scale bars, 100 µm. **j**, Immunoblot analysis of CD73 in the sEV-P and exomeres isolated from plasma samples of control individuals and patients with CRC. **k**, Immunohistochemical staining of FASN expression in adjacent normal colon and CRC tissue samples. Data are representative of three independent experiments. Scale bars, 100 µm. **l**, FAVS analysis of the FASN levels in the sEV-Ps and exomeres of plasma from normal controls and patients with CRC using anti-FASN directly conjugated to Alexa Fluor-647. **a,d,h,j**, Equal quantities (30 µg) of protein from each fraction were analysed. The red boxes indicate FASN-positive particles. The percentages indicate the percent of particles that contain FASN above the detection limit. WCL, whole-cell lysate; exom, exomere; and super, supermere. Source data

**Extended Data Fig. 7:**
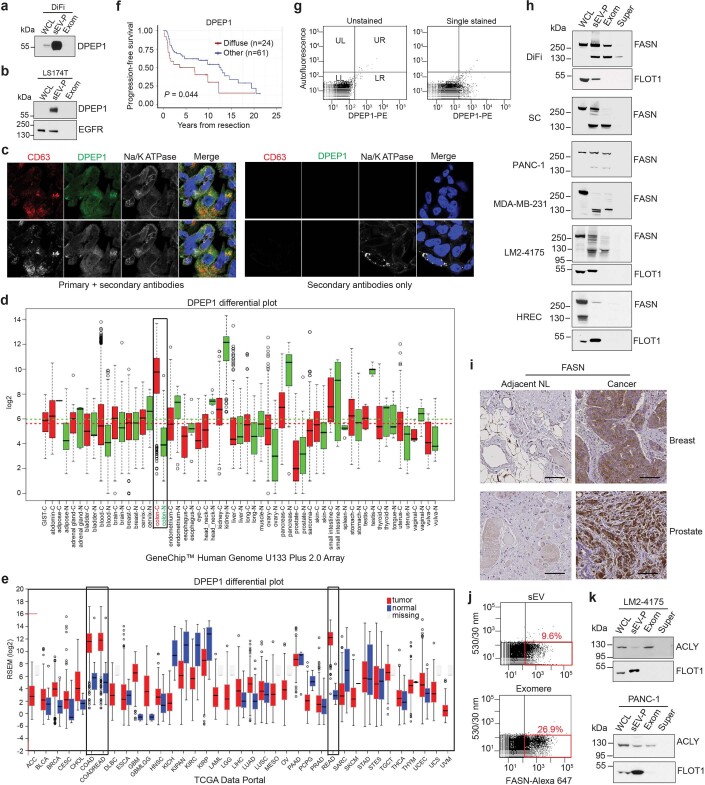
DPEP1 in exosomes and FASN in exomeres are potential CRC biomarkers. **a**,**b**, Immunoblot of DPEP1 expression in DiFi (**a**) and LS174T (**b**). **c**, Localization of endogenous CD63 and DPEP1 in DiFi cells imaged with confocal microscopy. Left panels: primary + secondary antibodies. Right panels: secondary antibodies only control. Scale bar, 10 µm. **d**, DPEP1 expression from microarray platform U133 plus 2.0 (http://gent2.appex.kr/gent2/). Data were presented by box plots, where the centre line shows the median, the bounds of the box show the first and third quantile, whiskers extend to the most extreme values within 1.5 interquartile range (1.5 *IQR), and dots denote outliers reaching past 1.5 interquartile range. *n* = 3775 for biologically independent colon cancer samples, *n* = 397 for biologically independent normal colon samples. **e**, DPEP1 expression from TCGA RNA-seq (http://firebrowse.org/viewGene.html). Data were presented by box plots, where the centre line shows the median, the bounds of the box show the first and third quantile, whiskers extend to the most extreme values within 1.5 interquartile range (1.5*IQR), and dots denote outliers reaching past 1.5 interquartile range. *n* = 458 for COAD_tumor, *n* = 41 for COAD_normal, *n* = 625 for COADREAD_tumor, *n* = 51 for COADREAD_normal, *n* = 167 for READ_tumor, and *n* = 10 for READ_normal biologically independent samples. **f**, Progression-free survival analysis of CRC patients comparing DPEP1 staining pattern (diffuse versus others) using Kaplan and Meier, using two-sided log-rank test. **g**, Gating strategy. Greater than 98% of the unstained samples that fell within the lower left (LL) quadrant of a dot-plot were used as negative control (baseline). Samples that fell in the lower right (LR) quadrant were considered as epitope positive, while samples falling in the LL quadrant were below the limit of detection. This gating panel corresponds to Figs. 2f, 2k, Figs. 4c, 4e, 4g, Fig. 5m, Figs. 7g, 7l, Extended Data Fig. 5l. **h**, Immunoblot analysis of FASN expression. **i**, Immunohistochemical staining of FASN expression in adjacent normal (NL) and cancer tissue samples of breast and prostate. Scale bar, 100 µm. **j**, FAVS analysis of FASN level. **k**, Immunoblot analysis of ACLY. Source data

We then identified which proteins are most abundant in the sEVs and exomeres of DiFi cells. DPEP1—a GPI-anchored zinc-dependent dipeptidase involved in glutathione metabolism, regulation of leukotriene activity^45^ and neutrophil recruitment^46^—and EGFR were the two most abundant proteins in gradient-purified DiFi sEVs (Fig. 7a, Extended Data Fig. 7a and Supplementary Table 1). They were also present in the sEV-P derived from LS174T cells (Extended Data Fig. 7b), despite low expression in cell lysates. To determine whether DPEP1 was present in classical exosomes^1^, we sorted sEVs by FAVS with fluorescently labelled antibodies to EGFR and the exosomal marker CD81 (Fig. 7b). The double-stained populations were analysed and sorted into EGFR^+^CD81^+^ bright or dim subpopulations^3^. Notably, DPEP1 as well as known CRC biomarkers (CEA, EPCAM and A33) were highly enriched in the EGFR^+^CD81^+^ bright population. CD73 (also known as NT5E)—a GPI-linked 5′-ecto-nucleotidase that converts AMP to immunosuppressive adenosine and is overexpressed in CRC^47,48^—was also highly enriched in this population (Fig. 7b). Conversely, FLOT1 was more enriched in the EGFR^+^CD81^+^ dim populations, suggesting that FLOT1 is more associated with a different subset of exosomes and/or non-exosomal sEVs. These results underscore the heterogeneity of sEVs and the utility of FAVS for analysis and sorting of distinct vesicle populations. DPEP1 co-localized with the canonical exosome marker CD63 in multivesicular endosomes (Fig. 7c and Extended Data Fig. 7c), further validating the presence of DPEP1 in classical exosomes. Moreover, we determined that DPEP1 and CD73 were α2,6-sialylated (Fig. 7d).

Next, we examined the clinical relevance of DPEP1 as a potential CRC biomarker. Bioinformatic analysis of the U133 plus 2.0 and The Cancer Genome Atlas databases showed that DPEP1 was highly upregulated in CRC compared with normal colonic tissue (Extended Data Fig. 7d,e). Immunohistochemical analysis of clinically well-annotated TMAs of CRC revealed that DPEP1 staining was markedly increased in CRC but undetectable in normal colonic mucosa (Fig. 7e). Cox regression analysis showed a significant inverse correlation between CRCs with diffuse cytoplasmic staining for DPEP1 and the overall (Fig. 7f) as well as progression-free survival of patients (Extended Fig. 7f). Using FAVS, we demonstrated that sEVs double-positive for DPEP1 and CEA were much higher in the plasma from patients with CRC compared with the controls, suggesting that DPEP1 may be a promising biomarker and target for a subset of patients with CRC (Fig. 7g and Extended Data Fig. 7g).

Furthermore, sEVs derived from human cancer cell lines—DKO-1 and LS174T, MDA-MB-231 and its derivative LM2-4175, PANC-1, Gli36vIII and Calu-3—as well as normal HRECs had high levels of CD73 (Fig. 7h). This observation is supported by previous studies^1,2,49^ and suggests that CD73 is a potential marker protein for sEVs. Immunohistochemical staining of CD73 in CRC tissues showed increased membranous and cytoplasmic CD73 immunoreactivity in the tumour compared with the adjacent normal colonic mucosa (Fig. 7i). CD73 was detected in the sEV-Ps isolated from the plasma of two patients with CRC but was not present in the third patient or in the control individual (Fig. 7j).

We then set out to examine proteins enriched in exomeres and the NV fraction. The most abundant proteins detected in DiFi-derived exomeres and the NV fraction were β-actin and fatty-acid synthase (FASN; Figs. 2c, 7a and Supplementary Table 1). FASN was also expressed in exomeres and NV fractions released from other cell lines (Extended Data Fig. 7h). FASN catalyses the synthesis of palmitate from acetyl-CoA and malonyl-CoA^50^. Strong immunohistochemical staining for FASN was observed in CRC but was absent in the adjacent normal mucosa (Fig. 7k). FASN staining was higher in breast and prostate tumours compared with the adjacent normal mucosa (Extended Data Fig. 7i). To assess whether FASN could be detected in exomeres, we first showed that it is highly enriched in exomeres from DiFi cells by FAVS (Extended Data Fig. 7j) and then, as proof-of-principle, we detected higher levels of FASN in exomeres isolated from the plasma of a patient with CRC compared with a control (Fig. 7l). In addition to FASN, other enzymes related to lipogenesis were enriched in exomeres and the NV fraction, including ACLY, ACSS2, ACACA and IDH1 (Supplementary Table 1). ACLY catalyses the conversion of citrate and coenzyme A to acetyl-CoA, which is a central metabolite for de novo fatty-acid and cholesterol biosynthesis. High expression of ACLY was confirmed in the NV fraction and exomeres derived from DiFi, LM2-4175 and PANC-1 cells (Fig. 7a and Extended Data Fig. 7k).

Thus, we have identified DPEP1 and CD73 in classical exosomes, as well as FASN in exomeres, to be potential CRC biomarkers and druggable targets. These results highlight the benefits of parsing distinct extracellular compartments to identify biomolecules of clinical interest and to assign cargo to their correct carrier.

## Discussion

Heterogeneity of EVs and nanoparticle populations is a major challenge in the EV field^1–5^. Here we report the isolation and characterization of a distinct extracellular nanoparticle that we have termed supermere. Supermeres are distinct from exomeres in terms of size, morphology, composition, cellular-uptake dynamics and tissue distribution. Our current AFM and electron microscopy data do not allow us to define structural differences between exomeres and supermeres beyond the diameter, height and volume differences identified by fluid-phase AFM. Efforts are ongoing to examine these nanoparticles by cryogenic electron microscopy to determine their structural differences more precisely. In tissue biodistribution experiments, we consistently found greater final uptake of supermeres in vivo compared with sEVs, despite the slower uptake kinetics of supermeres (and exomeres) in vitro. An explanation for this discrepancy may reflect important differences in how cells interact with nanoparticles versus sEVs or merely be due to the technical limitations of our experiments. Future studies will be needed to resolve this issue.

Supermeres contain many proteins that have previously been reported to be associated with exosomes^5^. For example, TGFBI, the most abundant protein in supermeres, is purportedly a component of EVs from mesenchymal stromal cells^51^. Based on our findings that patients with CRC whose tumours exhibit high TGFBI immunoreactivity have a poor outcome and that the levels of TGFBI, determined by ELISA, are markedly increased in sEV-Ps, exomeres and supermeres isolated from the plasma of patients with CRC compared with those isolated from control individuals, we propose that TGFBI may be a useful marker in liquid biopsies for patients with CRC. TGFBI has been linked to both cancer cell migration^52^ and an immunosuppressive tumour microenvironment^53^. TGFBI mediates binding to extracellular-matrix proteins such as collagen and fibronectin, and can interact with integrin proteins^52,53^. Future studies will focus on whether TGFBI-associated supermeres are responsible for these effects. Argonaute proteins, including AGO1 and AGO2, were presumed exosomal proteins but refinements in purification demonstrate that these miRNA-binding proteins are predominantly NV^1,35^ and associated with supermeres. Other known RNA-binding proteins are also enriched in supermeres, highlighting that a significant proportion of exRNAs and RNA-binding proteins are not associated with EVs^1,54^. Many miRNAs that are barely detectable or undetectable at the cellular level are highly and selectively enriched in supermeres. For example, miR-1246, which has been linked to serum exosomes in patients with CRC^37^, is the most highly expressed and highly enriched miRNA in supermeres. The strong staining of miR-1246 in CRC tissue compared with normal colonic mucosa supports miR-1246 as a biomarker with potential roles in the pathogenesis of CRC.

It is important to note that supermeres and exomeres are not the only NV extracellular nanoparticles capable of transporting miRNA as high-density lipoprotein (HDL) particles in plasma and serum are known to contain miRNA^55,56^. All the cell line-derived supermere samples generated for this work were from serum-free conditions with no detection of ApoA1 or ApoA2 (the most abundant proteins of HDL complexes) by proteomic analysis. However, efficient purification from HDL-rich blood may benefit from additional approaches, perhaps utilizing a combination of high-resolution density gradient fractionation^1^ and fast protein liquid chromatography or size-exclusion chromatography^55,56^ for improved separation of sEVs, exomeres, supermeres and HDL particles.

We demonstrated that supermeres and exomeres isolated from cetuximab-resistant SC and CC-CR cells can transfer cetuximab resistance to cetuximab-sensitive cells. Activation of the receptor tyrosine kinases MET and RON induce de novo cetuximab resistance in SC cells^7^. In CC-CR cells, upregulation of a long noncoding RNA (lncRNA), MIR100HG and two embedded miRNAs (miR-100 and miR-125b) is responsible for this acquired mode of cetuximab resistance^16^. Thus, multiple cargos, including proteins and RNA (messenger RNA, miRNA and lncRNA) carried by nanoparticles may contribute to these modes of drug resistance. The identity of these cargos, and whether they act independently or cooperatively in cetuximab resistance, await further investigation.

The Warburg effect features enhanced lactate secretion, acidification of the tumour microenvironment and extracellular-matrix degradation^15^. Lactate secretion has been linked to resistance to drugs targeting EGFR and MET^17^. We demonstrated that cancer cell-derived supermeres contain large amounts of glycolytic enzymes and their addition to recipient cells increases lactate secretion. Furthermore, treating mice with supermeres reduces the levels of lipids and glycogen in the liver. The liver phenotype we observed is similar to that reported with hepatic mTORC1 inhibition in which there was decreased hepatic steatosis and an increased inflammatory response^44^. Future studies will be needed to assign these effects on the liver to specific cargo in supermeres and exomeres.

Shedding or release of membrane receptors to the extracellular environment is associated with a number of disease states^57^ and drug resistance^58^. Secretion of full-length transmembrane receptors is, as we demonstrated, a distinctive feature of sEVs/exosomes but the ectodomain of many clinically relevant transmembrane receptors—including MET, GPC1, CEA, ACE, ACE2 and APP—are highly abundant in supermeres. As an example, the secreted receptor ACE2 in sEVs and extracellular nanoparticles may act as a decoy for SARS-CoV-2 to attenuate infection, as has been demonstrated for human soluble recombinant ACE2 (refs. ^11,59^). A GPI-anchor attached to the C terminus of a protein enables it to be attached to the plasma membrane of cells or EVs, and many GPI-anchored proteins of clinical importance—including GPC1, CEA, DPEP1 and CD73—have been detected in the extracellular space and ascribed to exosomes. However, GPC1 is less associated with exosomes, or other sEVs, but is instead enriched in exomeres and supermeres. Other GPI-anchored proteins (for example, DPEP1 and CD73) are strongly associated with EGFR^+^CD81^+^ exosomes. DPEP1 was recently identified as a neutrophil-binding receptor and targeting DPEP1 reduced mortality in murine models of sepsis, suggesting a role for DPEP1 in inflammation^46^. Here we demonstrated that increased diffuse DPEP1 staining is associated with overall and progression-free survival in CRC and increased levels of DPEP1^+^CEA^+^ exosomes are present in the plasma of patients with CRC. High levels of CD73 have been linked to immune suppression and tumour progression due to the generation of extracellular adenosine^60^. We found increased levels of CD73 in CRC tumour tissue and demonstrated that CD73^+^ exosomes can be detected in the plasma of patients with CRC.

Based on our findings, we propose that TGFBI, ENO1 and GPC1 may be useful markers for extracellular nanoparticles (exomeres and supermeres), whereas HSPA13 and ENO2 are more specifically associated with supermeres. Going forward, it will be important to elucidate the biogenesis of supermeres and exomeres. The abundance of retromer machinery associated with both of these amembranous nanoparticles may offer a clue. Equally important will be to unravel the mechanism(s) underlying the effects mediated by these extracellular nanoparticles and their cargo.

In summary, we have identified a distinct circulating extracellular nanoparticle. Supermeres are enriched in proteins and miRNAs central to a number of disease states, including cancer, COVID-19, cardiovascular disease and Alzheimer’s disease. Many of these proteins have previously been ascribed to exosomes or other sEVs. Our findings serve to highlight the importance of parsing the exact extracellular compartment that contains a biomolecule of interest. Supermeres are also functional agents of intercellular communication that are efficiently taken up by multiple organs, including the liver, lung, colon, heart and brain. Thus, supermeres take their place alongside EVs and exomeres as a rich source of circulating cargo for candidate biomarkers and therapeutic targets in a number of disease states.

## Methods

The research conducted as part of this manuscript complies with all of the relevant ethical regulations. The use of the human samples was approved by the Vanderbilt University Medical Center Institutional Review Board (IRB; IRB nos 161529 and 151721).

### Cell lines

The LS174T, PANC-1, Calu-3 and HeLa cell lines were obtained from the American Type Culture Collection. Human primary renal proximal tubule epithelial cells (HRECs) were from Innovative BioTherapies. The LIM1215 cell line was obtained from the Ludwig Institute for Cancer Research. The HCA-7 cell line was obtained from S. Kirkland (Imperial Cancer Research Fund); its derivatives (SC, CC and CC-CR) and the DiFi cell lines were developed in the Coffey laboratory. The DKO-1 cell line was obtained from T. Sasazuki at Kyushu University, Gli36 cells were obtained from X. Breakefield at Harvard Medical School, and MDA-MB-231 and LM2-4175 cells were obtained from J. Massagué at Memorial Sloan-Kettering Cancer Center. The cell lines were authenticated using short-tandem-repeat analysis. All cell lines tested negative for mycoplasma contamination (Universal mycoplasma detection kit, American Type Culture Collection).

### Cell culture

Human CRC; DiFi; DKO-1; HCA-7-derived SC^7^, CC, CC-CR; LS174T; LIM1215; MDA-MB-231 and LM2-4175 (human breast cell lines); PANC-1 (pancreatic cancer cell line); Calu-3 (lung cancer cell line); Gli36vIII (human glioblastoma cell line) and HeLa cells were cultured in DMEM medium supplemented with 10% bovine growth serum, 1% glutamine, 1% non-essential amino acids and 1% penicillin–streptomycin at 37 °C in a 5% CO_2_ humidified incubator. All cell culture media were purchased from Corning Cellgro and all cell culture supplements were from Hyclone, unless stated otherwise. Primary cultures for production of EVs were initiated at passage 2 and the cells were maintained in DMEM supplemented with 2 μg ml^-1^ normocin, insulin-transferrin-selenium, epidermal growth factor, hydrocortisone and T3 thyroid hormone. For the 3D cultures, the cells were cultured in type-1 collagen. Type-1 collagen was diluted at 2 mg ml^−1^ in DMEM containing 10% FBS. Assays were set up using three collagen layers, with the middle layer containing the single-cell suspension at 5,000 cells ml^−1^. Medium with or without reagents was added on top and changed every 2–3 d.

### EV and nanoparticle isolation from cells cultured in dishes

Extracellular nanoparticles were isolated from cell-conditioned medium as previously described^3^, with minor modifications. The colon, breast, lung, and pancreatic cells mentioned earlier were cultured until 80% confluent. The cells were then washed three times with PBS and cultured in serum-free medium for 48 h. For primary human kidney epithelial cells, cell-conditioned medium was collected from cells at approximately 95% confluency, which had been cultured for 96 h in cell culture flasks with DMEM without FBS. The serum-free conditioned medium was centrifuged for 15 min at 1,000*g* to remove cellular debris and the resulting supernatant was then filtered through a 0.22 μm polyethersulfone filter (Nalgene) to reduce microparticle contamination. The filtrate was concentrated using a centrifugal concentrator with a 100,000 molecular-weight cutoff (Millipore). The concentrate then was subjected to high-speed centrifugation at 167,000*g* for 4 h in a SW32 Ti swinging-bucket rotor (Beckman Coulter) and the resulting sEV pellet was resuspended in PBS containing 25 mM HEPES (pH 7.2) and washed by centrifuging again at 167,000*g* for 4 h. The washed pellet was designated as the sEV-P. To isolate exomeres, the supernatant collected from the 4 h ultracentrifugation was ultracentrifuged at 167,000*g* for 16 h. The resulting pellet was resuspended in PBS containing 25 mM HEPES (pH 7.2) and washed by centrifuging again at 167,000*g* for 16 h. The washed pellet was designated as exomeres. To isolate supermeres, the supernatant from the pelleting of exomeres was subjected to ultracentrifugation at 367,000*g* using a Beckman Coulter SW55 Ti rotor (*k* factor of 48, Beckman Coulter) for 16 h. The resulting pellet was resuspended in PBS containing 25 mM HEPES (pH 7.2) and was designated supermeres. A standard production lot of DiFi consisted of 80 culture dishes (15 cm) with approximately 1.34 × 10^8^ cells per dish at the time of harvest. The typical protein yield was approximately 4 mg sEV-P, 2.5 mg exomeres and 7 mg supermeres.

### EV and nanoparticle isolation from cells cultured in bioreactors

DKO-1 cells were maintained in CELLine Adhere 1000 (CLAD1000) bioreactors (INTEGRA Biosciences AG) at 37 °C in a 5% CO_2_ humidified incubator. Cell-conditioned medium was harvested from bioreactors every 48 h, starting from 1 week after inoculation of the bioreactor and continuing for a period of 4 weeks. The sEV-Ps, exomeres and supermeres were isolated as described in the previous section of Methods.

### EV and nanoparticle isolation from human plasma samples

All procedures on human peripheral-blood specimens were approved and performed in accordance with the Vanderbilt University Medical Center IRB (IRB nos 161529 and 151721). All participants provided informed consent (clinical trial registration number: NCT03263429). The participants did not receive compensation. Consent to publish this information was provided. Blood was drawn into BD Vacutainer blood collection tubes (BD Bioscience) containing buffered sodium citrate as an anticoagulant. Plasma was generated by centrifugation of the blood at 1,500*g* for 15 min and then a second round of centrifugation of the supernatant at 3,000*g* for 15 min to ensure that no platelets remained. The resulting plasma samples were immediately diluted (approximately 1:20) in ice-cold PBS and centrifuged at 20,000*g* for 30 min to pellet and remove large EVs and microparticles. The sEV-Ps, exomeres and supermeres were generated as described earlier.

### High-resolution (12–36%) iodixanol density-gradient fractionation

Iodixanol (OptiPrep) density media (Sigma-Aldrich) were prepared in ice-cold PBS immediately before use to generate discontinuous (12–36%) step gradients. Crude sEV pellets were resuspended in ice-cold PBS and mixed with ice-cold iodixanol in PBS to obtain a final 36% iodixanol solution. The suspension was added to the bottom of a centrifugation tube and carefully overlaid with iodixanol in PBS, in descending order of concentration, yielding the complete gradient. The bottom-loaded 12–36% gradients were subjected to ultracentrifugation at 120,000*g* for 15 h at 4 °C using a SW41 Ti swinging-bucket rotor. Twelve fractions of 1 ml were collected from the top of the gradient. Fractions 4 and 5, and fractions 8 and 9 were separately pooled. These two pools were then diluted 12-fold in PBS and subjected to ultracentrifugation at 120,000*g* for 4 h at 4 °C. The resulting pellets were lysed in cell lysis buffer for further proteomic and immunoblotting analysis.

### AFM imaging and analysis

Twenty microlitres of isolated sEVs, NV fractions, exomeres and supermeres were diluted 1:1 with PBS and then incubated over (3-aminopropyl) triethoxysilane (AP)-modified mica substrates (Ted Pella Inc.) for 3 min. To remove unbound particles, the substrates were washed twice with 50 µl PBS and imaged in PBS at room temperature. Measurements were conducted in PBS using a Dimension FastScan microscope (Bruker Instruments) in off-resonance tapping mode, with ScanAsyst Fluid+ tips (Bruker) with a nominal radius of about 2 nm and experimentally determined spring constants of 0.7 N m^−1^. The AFM images were taken at 256 samples per line, at 0.75 Hz. The images were exported offline and processed using the Gwyddion or custom R software.

For statistical analysis, data were expressed as the mean ± s.d. Statistical significance was determined using the Student’s *t*-test for the differences between different samples. *P* values of less than 0.01 were considered to be statistically significant.

### Negative-stain TEM

Highly purified sEV fractions, NV fractions, exomeres and supermeres were prepared for TEM as previously described^1^. Extracellular sample fractions were prepared for TEM by absorption of samples onto carbon film (1–2 nm thick) mounted on carbon-coated holey-film grids for 5 min at 4 °C. This was accomplished by floating the grid on 25 μl of sample. Following sample adsorption, the grids were quickly and gently blotted on filter paper, immediately floated on 1 ml of 1% uranyl acetate at 4 °C for 5 min and then dried on filter paper. Imaging was performed on a JEM 1200EX microscope. Micrographs were captured with a BioScan 600 W digital camera (Gatan) using the DigitalMicrograph software (Gatan). In all cases, TEM was performed on a fresh sample of EVs that had not been subjected to freezing temperatures at any step in the purification or processing.

### Proteomics

Gradient-fractionated sEVs, NV fractions, exomeres and supermeres derived from DiFi, PANC-1 and MDA-MB-231 cells were lysed in RIPA buffer, and equal amounts of protein were run on a NuPAGE bis–Tris gel. LC–MS/MS was performed as previously described^2^. Briefly, Coomassie-stained gels were treated with 45 mM DTT for 30 min at 55 °C and carbamidomethylated for 30 min with 100 mM iodoacetamide at room temperature. The gels were destained and digested overnight with trypsin at 37 °C. Peptides were extracted with 60% acetonitrile, 0.1% trifluoroacetic acid, dried and reconstituted in 0.1% formic acid. The peptides were analysed by LC–MS/MS. An analytical column was packed with 20 cm of C18 reverse-phase material (Jupiter, 3 μm beads, 300 Å, Phenomenox) directly into a laser-pulled emitter tip. The peptides were loaded on the capillary reverse-phase analytical column using a Dionex Ultimate 3000 nanoLC and autosampler. The mobile-phase solvents consisted of 0.1% formic acid, 99.9% water (solvent A) and 0.1% formic acid, and 99.9% acetonitrile (solvent B). The peptides were gradient-eluted at a flow rate of 350 nl min^−1^ using a 180 min gradient. A Q Exactive Plus mass spectrometer (Thermo Scientific), equipped with a nano-electrospray ionization source, was used to mass analyse the eluting peptides using a data-dependent method. The instrument method consisted of MS1 using an MS AGC target value of 3 × 10^6^, followed by up to 20 MS/MS scans of the most abundant ions detected in the preceding MS scan. A maximum MS/MS ion time of 80 ms was used with a MS2 AGC target of 5 × 10^4^. The dynamic exclusion was set to 30 s, HCD collision energy was set to 27 normalized collision energy, and peptide match and isotope exclusion were enabled. For the identification of peptides, tandem mass spectra were searched using Sequest (Thermo Fisher Scientific) against a *Homo sapiens* database created from the UniprotKB protein database (https://www.uniprot.org/). The search results were assembled using Scaffold 4.3.2 (Proteome Software).

### Proteomic analysis

Proteins with an average count of ≥1 in each fraction were considered detectable. Spectral counts of proteins were normalized to the total spectral counts and log_2_-transformed. Principal component analysis was performed to assess the similarity between samples. Differential expression between sEVs, NV fractions, exomeres and supermeres was identified using Limma. Proteins with a fold change > 2 and a FDR < 0.05 were considered to be significantly differentially expressed. GSEA was implemented against three reference gene sets from the Molecular Signatures database (MSigDB v6.1; http://software.broadinstitute.org/gsea/msigdb/index.jsp): H, hallmark gene sets (50 gene sets); C2, Kyoto Encyclopedia of Genes and Genomes gene sets (186 gene sets); and C5, all gene ontology gene sets (5,917 gene sets). Default parameters were used to identify significantly enriched gene sets (minimum size, 15; maximum, size 500; and FDR < 0.25).

### SIM

A Nikon N-SIM structured illumination platform equipped with an Andor DU-897 EMCCD camera and a SR Apo TIRF ×100 (1.49 NA, WD 0.12) oil immersion objective was used for 3D SIM imaging and processing. Samples were imaged in PBS at room temperature. For calibration, 100 nm fluorescent (360/430 nm, 505/515 nm, 560/580 nm and 660/680 nm) beads (TetraSpeck Microspheres, Thermo Fisher Scientific) were fixed and imaged. The images were analysed using the ImageJ software (National Institutes of Health).

### Immunofluorescence staining for SIM

DiFi cells were cultured on 35 mm culture dishes with a 1.5 coverslip (P35G-0.170–14-C, MatTek Corporation). The cells were fixed with 4% paraformaldehyde in PBS at room temperature for 20 min and then extracted for 5 min with 1% Triton X-100 in 4% paraformaldehyde in PBS, as previously described^3^. The cells were washed three times in PBS and blocked in 10% BSA in PBS. The cells were incubated with primary antibodies diluted in 10% BSA at 4 °C overnight and washed three times with PBS. The secondary Alexa Fluor antibodies (anti-rabbit conjugated to Alexa Fluor-488 and anti-mouse conjugated to Alexa Fluor-568) were prepared in blocking buffer and centrifuged at 10,000*g* for 10 min before incubation with the cells for 1 h at room temperature. The primary antibodies used were: anti-DPEP1 (1:50; Sigma-Aldrich, HPA012783) and anti-CD63 (1:50; BD, 556019).

### Immunofluorescence staining for confocal microscopy

DiFi cells (2 × 10^5^) were cultured on six-well plates for 2 d. The cells were then washed with PBS, fixed with 4% paraformaldehyde for 10 min at room temperature and permeabilized with 0.5% Triton X-100 for 5 min at room temperature. The fixed cells were blocked in 5% BSA for 2 h at 4 °C and subsequently incubated at 4 °C overnight with primary antibodies in 5% BSA in PBS. The primary antibodies used were: anti-DPEP1 (1:100; Sigma-Aldrich, HPA012783), anti-CD63 (1:100; BD, clone H5C6, 556019) and Alexa Fluor-647 anti-sodium potassium ATPase (Na/KATPase; 1:500; Abcam, clone EP1845Y, ab198367).

The cells were washed three times in PBS and then incubated overnight with secondary antibodies in 5% BSA in PBS. The secondary antibodies used were: donkey anti–rabbit IgG (1:600; Invitrogen, A21206, Alexa Fluor-488 conjugated) and cy3. Immunofluorescence was analysed using a Zeiss LSM 710 confocal microscope. Microscopy was performed at the Vanderbilt Cell Imaging Shared Resource (CISR). All micrographs were taken using a ×63 oil immersion objective lens.

### Immunoblot analysis

Cells and all isolated fractions were lysed in ice-cold RIPA buffer: 50 mM Tris–HCl pH 7.5, 150 mM NaCl, 1% Triton X-100, 1% deoxycholate, 0.1% SDS and 1 mM PMSF containing a complete protease inhibitor tablet and a PhosSTOP tablet (Roche). The lysates were sonicated three times and then cleared by centrifugation at 14,000 r.p.m. for 5 min. The supernatant fractions were quantified using a Direct Detect system. The samples (30 µg) were separated on 4–12% SDS–PAGE bis–Tris gels (Life Technologies) under either reducing or non-reducing conditions, depending on the primary antibody, before being transferred to nitrocellulose membranes (GE Healthcare). The membranes were blocked for 1 h in 5% non-fat dry milk or 5% BSA, depending on the primary antibody used. The membranes were incubated with primary antibodies overnight at 4 °C. After incubation with secondary antibodies for 1 h, the immunoblots were developed using chemiluminescence (Western Lightning Plus-ECL, PerkinElmer).

The following primary antibodies were used. Anti-EEF1A1 (clone EPR9471; ab157455), anti-A33 (clone EPR4240; ab108938), anti-EPCAM (clone E144; ab32392), anti-AGO2 (clone EPR10411; ab186733), anti-Syntenin-1 (clone EPR8102; ab133267), anti-ACE2 (clone EPR4435(2); ab108252), anti-APP (clone Y188; ab32136), anti-GPC1 (clone EPR19285; ab199343), anti-CEACAM5/CEA (clone EPCEAR7; ab133633), anti-TPI1 (ab96696), anti-LDHB (clone 60H11; ab85319), anti-GPI (clone 1B7D7; ab66340), anti-HSPA8 (clone EP1531Y; ab51052), anti-PCSK9 (clone EPR7627(2); ab181142), anti-VPS35 (clone EPR11501(B); ab157220) and anti-MVP (clone EPR13227(B); ab175239), all from Abcam. Anti-MET (clone D1C2; 8198), anti-CEACAM5/CEA (clone CB30; 2383), anti-CD73 (clone D7F9A; 13160), anti-FASN (clone C20G5; 3180), anti-ACLY (4332), anti-AGO1 (clone D84G10; 5053), anti-XPO5 (clone D7W6W; 12565), anti-HNRNPA2B1 (clone 2A2; 9304), anti-Alix (clone 3A9; 2171), anti-ALDOA (clone D73H4; 8060), anti-ENO1 (3810), anti-ENO2 (clone D20H2; 8171), anti-HK1 (clone C35C4; 2024), anti-PKM1/2 (clone C103A3; 3190), anti-LDHA (clone C4B5; 3582), anti-pAKT (9271), anti-AKT (9272), anti-p-ERK1/2 (9101), anti-ERK1/2 (9102) and anti-HSP90 (clone C45G5, 4877) from Cell Signaling Technology. Anti-HSPA13 (clone A-11; sc-398297), anti-ACE (clone E-9; sc-271860), anti-FASN (clone G-11; sc-48357) and anti-CD9 (clone C-4, SC-13118) from Santa Cruz Biotechnology. Anti-APP (clone 22C11; MAB348), anti-β-actin (clone AC-74; A5316), anti-DPEP1 (HPA012783) and anti-EGFR (06-847) from Sigma-Aldrich. Anti-GPC-1 (Invitrogen, PA5-28055), anti-MET (AF276) and anti-CD81 (clone 454720; MAB4615) from R&D Systems. Anti-AREG (6R1C2.4) from Bristol-Myers Squibb Research Institute. Anti-TGFBI (10188-1-AP) from Proteintech. Anti-FLOT1 (clone 18; 610820), anti-β1-integrin (clone 18/CD29; 610467) and anti-CD63 (clone H5C6; 556019) from BD Transduction Laboratories. All of the antibodies were used at a 1:1,000 dilution, except anti-Synteinin-1 and anti-β-actin which were used at 1:5,000.

### ELISA for TGFBI

The concentrations of TGFBI in the sEV-Ps, exomeres and supermeres derived from human cancer cell lines and human platelet-poor plasma were determined using an ELISA kit (R&D Systems, DY29350) according to the manufacturer’s instructions.

### FAVS staining, sorting and analysis

The sEV-Ps derived from DiFi cells were stained and sorted as previously described^3,61^. Briefly, 5 mg of DiFi-derived sEV-Ps were blocked with 100 μg ml^−1^ human intravenous immune globulin for 4 h under constant rotation at room temperature and washed three times with PBS containing 20 mM HEPES (PBS-H). All washes, unless stated otherwise, were performed in triplicate for 30 min using a S100-AT4 fixed-angle rotor at 228,000*g*. The sEV-Ps were then stained simultaneously with CD81 (0.14 μg ml^−1^; BD) directly conjugated to phycoerythrin and cetuximab directly conjugated to Alexa Fluor-647 (0.25 μg ml^−1^) for 4 h under constant rotation at 4 °C and washed three times with PBS-H. All subsequent staining reactions were performed for 4 h under constant rotation at 4 °C in PBS-H with 100 μg ml^−1^ intravenous immune globulin. To establish an unstained baseline, 100 μg of DiFi cell-derived exosomes were blocked with 100 μg ml^−1^ human intravenous immune globulin as described above, diluted to a final concentration of 1 ng ml^−1^ and FAVS was performed as previously described^61^. All FAVS analyses and sorting were performed on a FACS Aria IIIu flow cytometer customized with a forward scatter photomultiplier tube. The BD FACSDiva 8.1.3. software was used for flow-cytometry data acquisition. The gating strategy is displayed in Extended Data Fig. 7g. Equal number of sorted sEVs were lysed for immunoblotting.

For FAVS staining and analysis of the sEV-Ps, exomeres and supermeres derived from DiFi cells or human plasma, 100 µg sample was blocked and processed as described earlier. For samples that were incubated with directly conjugated primary antibodies, the samples were washed three times in PBS-H and centrifuged at 304,000*g* with a S100-AT4 fixed-angle rotor for 30 min, unless stated otherwise. For samples that were stained with unconjugated primary antibodies, after an overnight incubation at 4 °C, the samples were washed twice, incubated with secondary antibody for 1 h at room temperature and then washed three times in PBS-H for single-colour analysis. For dual colour-stained samples with one directly conjugated and one unconjugated primary antibody, the samples were first stained with unconjugated primary antibody and then washed as described earlier, except that after incubation with the secondary antibody, the samples were washed twice, then the samples were stained with the directly conjugated primary antibody for the second colour and washed three times in PBS-H as described earlier. The samples were then analysed. Nanoparticles incubated with secondary antibody only were used as negative controls. The primary antibodies used as directly conjugated antibodies were: anti-DPEP1 (1:1,000, phycoerythrin-conjugated; LSBio, LS-A109972), anti-FASN (1:250, Alexa Fluor-647-conjugated; Santa Cruz Biotechnology, clone G-11, sc-48357), anti-c-MET (1:400, Alexa Fluor-647-conjugated; R&D, clone 95106, FAB3582R), anti-CD81 (1:300, Alexa Fluor-647-conjugated; R&D, clone 454720, FAB4615P), and anti-EGFR (CTX) (chimaeric mouse/human, 1:400, Alexa Fluor-647-conjugated; purchased from the Vanderbilt-Ingram Cancer Center pharmacy). The unconjugated primary antibodies were: anti-TGFBI (1:350; Proteintech, 10188-1-AP), anti-GPC1 (1:300; Abcam, clone EPR19285, ab199343), anti-CEACAM5/CEA (1:400; Abcam, clone EPCEAR7, ab133633), anti-Ago2 (1:350; Abcam, clone EPR10411, ab186733) and anti-APP (1:350; Millipore, clone 22C11, MAB348). The secondary antibodies used were: goat anti-rabbit (H+L) (Invitrogen, A32733), donkey anti-goat (H+L) (Invitrogen, A32814) and goat anti-mouse (H+L) (Invitrogen, A865).

### RNA purification from cells, sEV-Ps, exomeres and supermeres

RNA was purified using a miRNeasy mini kit (Qiagen, 217004) according to the manufacturer’s protocol. The concentration and integrity of the RNA were estimated using a Quant-It RiboGreen RNA assay kit (Thermo Fisher Scientific) and High sensitivity RNA kit on the 5300 fragment analyzer (Agilent Technologies), respectively.

### Small-RNA library preparation and sequencing

All RNA sequencing was performed at Hudson Alpha. The concentration and integrity of the RNA were estimated using a Quant-It RiboGreen RNA assay kit (Thermo Fisher Scientific) and High sensitivity RNA kit on a 5300 Fragment analyzer (Agilent Technologies), respectively. Total RNA from each sample was taken into a small-RNA library preparation protocol using an Automated NEXTflex small RNA-seq kit v3 (Bioo Scientific, PerkinElmer) for Illumina Libraries on a PerkinElmer Scilone G3 NGS workstation according to the manufacturer’s protocol. The final library concentration and profile were assessed using a Quant-iT Picogreen dsDNA assay kit (Thermo Fisher Scientific) and High sensitivity (HS) DNA assay on the Caliper LabChip Gx (PerkinElmer Inc.), respectively. Quantitative PCR was performed on the final libraries using a KAPA Biosystems library quantification kit to determine the exact nanomolar concentration. Each library was diluted to a final concentration of 1.5 nM and pooled in equimolar ratios. Single-end sequencing (50 bp) was performed on an Illumina NovaSeq 6000 sequencer.

### Small RNA-seq analysis

Cutadapt (https://github.com/marcelm/cutadapt) was used to trim adaptors. TIGER v202001 (https://github.com/shengqh/TIGER) was used to perform small RNA-seq analysis, including read mapping, miRNA quantification and differential analysis. Specifically, Bowtie was used to map reads to the human miRNAs from miRBase v22 and the human reference genome hg19. Data were normalized to the total number of reads in each sample. Principal component analysis was performed to assess the similarity between samples. DESeq2 was used to detect differential expression between cells, the sEV-P, exomeres and supermeres. The miRNAs with a fold change of >2 and FDR < 0.05 were considered to be significantly differentially expressed.

### Quantitative RT-PCR

Analysis of the miRNA levels was performed using TaqMan small RNA assays (Applied Biosystems, cat. no. 4366596) and TaqMan Fast Advanced Master Mix (Applied Biosystems, cat. no. 4444556) according to the manufacturer’s instructions, with U6 small nuclear RNA (U6 snRNA) as the internal control. Briefly, 10 ng of total RNA was used per reverse transcription reaction (15 µl total per reaction); 0.5 μl of the resultant complementary DNA was used in 20 μl quantitative PCR reactions. Quantitative real-time PCR was performed on a Bio-Rad CFX96 C1000 Touch Thermal cycler using the iQ SYBR Green supermix (Bio-Rad). Relative measurement of gene expression was calculated following the manufacturer’s instructions using the ΔΔ*C*_t_ method. U6 was used to calculate the normalized fold change. The following reagents were used: hsa-miR-1246 (cat. no. 4427975, assay ID: 462575_mat, Thermo Fisher Scientific), hsa-miR-675 (cat. no. 4427975, assay ID: 002005, Thermo Fisher Scientific) and U6 snRNA (cat. no. 4427975, assay ID: 001973, Thermo Fisher Scientific).

### Fluorescence in situ hybridization for hsa-miR-1246

Paraffin-embedded sections (5 μm) of colonic tissue and TMAs were deparaffinized and rehydrated. In situ hybridization process was performed, and the TSA Plus fluorescence system was used as previously described^62^ as well as the manufacturer’s protocol for the miRCURY LNA microRNA ISH optimization kit (Qiagen). Briefly, the slides were incubated with proteinase K (15 μg ml^−1^) at 37 °C for 10 min and washed three times with PBS. The slides were incubated with peroxidase block (Vector Laboratories, SP-6000) at room temperature for 10 min to block endogenous peroxidase activity. After in situ hybridization for 1 h at 55 °C with locked nucleic acid probes (0.4 nM for hsa-miR-1246, 1 nM of U6 snRNA and 40 nM of Scramble-miR probe), the slides were washed and blocked in blocking solution (2% sheep serum, 1% BSA and 0.1% Tween in PBS) at room temperature for 15 min and incubated with anti-digoxigenin-POD antibody (1:400; Roche, 11207733910) in antibody dilutant solution (1% sheep serum, 1% BSA, PBS and 0.05% Tween) at room temperature for 1 h. To detect digoxigenin, the TSA Plus Cy5 substrate (1:200; PerkinElmer, NEL745001KT) was applied to the slides and incubated at room temperature for 10 min. After washing three times in PBS, the slides were incubated with DAPI for 5 min and mounted with ProLong gold antifade reagent (Invitrogen, P36934). The slides were scanned by the Vanderbilt University Digital Histology Shared Resource Core. The Lan miRNA detection probes consisted of hsa-miR-1246 (Qiagen, cat. no. 33911 YD00610948-BCG), probe sequence 5′–3′ /5DIGN/CCTGCTCCAAAAATCCATT/3DIG_N/; U6 snRNA (Qiagen, YD00699002) and Scramble-miR probe (Qiagen, YD00699004). The experiments on paraffin-embedded colonic tissues and TMAs were approved by umbrella spore IRB no. 070166.

### Treatment of recipient cells with sEV-Ps, exomeres and supermeres in 3D culture

CC or DiFi cells (2,000) were incubated at 37 °C for 30 min with the indicated concentrations of sEV-Ps, exomeres or supermeres derived from CC, SC, CC-CR or DiFi cells. Fresh medium was added with or without cetuximab (0.3 μg ml^−1^) and/or the indicated concentrations of extracellular nanoparticles every 3–4 d. Colonies were observed and counted after 14–17 d using a GelCount system (Oxford Optronix) with identical acquisition and analysis settings, and are represented as the mean ± s.e.m. from triplicate experiments. The images of the colonies were taken using an EVOS fluorescence microscope (Thermo Fisher).

### Lactate-release measurement

Lactate release into the medium was measured using a Glycolysis cell-based assay kit (Cayman Chemical, cat. no. 600450) according to the manufacturer’s instructions. CC cells (2,000) were cultured in type-1 collagen in a 12-well plate and treated with or without the indicated quantities of extracellular nanoparticles for 14 d as described earlier. The medium was collected and used for the assay.

### Immunohistochemistry

The experiments on paraffin-embedded colonic tissues and TMAs were approved by umbrella spore IRB no. 070166. Tumour xenografts were fixed in neutralized formalin and embedded in paraffin. Slices were deparaffinized with serial histoclear and ethanol. Antigen retrieval was performed in citrate buffer (pH 6.0) with high pressure at 110 °C for 15 min and then quenched in 0.03% H_2_0_2_ with sodium azide for 5 min. The slides were incubated with primary antibodies at room temperature for 60 min and then incubated in Dako Envision + system horseradish peroxidase-labelled polymer at room temperature for 30 min. Signal was detected by incubating in a DAB+ substrate chromogen system at room temperature for 5 min. The primary antibodies used were: anti-DPEP1 (1:1,000; Sigma-Aldrich, HPA012783), anti-CD73 (clone D7F9A, 1:300; Cell Signaling Technology, 13160), anti-TGFBI (clone EPR12078(B), 1:300; Abcam, ab170874), anti-FASN (clone G-11, 1:500; Santa Cruz Biotechnology, sc-48357) and anti-AGO2 (clone EPR10411, 1:500; Abcam, ab57113).

### Labelling and uptake of sEV-Ps, exomeres and supermeres in vitro

The sEV-P and extracellular nanoparticles derived from DiFi cells were labelled with Alexa Fluor-647 (Invitrogen, A20173) according to the manufacturer’s instructions. We experimentally determined that potential Alexa Fluor-647 unbound dye aggregates did not remain after the centrifugation and washing protocols used to purify labelled supermeres. To monitor the uptake of the sEV-P, exomeres and supermeres over time, MDA-MB-231 cells (20,000 cells per well) were seeded on a 35-mm dish (P35G-0.170-14-C, MatTek Corporation) in DMEM culture medium overnight. The cells were then treated with either dimethylsulfoxide control or the Alexa Fluor-647-labelled sEV-Ps, exomeres and supermeres (40 µg ml^−1^) in serum-free DMEM media. Images were acquired using a ×60 objective on a VisiTech iSIM with a Nikon Ti base. Fluorescence (640 far red, 10% laser power, 100 ms exposure time) images were taken of three fields of view, each with several cells. Three *z*-slices, 1 µm apart, were taken of each fluorescent field and the maximum *z*-projection was analysed. The cells were imaged every 15 min for 24 h. The average intensity of the far-red channel was measured for each field of view. Each field of view for each treatment (dimethylsulfoxide, sEV-P, exomere and supermere) was averaged and normalized to the starting value (*n* = 1). The images shown are of one representative cell.

For the imaging of cells treated with LysoTracker, MDA-MB-231 cells were treated with labelled supermeres (40 µg ml^−1^) for 24 h as described earlier. LysoTracker red DND-99 (100 nm; Molecular Probes, L7528) was then applied to the cells for 1 h. Images were acquired using an iSIM system.

For inhibitor treatment before supermere uptake, MDA-MB-231 (20,000 cells per well) or HeLa (25,000 cells per well) cells were seeded on a 35 mm dish (MatTek Corporation, P35G-0.170-14-C) in DMEM culture medium for 24 h. The cells were then pre-incubated with the inhibitors in serum-free DMEM medium for 30 min. The following inhibitors were used: 100 nM bafilomycin A (Sigma-Aldrich, SML1661), 20 µM dynasore (Sigma-Aldrich, D7693), 25 µM CK666 (Sigma-Aldrich, SML0006) and 5 µM cytochalasin D (Sigma-Aldrich, C2618). The labelled supermeres (40 µg ml^−1^) were added to the cells for 24 h in the presence of the indicated inhibitors. Images were acquired using a ×60 objective on a VisiTech iSIM with a Nikon Ti base. Bright-field (30 ms exposure time) and fluorescence (640 far red, 10% laser power, 100 ms exposure time) images were taken of ten or more fields of view, each with several cells per field. Three *z*-slices, 1 µm apart, were taken of each fluorescent field and the brightest slice was analysed.

Bright-field images were used to identify cell boundaries and a region of interest (ROI) was manually drawn around each cell in each field of view. These ROIs were then opened on the fluorescent image and the mean fluorescence intensity of each ROI (cell) was measured. For each field of view, a background ROI was drawn in a region with no cells and this background value was subtracted from each cell fluorescence mean in the field of view. Images shown in the figure are representative of the average fluorescence intensity. Dark shadows in the lower right-hand corner of bright-field images represent a bypass filter physically impeding the image and not any data or cell information.

### Animal studies

Male C57BL/6 mice (6–10 weeks old) were purchased from Jackson Laboratories. The mice were tail-vein injected with exomeres or supermeres (100 or 300 µg in 100 µl PBS pH 7.4) derived from DiFi cells. The control group received vehicle (PBS) only. The mice received daily injections for three consecutive days and were killed 24 h after the last injection. All animal studies and procedures were approved by the Vanderbilt University Medical Center Institutional Animal Care and Use Committee (IACUC; protocol no. M2000054, for tail-vein injection).

### Biodistribution of extracellular samples in vivo

The sEV-Ps and extracellular nanoparticles derived from DiFi cells were labelled with IRDye 800 CW NHS ester (LI-COR, 929-70020) according to the manufacturer’s protocol. The labelled sEV-P was pelleted by centrifugation for 40 min at 304,000*g* in a S100-AT4 fixed-angle rotor. The labelled exomeres were pelleted by centrifugation at 167,000*g* in a SW32 Ti swinging-bucket rotor for 16 h. The supermeres were pelleted by centrifugation at 367,000*g* using a Beckman Coulter SW55 Ti rotor for 16 h. The samples were resuspended and washed in PBS (pH 7.4) and then pelleted again as described earlier. We experimentally determined that potential IRDye 800 CW unbound dye aggregates did not remain after the centrifugation and washing protocols used to purify labelled supermeres. Labelled sample (200 µg in 500 µl PBS) was injected intraperitoneally into ten-week-old male C57BL/6 mice. Their organs were harvested 22 h after injection and imaged using the Odyssey imaging system (LI-COR Biosciences). One of the supermere-treated mice died during the experiment; it was thus excluded from the analysis. All animal studies and procedures were approved by the Vanderbilt University Medical Center Institutional IACUC (protocol no. M2100029-00, for intraperitoneal injection).

### Histochemistry

We stained FFPE sections (4 µm) with Gill 2 haematoxylin (Richard-Allan Scientific, 72504) and eosin (Sigma-Aldrich, HT110316). The percentage of surface area composed of large hepatocytes with increased cytoplasmic vacuolations was estimated for each slide by a liver pathologist (V.Q.T.). Freshly frozen optimal cutting temperature compound (Fisher Health Care, 4585)-embedded liver sections (8 µm) were stained with Oil red O (Sigma-Aldrich, 0625). Briefly, the liver sections were fixed in 10% neutral buffered formalin for 10 min, washed with double-distilled water and equilibrated with 60% isopropanol. The Oil red O was dissolved in isopropanol (0.5% wt/vol), filtered (0.22 µm) and diluted with distilled water (3:2) immediately before staining. The liver sections were stained for 15 min at room temperature, washed with 60% isopropanol, counterstained with Gill 2 haematoxylin and mounted with Vectamount (Vector Laboratories, H-5501). The stained sections were scanned using an Aperio Versa 200 system (Leica Microsystems GmbH) in the Digital Histology Shared Resource at Vanderbilt University Medical Center. Positive surface area was automatically assessed using Tissue IA v2.0 integrated into the Leica Digital Image Hub slide manager platform (Leica Biosystems). Oil red O staining was scored independently by two liver pathologists (V.Q.T. and W.J.H.) for lipid vesicles in a four-tier scheme as follows: 0, no vesicles; 1, rare inconspicuous vesicles in the centrilobular vein (CV) area; 2, conspicuous vesicles present in the CV area; 3, confluent vesicles in the CV area; and 4, confluent vesicles in the CV area, extending between separate CVs.

To highlight polysaccharides such as glycogen, FFPE sections (4 µm) were stained with periodic acid–Schiff with and without diastase. Briefly, the FFPE sections were dewaxed and dehydrated, oxidized for 10 min with periodic acid (Acros Organics, 19840–0050), washed in lukewarm distilled water for 5 min, stained with Schiff reagent (Acros Organics, 61117-5000) for 10 min, washed in lukewarm water for 5 min, counterstained in Gill 2 haematoxylin (Richard-Allan Scientific, 72504) for 4 min, dehydrated and mounted with Acrytol (Electron Microscopy Sciences, 13518). All periodic acid–Schiff-only slides were scored double-blinded and independently by two liver pathologists (V.Q.T. and W.J.H.) for the presence of dark magenta deposits suggestive of glycogen deposition in a three-tier scheme based on the percentage of hepatocytes with dense deposits as follows: 1, 0–33%; 2, 34–66%; and 3, 67–100%. The diastase-treated slides were treated for 20 min with α-amylase from porcine pancreas Type VIB (0.5% in double-distilled water; Sigma-Aldrich, A1376-5000KU) before the periodic acid-staining step to confirm that the dark magenta deposits were polymeric carbohydrates such as hepatic glycogen. Statistics were performed in R with the Wilcoxon rank-sum test for two-group analyses and Kruskal–Wallis one-way ANOVA for more than two groups.

### Liver triglyceride analysis

Snap-frozen liver tissues (50 mg) were homogenized with ceramic beads using a PowerLyzer (Qiagen). The triglyceride content in the liver was quantified using a triglyceride assay kit (Abcam, ab65336) as per the manufacturer’s instructions. The samples were measured on a microplate reader at an optical density of 570 nm.

### RNA isolation from liver tissue

Liver tissue samples were immediately stored in RNAlater (Ambion) until homogenization with ceramic beads using the PowerLyzer (Qiagen) and RNA was extracted using an RNeasy kit (Qiagen) according to the manufacturer’s instructions.

### RNA-seq library preparation for liver-derived RNA

The RNA-seq libraries were prepared using 300 ng RNA and a NEBNext ultra II directional RNA library prep kit (NEB, E7760L). Fragmentation, cDNA synthesis, end repair/dA-tailing, adaptor ligation and PCR enrichment were performed as per the manufacturer’s instructions. Individual libraries were assessed for quality using an Agilent 2100 Bioanalyzer and quantified with a Qubit Fluorometer. The adaptor-ligated material was evaluated using quantitative PCR before normalization and pooling for sequencing. The libraries were sequenced using a NovaSeq 6000 system with 150 bp paired-end reads. RTA (version 2.4.11; Illumina) was used for base calling and quality control of the data was completed using MultiQC v1.7 by the Vanderbilt Technologies for Advanced Genomics (VANTAGE) core (Vanderbilt University).

### RNA-seq analysis of liver-derived RNA

Adaptors were trimmed using Cutadapt (https://github.com/marcelm/cutadapt). After trimming, the RNA-seq reads were mapped to the mouse genome mm10 using STAR and quantified by featureCounts. DESeq2 was used to detect differential expression between supermere- or exomere-treated samples and PBS. Genes with a fold change of >1.5 and FDR < 0.1 were considered to be significantly differentially expressed. GSEA was used to perform functional enrichment analysis against Hallmark gene sets from MSigDB.

### Statistics and reproducibility

All experiments were independently repeated at least twice with similar results, unless otherwise indicated in the figure legends. No statistical method was used to predetermine the sample size. No data were excluded from the analyses. For in vivo experiments, the mice were randomly assigned to different treatment groups. For mouse liver-tissue staining, blinded evaluation was done by two pathologists. Statistical analyses were performed using the SPSS Statistical Analysis System (version 22.0), R (The R foundation) and GraphPad Prism for Windows (version 9.0). Data were presented as the mean ± s.e.m. All statistical tests were two-sided and a *P* value of less than 0.05 was considered statistically significant, with the exception of AFM imaging analysis where a *P* value of less than 0.01 was considered statistically significant. The statistical tests used are indicated in the figure legends. Adjustment for multiple comparisons of significance between groups was performed using the Holm–Bonferroni procedure for ANOVA or Dunn’s multiple comparison test for Kruskal–Wallis, as indicated in the corresponding figure legends. The statistical analyses for drug resistance, lactate release, RT-PCR and TGFBI ELISA assays were all performed using two-sided Student’s *t*-tests; no adjustments were made for multiple comparisons. Differentially expressed miRNAs in sEV-Ps, exomeres and supermeres derived from DiFi cells and differentially expressed genes in the mouse liver were generated by Deseq2 (two-sided). *P* values were adjusted for multiple comparisons using the Benjamini–Hochberg correction. Enriched pathways were generated by GSEA and *P* values were adjusted for multiple comparisons using the Benjamini–Hochberg correction. The Kaplan–Meier method was used for the analysis of overall and progression-free survival of patients with CRC comparing the DPEP1-staining pattern (diffuse versus others), and data were compared between marker groups using a two-sided log-rank test. TGFBI staining and statistical analysis were performed as described for DPEP1. For the immunoblotting data, each blot was repeated at least twice with similar results and a representative blot is displayed. For the in vitro particle uptake data presented in Fig. 1d,f, the experiments were repeated twice independently.

### Reporting Summary

Further information on research design is available in the Nature Research Reporting Summary linked to this article.

## Online content

Any methods, additional references, Nature Research reporting summaries, source data, extended data, supplementary information, acknowledgements, peer review information; details of author contributions and competing interests; and statements of data and code availability are available at 10.1038/s41556-021-00805-8.

## Supplementary information

Reporting Summary

Supplementary Tables 1–10. Supplementary Table 1. Complete list of proteins in sEVs, NV, exomeres and supermeres derived from DiFi cells identified by LC–MS/MS. Proteins with an average count ≥ 1 in each fraction were considered detectable. Spectral counts of proteins were normalized to the total spectral counts and log_2_-transformed. NV, non-vesicular; and sEV, small extracellular vesicle. Supplementary Table 2. Complete list of proteins in supermeres derived from DiFi, PANC-1 and MDA-MB-231 cells identified by LC–MS/MS. Proteins with an average count ≥ 1 in each fraction were considered detectable. Spectral counts of proteins were normalized to the total spectral counts and log_2_-transformed. NV, non-vesicular; and sEV, small extracellular vesicle. Supplementary Table 3. Top-20 most abundant proteins in supermeres derived from DiFi, PANC-1 and MDA-MB-231 cells identified by LC–MS/MS. Proteins with an average count ≥ 1 in each fraction were considered detectable. Spectral counts of proteins were normalized to the total spectral counts and log_2_-transformed. NV, non-vesicular; and sEV, small extracellular vesicle. Supplementary Table 4. Differential expression of miRNAs between cells and the sEV-P, exomeres and supermeres derived from DiFi cells. List of miRNAs generated by RNA-seq. sEV-P, small extracellular vesicle pellet. The *P* values were adjusted for multiple comparisons using the Benjamini–Hochberg procedure to decrease the FDR. Supplementary Table 5. Mean *C*_t_ value for miR-1246 and U6 in DiFi cells and extracellular compartments by qRT-PCR analysis. sEV-P, small extracellular vesicle pellet; Exom, Exomere; and Super, Supermere. Supplementary Table 6. Top-ten small nuclear RNAs in supermeres, apart from RNU2-1, that contain the miR-1246 sequence. The top-ten snRNAs in supermeres, apart from RNU2-1, by average reads. The red box marks the miR-1246 sequence. By average reads supermeres have an 8–19-, 4–6-, and 5–161-fold increase in read number between supermeres and small EV pellet, exomeres and cells, respectively. To the right of the alignment is the fold increase of supermeres over small EV pellet (S/P), exomeres (S/X) and cells (S/C). The numbers in red are from snRNAs that diverge from the miR-1246 sequence. The aligned region around the miR-1246 region is shown. Supplementary Table 7. Total normalized small-RNA reads containing miR-1246 in DiFi cells and extracellular fractions. sEV-P, small extracellular vesicle pellet; Exom, Exomere; and Super, Supermere. Supplementary Table 8. Expression of RNA-binding proteins in sEVs, NV, exomeres and supermeres. List of published RNA-binding proteins^63,64^ present in sEVs, NVs, exomeres and supermeres derived from DiFi cells identified by LC–MS/MS. NV, non-vesicular; and sEV, small extracellular vesicle. Supplementary Table 9. RNA-seq of mouse liver cells after treatment with exomeres and supermeres. Gene expression in mouse liver cells (exomere treated versus PBS and supermere treated versus PBS) and GSEA pathway analysis. The *P* values were adjusted for multiple comparisons using the Benjamini–Hochberg procedure to generate FDR-adjusted *q*-values. Supplementary Table 10. Select GSEA pathways significantly down- or upregulated in mouse liver cells compared with PBS following treatment with exomeres or supermeres. Based on data from Supplementary Table 9.

## Data Availability

The mass spectrometry proteomic data have been deposited to the ProteomeXchange Consortium via the PRIDE partner repository with the dataset identifiers PXD025213 and PXD027258. The RNA-seq data that support the findings of this study have been deposited with NCBI (accession number GSE168418). Previously published RNA sequencing data (DKO-1 and Gli36 miRNA datasets) that were re-analysed here are available under the accession code GSE125905 (ref. ^1^). The microarray platform U133 plus 2.0 can be found at http://gent2.appex.kr/gent2/. The Cancer Genome Atlas RNA-seq can be found at http://firebrowse.org/viewGene.html. All other data supporting the findings of this study are available from the corresponding author on reasonable request. Source data are provided with this paper.

## Source data

Source Data Fig. 1 Statistical source data.
Source Data Fig. 1 Unprocessed western blots.
Source Data Fig. 2 Statistical source data.
Source Data Fig. 2 Unprocessed western blots.
Source Data Fig. 3 Statistical source data.
Source Data Fig. 3 Unprocessed western blots.
Source Data Fig. 4 Unprocessed western blots.
Source Data Fig. 5 Statistical source data.
Source Data Fig. 5 Unprocessed western blots.
Source Data Fig. 6 Statistical source data.
Source Data Fig. 6 Unprocessed western blots.
Source Data Fig. 7 Unprocessed western blots.
Source Data Extended Data Fig. 1 Unprocessed western blots.
Source Data Extended Data Fig. 2 Statistical source data.
Source Data Extended Data Fig. 2 Unprocessed western blots.
Source Data Extended Data Fig. 3 Statistical source data.
Source Data Extended Data Fig. 4 Unprocessed western blots.
Source Data Extended Data Fig. 5 Statistical source data.
Source Data Extended Data Fig. 5 Unprocessed western blots.
Source Data Extended Data Fig. 6 Statistical source data.
Source Data Extended Data Fig. 7 Unprocessed western blots.
