# Supplementary material for: Supermeres are functional extracellular nanoparticles replete with disease biomarkers and therapeutic targets
Source: Nat Cell Biol. 2021 Dec 9;23(12):1240–54. doi: 10.1038/s41556-021-00805-8 (PMC8656144; doi:10.1038/s41556-021-00805-8)
Supplement: Supplementary file 1 — Reporting Summary [file 41556_2021_805_MOESM1_ESM.pdf]

nature research

Corresponding author(s): Robert J. Coffey

Last updated by author(s): Oct.20, 2021

Reporting Summary

Nature Research wishes to improve the reproducibility of the work that we publish. This form provides structure for consistency and transparency in reporting. For further information on Nature Research policies, see our Editorial Policies and the Editorial Policy Checklist.

Statistics

For all statistical analyses, confirm that the following items are present in the figure legend, table legend, main text, or Methods section.

n/a	Confirmed	
☐	☒	The exact sample size (*n*) for each experimental group/condition, given as a discrete number and unit of measurement
☐	☒	A statement on whether measurements were taken from distinct samples or whether the same sample was measured repeatedly
☐	☒	The statistical test(s) used AND whether they are one- or two-sided<br>*Only common tests should be described solely by name; describe more complex techniques in the Methods section.*
☒	☐	A description of all covariates tested
☐	☒	A description of any assumptions or corrections, such as tests of normality and adjustment for multiple comparisons
☐	☒	A full description of the statistical parameters including central tendency (e.g. means) or other basic estimates (e.g. regression coefficient) AND variation (e.g. standard deviation) or associated estimates of uncertainty (e.g. confidence intervals)
☒	☐	For null hypothesis testing, the test statistic (e.g. *F*, *t*, *r*) with confidence intervals, effect sizes, degrees of freedom and *P* value noted<br>*Give P values as exact values whenever suitable.*
☒	☐	For Bayesian analysis, information on the choice of priors and Markov chain Monte Carlo settings
☒	☐	For hierarchical and complex designs, identification of the appropriate level for tests and full reporting of outcomes
☒	☐	Estimates of effect sizes (e.g. Cohen's *d*, Pearson's *r*), indicating how they were calculated

Our web collection on statistics for biologists contains articles on many of the points above.

Software and code

Policy information about availability of computer code

Data collection	BD FACSDiva 8.1.3. software was used for flow cytometry data acquisition; Gwyddion was used for exporting and processing AFM Images; SIM images were analysed using ImageJ software.
Data analysis	TIGER v202001(https://github.com/shengqh/TIGER), was used to perform small RNA-seq analysis. RNAseq reads were mapped to the mouse genome mm10 using STAR (v2.7.3a), and quantified by featureCounts (v2.0.0). DESeq2 (v1.24.0) was used to detect differential expression.

For manuscripts utilizing custom algorithms or software that are central to the research but not yet described in published literature, software must be made available to editors and reviewers. We strongly encourage code deposition in a community repository (e.g. GitHub). See the Nature Research guidelines for submitting code & software for further information.

Data

Policy information about availability of data

All manuscripts must include a data availability statement. This statement should provide the following information, where applicable:

- Accession codes, unique identifiers, or web links for publicly available datasets
- A list of figures that have associated raw data
- A description of any restrictions on data availability

The mass spectrometry proteomics data have been deposited to the ProteomeXchange Consortium via the PRIDE partner repository with the dataset identifier PXD025213 and PXD027258.

The RNA-seq data that support the findings of this study have been deposited with NCBI (accession number GSE168418). https://www.ncbi.nlm.nih.gov/geo/query/acc.cgi?acc=GSE168418

Microarray platform U133 plus 2.0: http://gent2.appex.kr/gent2/
TCGA RNAseq: http://firebrowse.org/viewGene.html

The DKO-1 and Gli36 miRNA datasets was from (Jeppesen et al. 2019, https://www.cell.com/cell/article/S0092-8674(19)30212-0/fulltext). All other data supporting the findings of this study are available from the corresponding author upon reasonable request.

Field-specific reporting

Please select the one below that is the best fit for your research. If you are not sure, read the appropriate sections before making your selection.

☒ Life sciences ☐ Behavioural & social sciences ☐ Ecological, evolutionary & environmental sciences

For a reference copy of the document with all sections, see nature.com/documents/nr-reporting-summary-flat.pdf

Life sciences study design

All studies must disclose on these points even when the disclosure is negative.

Sample size	Sample size was chosen based on similar studies performed in our lab and those reported in the literature.
Data exclusions	No data was excluded from the studies.
Replication	Replication was carried out for key in vitro and in vivo experiments as described in the figure legends and materials and methods.
Randomization	For in vitro experiments, randomization was not applicable. For in vivo experiments, mice were randomly assigned to different treatment groups.
Blinding	For mouse liver tissue staining, blinded evaluation was done by two pathologists.

Reporting for specific materials, systems and methods

We require information from authors about some types of materials, experimental systems and methods used in many studies. Here, indicate whether each material, system or method listed is relevant to your study. If you are not sure if a list item applies to your research, read the appropriate section before selecting a response.

Materials & experimental systems

n/a	Involved in the study	
☐	☒	Antibodies
☐	☒	Eukaryotic cell lines
☒	☐	Palaeontology and archaeology
☐	☒	Animals and other organisms
☐	☒	Human research participants
☒	☐	Clinical data
☒	☐	Dual use research of concern

Methods

n/a	Involved in the study	
☒	☐	ChIP-seq
☐	☒	Flow cytometry
☒	☐	MRI-based neuroimaging

Antibodies

Antibodies used

Immunoblot analysis.
The primary antibodies used were: anti-EEF1A1 (clone EPR9471, ab157455), anti-A33 (clone EPR4240, ab108938), anti-EPCAM (clone E144, ab32392), anti-AGO2 (clone EPR10411, ab186733), anti-Syntenin-1 (clone EPR8102, ab133267), anti-ACE2 (clone EPR4435(2), ab108252), anti-APP (clone Y188, ab32136), anti-GPC1 (clone EPR19285, ab199343), anti-CEACAM5/CEA (clone EPCEAR7, ab133633), anti-TPI1 (ab96696), anti-LDHB (clone 60H11, ab85319), anti-GPI (clone 1B7D7, ab66340), anti-HSPA8 (clone EP1531Y, ab51052), anti-PCSK9 (clone EPR7627(2), ab181142), anti-VPS35 (clone EPR11501(B), ab157220) and anti-MVP (clone EPR13227(B), ab175239) are from Abcam.

Anti-MET (clone D1C2, 8198), anti- CEACAM5/CEA (clone CB30, 2383), anti-CD73 (clone D7F9A,13160), anti-FASN (clone C20G5, 3180), anti-ACLY (4332), anti-AGO1 (clone D84G10, 5053), anti-XPO5 (clone D7W6W, 12565), anti-HNRNPA2B1 (clone 2A2, 9304), anti-Alix (clone 3A9, 2171), anti-ALDOA (clone D73H4, 8060), anti-ENO1 (3810), anti-ENO2 (clone D20H2, 8171), anti-HK1 (clone C35C4, 2024), anti-PKM1/2 (clone C103A3, 3190), anti-LDHA (clone C4B5, 3582), anti-pAKT (9271), anti-AKT (9272), anti-pERK1/2 (9101), anti-ERK1/2 (9102) and anti-HSP90 (clone C45G5, 4877) are from Cell Signaling Technology.

Anti-HSPA13 (clone A-11, sc-398297), anti-ACE (clone E-9, sc-271860), anti-FASN (clone G-11, sc-48357), and anti-CD9 (clone C-4, SC-13118) are from Santa Cruz. Anti-APP (clone 22C11, MAB348), anti-β-Actin (clone AC-74, A5316), anti-DPEP1 (HPA012783) and anti-EGFR (06-847) are from Sigma.

Anti-GPC-1 (Invitrogen, PA5-28055), anti-MET (AF276) and anti-CD81 (clone 454720, MAB4615) are from R &D Systems. Anti-AREG

(6R1C2.4) is from Bristol-Myers Squibb Research Institute. Anti-TGFBI (10188-1-AP) is from Proteintech. Anti-FLOT1 (clone 18, 610820), anti-β1-Integrin (clone 18/CD29, 610467) and anti-CD63 (clone H5C6, 556019) are from BD Transduction Laboratories™.

All the antibodies were used at 1:1000 dilution except Synteinin-1 and β-Actin which were 1:5000.

Immunofluorescence staining for confocal microscopy.
The primary antibodies used were: anti-DPEP1 (1:100, Sigma, HPA012783), anti-CD63 (1:100, BD, clone H5C6, 556019), Alexa Fluor® 647 Anti-Sodium Potassium ATPase (Na/KATPase) (1:500, Abcam, clone EP1845Y, ab198367).
Secondary antibody: Donkey anti-Rabbit IgG (1:600, Invitrogen, A21206, Alexa Fluor 488 conjugated)

Fluorescence-activated vesicle sorting (FAVS) staining, sorting and analysis.
Primary antibodies used were: directly conjugated antibodies: anti-DPEP1 (1:1,000, LSBio, LS-A109972, PE-conjugated), anti-FASN (1:250, Santa Cruz, clone G-11, SC-48357, AF-647-conjugated), anti-c-MET (1:400, R&D, clone 95106, FAB3582R, AF-647-conjugated), anti-CD81 (1:300, R&D, clone 454720, FAB4615P, AF-647-conjugated), anti-EGFR (CTX) (chimeric mouse/human, 1:400, purchased from the Vanderbilt-Ingram Cancer Center pharmacy, AF-647-conjugated). Un-conjugated primary antibodies: anti-TGFBI (1:350, Proteintech, 10188-1-AP), anti-GPC1 (1:300, Abcam, clone EPR19285, ab199343), anti-CEACAM5/CEA (1:400, Abcam, clone EPCEAR7, ab133633), anti-Ago2 (1:350, Abcam, clone EPR10411, ab186733), anti-APP (1:350, Millipore, clone 22C11, MAB348).

Secondary antibodies: Goat anti-rabbit (H+L) (1:1,000, Invitrogen A32733, AF647-conjugated), donkey anti-goat (H+L) (1:1,000, Invitrogen, A32814, AF488 conjugated), goat anti-mouse (H+L) (1:1,000, Invitrogen, A865, APC-conjugated).

Immunohistochemistry (IHC).
Primary antibodies used were: anti-DPEP1 (1:1,000, Sigma, HPA012783), anti-CD73 (clone D7F9A, 1:300, Cell Signaling Technology,13160), anti-TGFBI (clone EPR12078(B), 1:300, Abcam, ab170874), anti-FASN (clone G-11, 1:500, Santa Cruz, sc48357), and anti-AGO2 (clone EPR10411, 1:500, Abcam, ab57113).

Validation

All the antibodies are commercially available and have been validated by the manufacturer.

Eukaryotic cell lines

Policy information about cell lines

Cell line source(s)

LS174T, PANC-1, Calu-3, and Hela cell lines were obtained from the American Type Culture Collection (ATCC), Human primary renal proximal tubule epithelial cells (HREC) were from Innovative BioTherapies. LIM1215 cell line was obtained from Ludwig Institute, Melbourne, AU. HCA-7 cell line was obtained from Susan Kirkland (Imperial Cancer Research Fund, London); its derivatives (SC, CC and CC-CR) and DiFi cell lines were developed in Coffy lab. DKO-1 cell line was obtained from Dr. T. Sasazuki at Kyushu University, Gli36 cells were obtained from Dr. X. Breakefield at Harvard Medical School, and MDA-MB-231 and LM2-4175 cells were obtained from Dr. J. Massagué at Memorial Sloan-Kettering Cancer Center.

Authentication

Cell lines were authenticated using short tandem repeat (STR) analysis.

Mycoplasma contamination

All cell lines were tested negative for mycoplasma contamination.

Commonly misidentified lines
(See ICLAC register)

No commonly misidentified cell lines were used.

Animals and other organisms

Policy information about studies involving animals; ARRIVE guidelines recommended for reporting animal research

Laboratory animals

Male C57BL/6 mice (6-10 weeks old) were purchased from Jackson Laboratories.

Wild animals

No wild animals were used in this study.

Field-collected samples

No field collected samples were used in this study.

Ethics oversight

The animal experiments described in this study were carried out with the approval of Vanderbilt University Medical Center Institutional Animal Care and Use Committee (IACUC) with the protocol number M2000054 (for tail vein injection) and M2100029-00 (for Intraperitoneal injection).

Note that full information on the approval of the study protocol must also be provided in the manuscript.

Human research participants

Policy information about studies involving human research participants

Population characteristics

A group of 13 CRC patients ranging in age (30-68 years old) and an average age of 52.2 years old. PID 01-112 DOC 03-13-2019 Age 52 Sex Male, PID 01-113 DOC 03-30-2019 Age 57 Sex Male, PID 01-115 DOC 05-16-2019 Age 65 Sex Female, PID 01-117 DOC 06-18-2019 Age 56 Sex Male, PID 01-120 DOC 10-01-2019 Age 30 Sex Male, PID 01-121 DOC 10-17-2019 Age 46 Sex Male, PID 01-122 DOC 10-30-2019 Age 68 Sex Female, PID 01-123 DOC 3-11-2020 Age 62 Sex Male, PID 01-124 DOC 11-26-2019 Age 63 Sex Male, PID 01-126 DOC 12-09-2019 Age 42 Sex Female, PID 01-128 DOC 02-12-2020 Age 56 Sex Male, PID 01-131 DOC 04-27-2020 Age 43 Sex Male, PID 01-132 DOC 05-25-2021 Age 39 Sex Female.

	Three normal control patients ranging in age (44-71 years old) and an average age of 56 years old. NC 01-001 DOC 07-21-2019 Age 44 Sex Male, NC 01-002 DOC 12-12-2019 Age 53 Sex Male, NC 01-003 DOC 12-12-2019 Age 71 Sex Male.<br><br>Informed consent was obtained by participants. The participant did not receive compensation. There is consent to publish this information.
Recruitment	See details in NCT 03263429
Ethics oversight	The study protocol was approved by the Vanderbilt University Medical Center Institutional Review Board (IRB#161529 and 151721)

Note that full information on the approval of the study protocol must also be provided in the manuscript.

Flow Cytometry

Plots

Confirm that:

☒ The axis labels state the marker and fluorochrome used (e.g. CD4-FITC).

☒ The axis scales are clearly visible. Include numbers along axes only for bottom left plot of group (a 'group' is an analysis of identical markers).

☐ All plots are contour plots with outliers or pseudocolor plots.

☐ A numerical value for number of cells or percentage (with statistics) is provided.

Methodology

Sample preparation	Small EV pellet (sEV-P) derived from DiFi cells were stained and sorted as described in the method. For FAVS staining and analysis of sEV-P, Exomere and Supermere derived from DiFi cells or human plasma, one hundred micrograms of samples were blocked and processed as described in the method. For samples that incubated with directly conjugated primary antibodies, the samples were washed three times and centrifuged at 304,000 × g with a S100-AT4 fixed angle rotor (effective k factor of 29) for 30 min unless stated otherwise. For samples that stained with unconjugated primary antibodies, after incubation for overnight at 4°C, the samples were washed twice, then incubated with secondary antibody for 1 h at RT and then washed three times in PBS-H for single color analysis. For dual-color stained samples with one directly conjugated and one un-conjugated primary antibody, samples were stained with unconjugated primary antibody first, and then washed as described above except that after incubation with the secondary antibody, the samples were washed only twice and then the samples were stained with the directly conjugated primary antibody for the second color and washed three times in PBS-H as described above. The samples are then ready to be analyzed. The nanoparticles incubated with only the secondary antibody were used as negative controls.
Instrument	All FAVS analysis and sorting were performed on a BD FACS ARIA IIIu instrument with FSC-PMT.
Software	All samples were acquired with BD FACSDiva 8.1.3. software.
Cell population abundance	Cells were not used for flow cytometry in this study. Extracellular vesicles and nanoparticles were used.
Gating strategy	sEVs and nanoparticles analysis: greater than 98% of the unstained samples that fell within the lower left (LL) quadrant of a dot-plot (autofluorescence vs the probe emission) were used as negative control (baseline). Stained sEVs and nanoparticles that fell in the lower right (LR) quadrant were considered as epitope positive, while samples falling in the LL quadrant were below the limit of detection.

☒ Tick this box to confirm that a figure exemplifying the gating strategy is provided in the Supplementary Information.
