# Supplementary figures and images for: Supermeres are functional extracellular nanoparticles replete with disease biomarkers and therapeutic targets
Source: Nat Cell Biol. 2021 Dec 9;23(12):1240–54. doi: 10.1038/s41556-021-00805-8 (PMC8656144; doi:10.1038/s41556-021-00805-8)

Fig. 1h

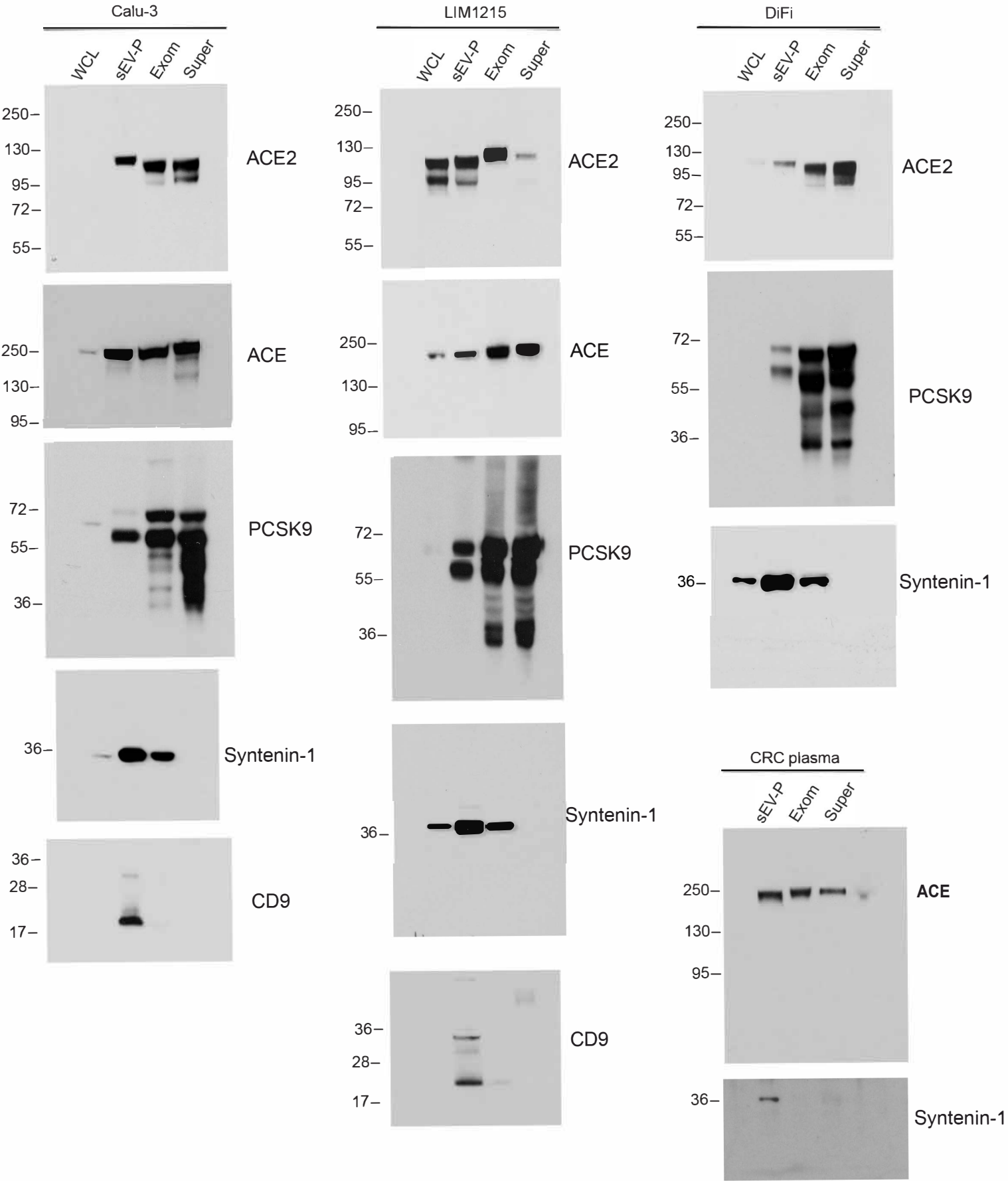

Supplement: Source Data Fig. 1 — Unprocessed western blots. [file 41556_2021_805_MOESM4_ESM.pdf]

**Fig. 2e**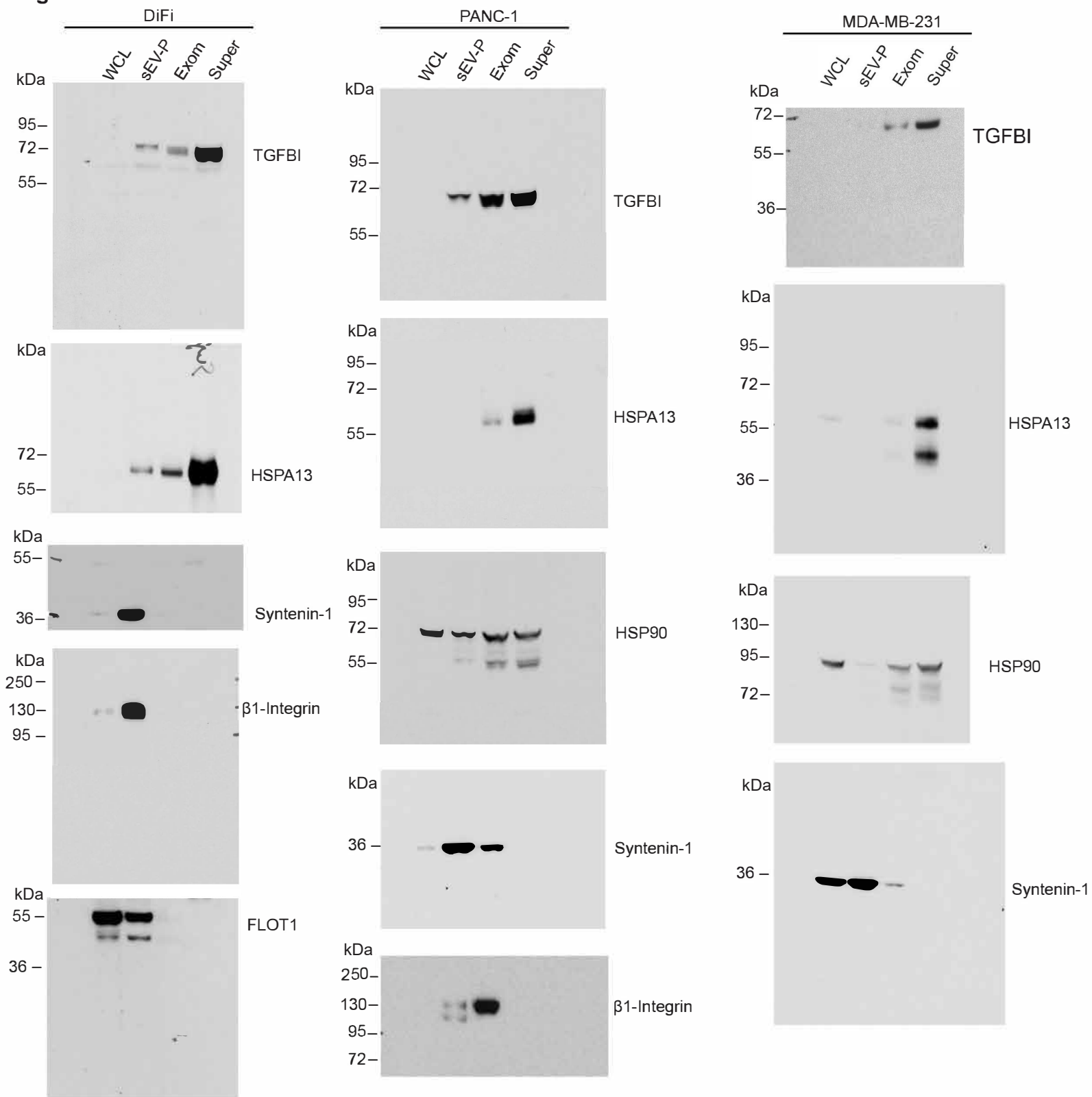

Supplement: Source Data Fig. 2 — Unprocessed western blots. [file 41556_2021_805_MOESM6_ESM.pdf]

Fig. 3c

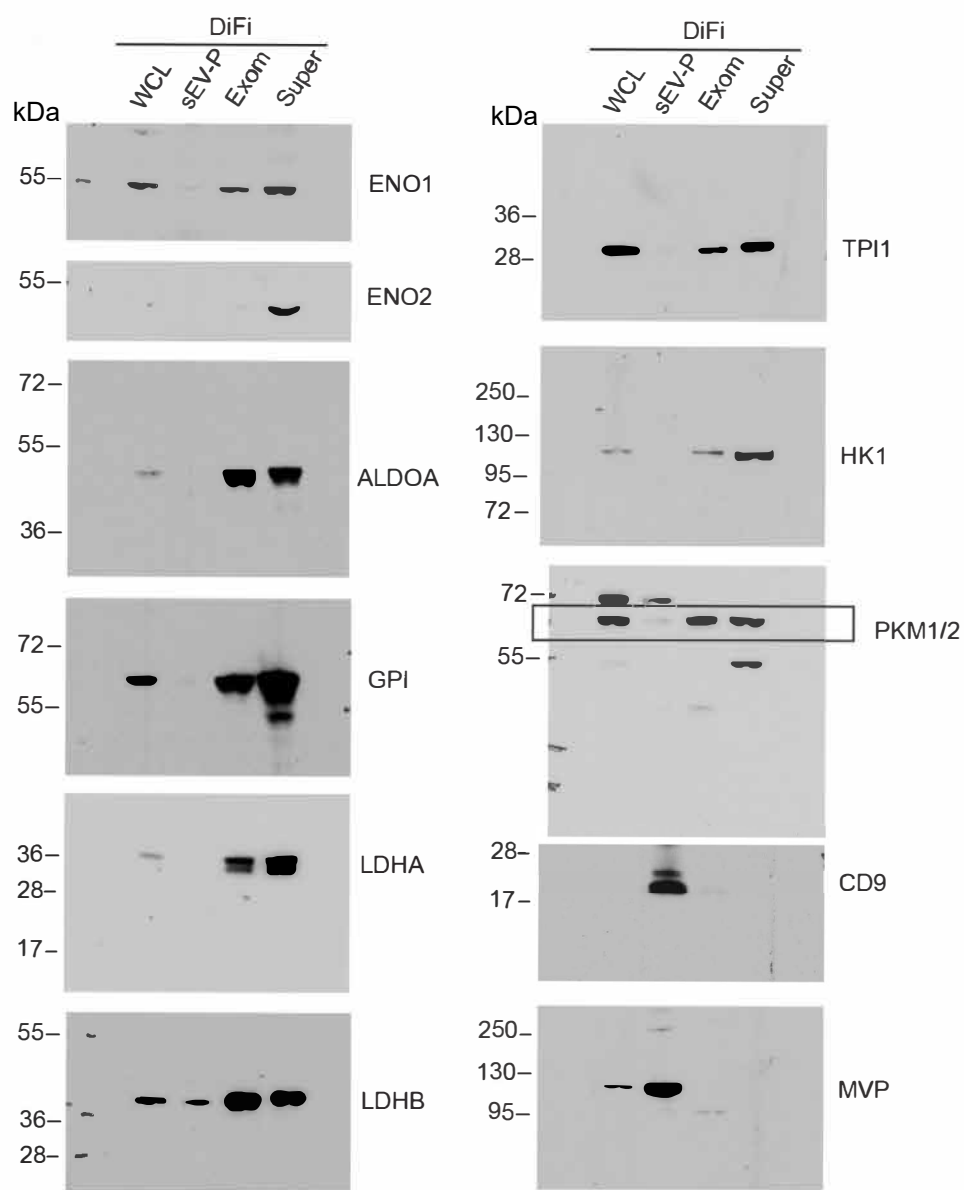

**Fig. 3d**

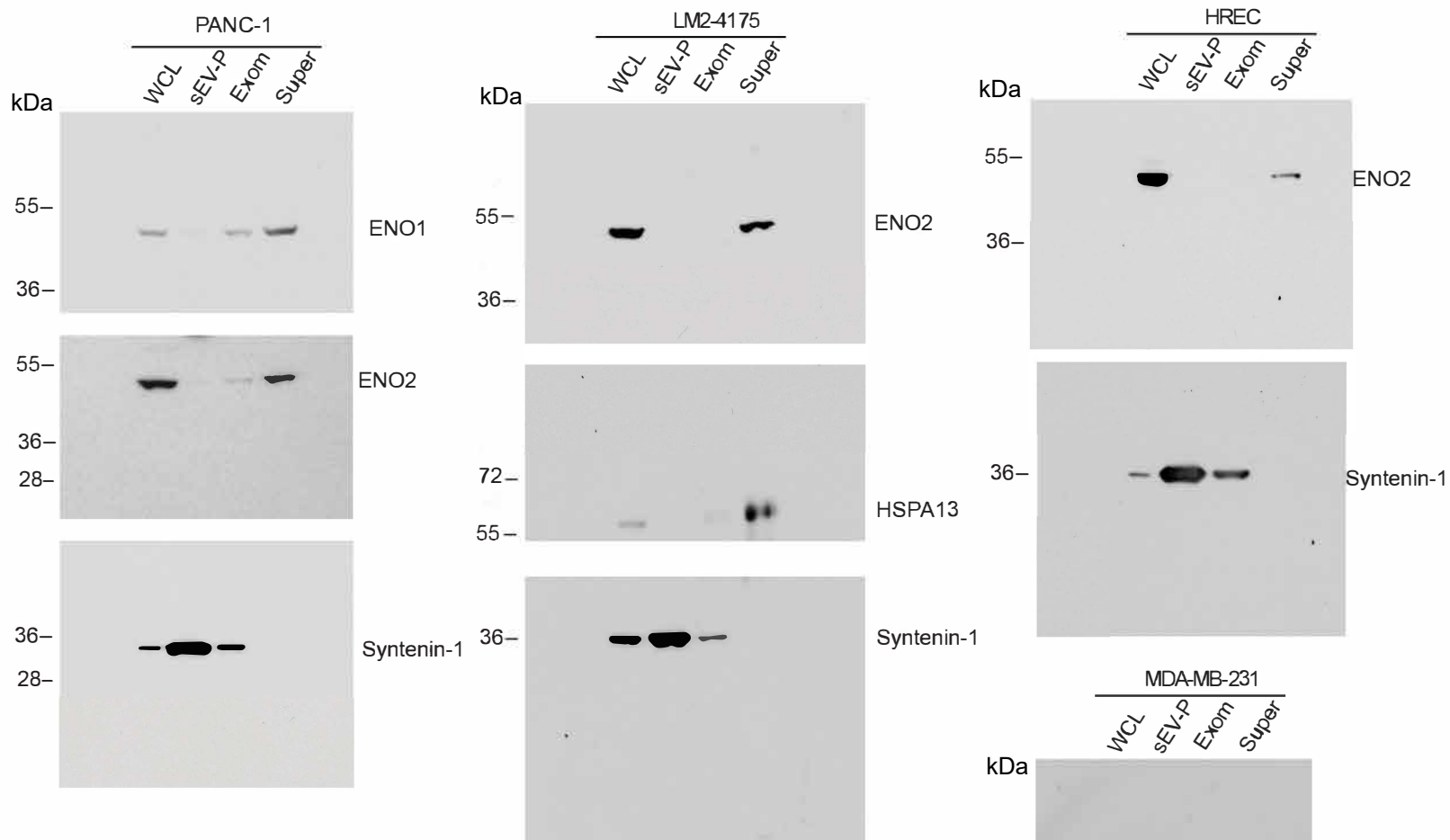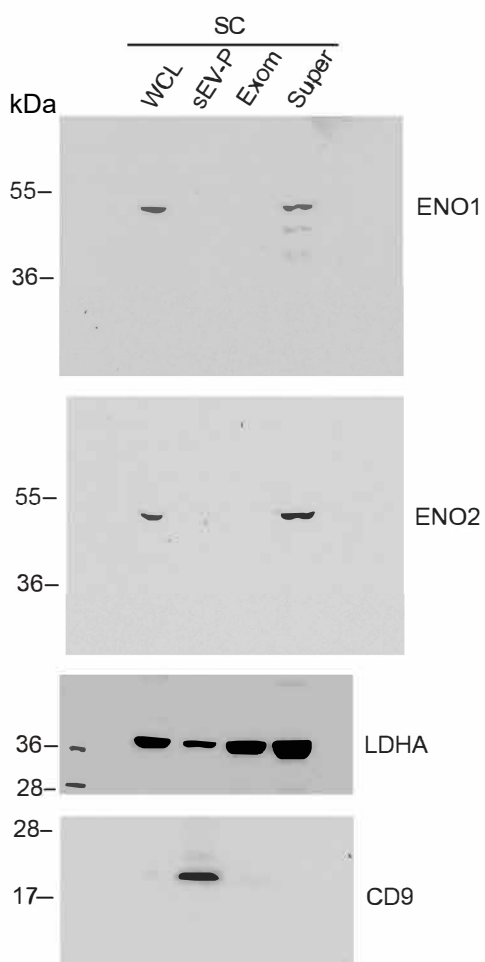

**Fig. 3e**

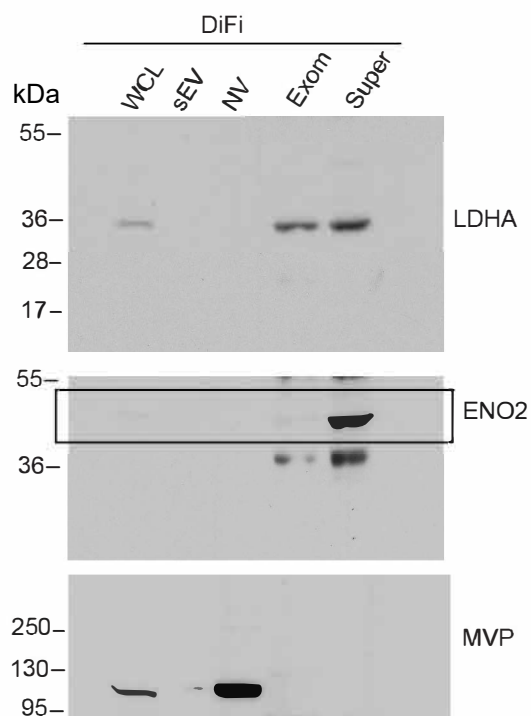

Supplement: Source Data Fig. 3 — Unprocessed western blots. [file 41556_2021_805_MOESM8_ESM.pdf]

**Fig. 4b**

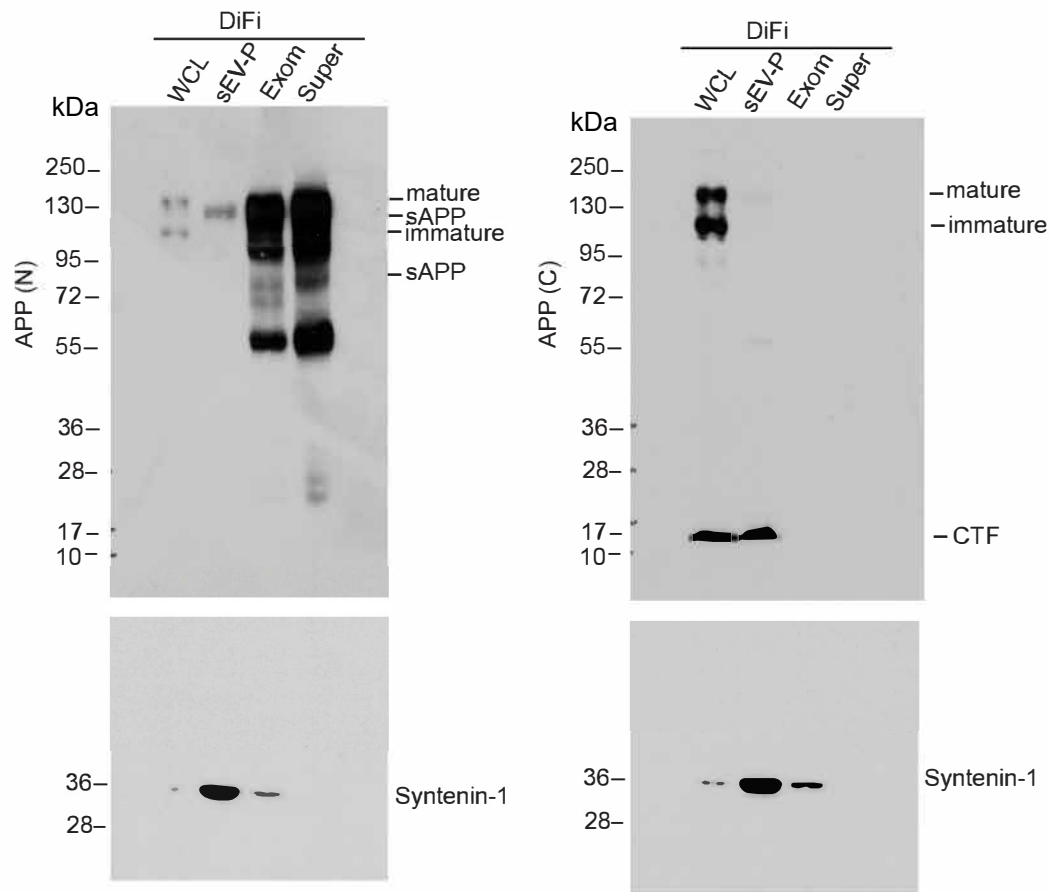

**Fig. 4d**

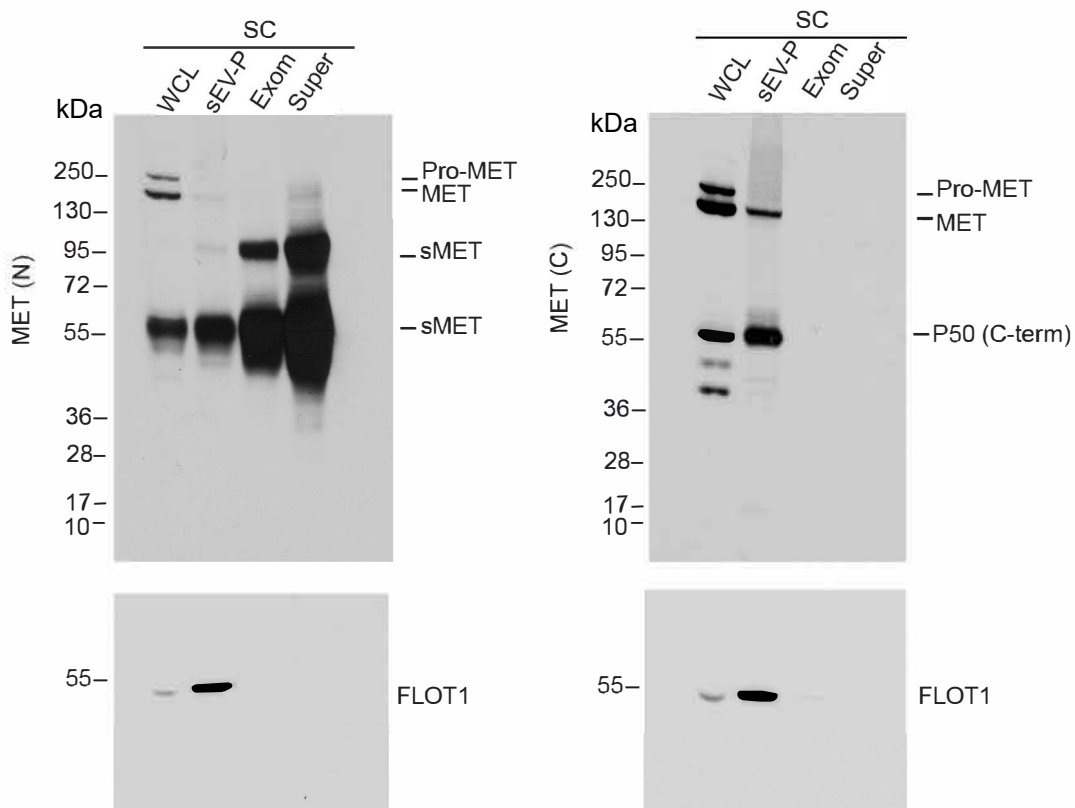

**Fig. 4f**

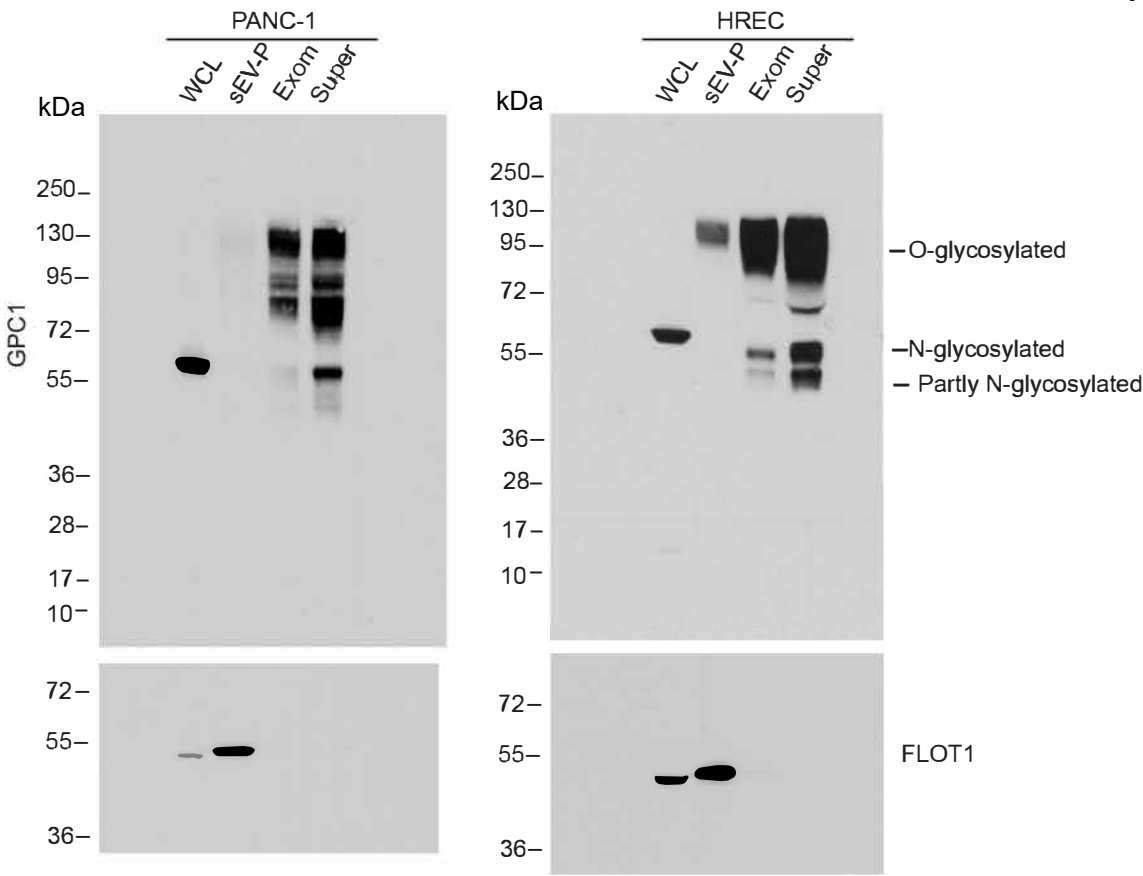

**Fig. 4h**

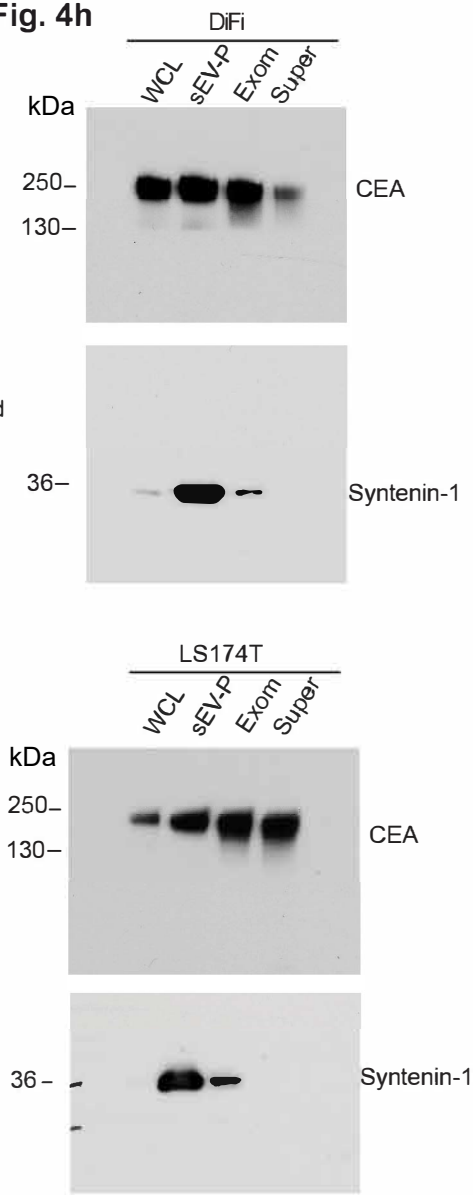

**Fig. 4i**

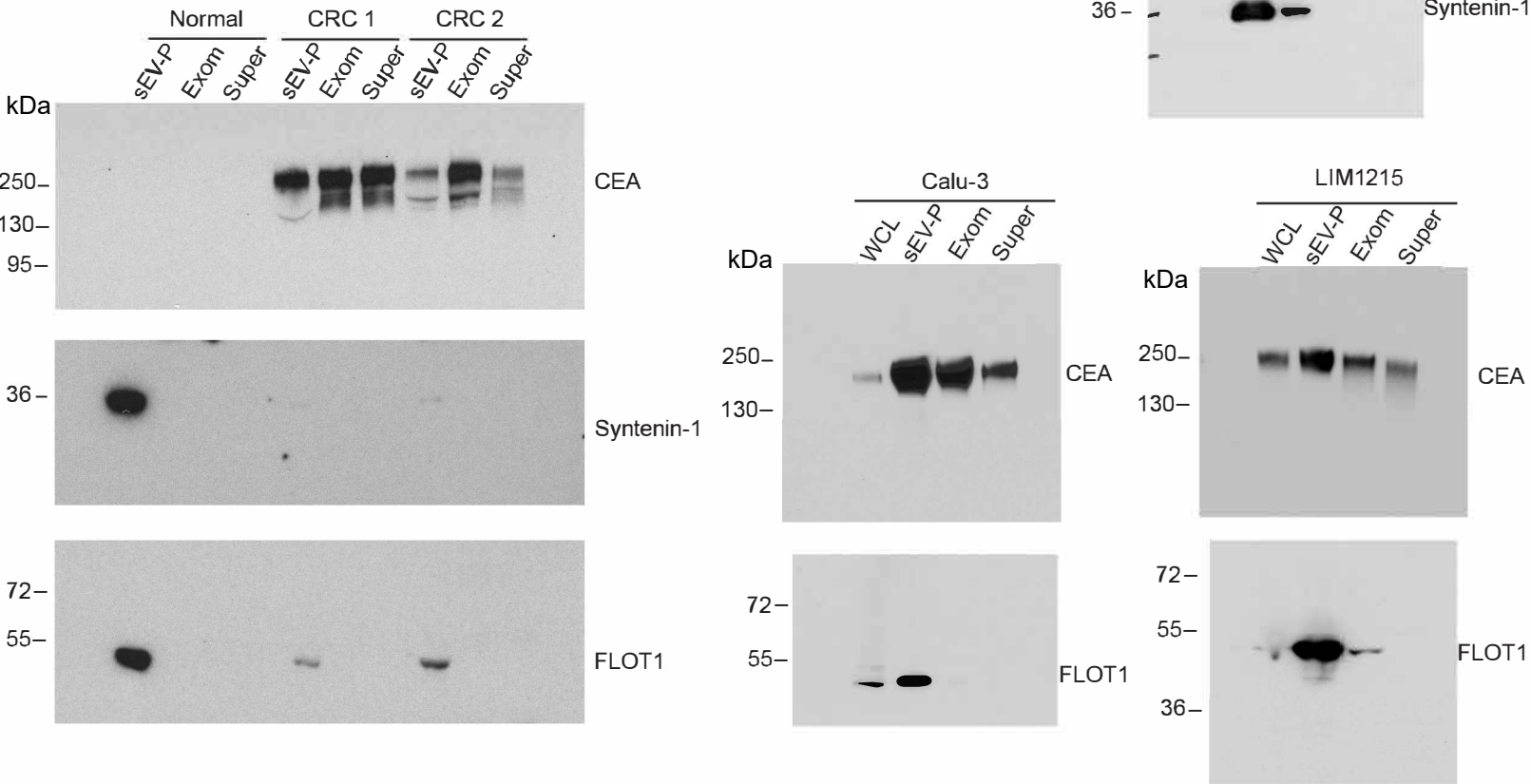

Supplement: Source Data Fig. 4 — Unprocessed western blots. [file 41556_2021_805_MOESM9_ESM.pdf]

**Fig. 5h**

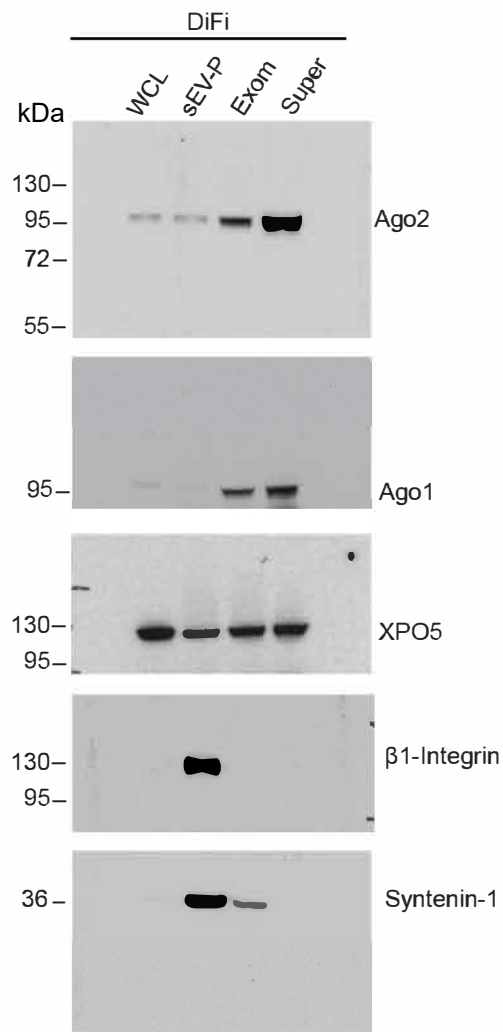

**Fig. 5i**

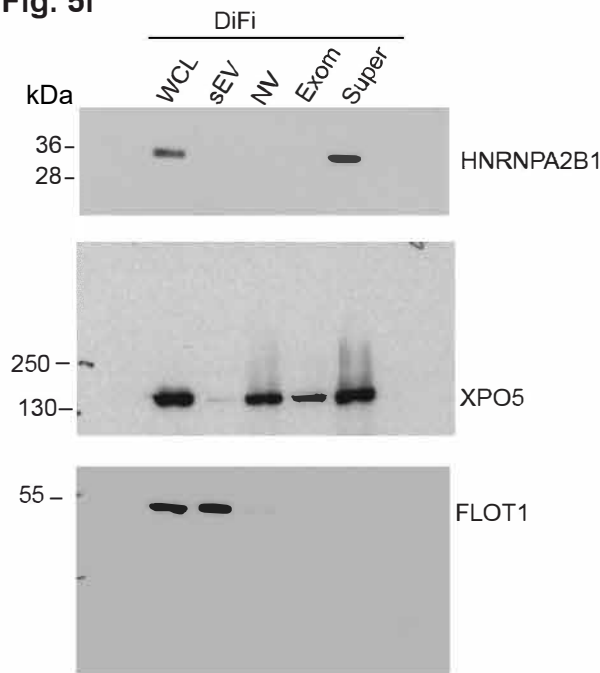

**Fig. 5k**

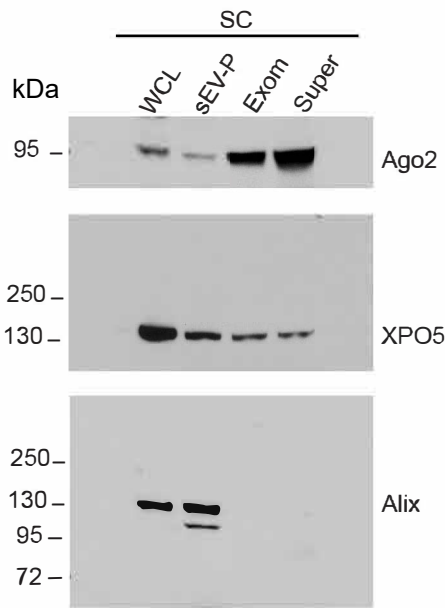

**Fig. 5j**

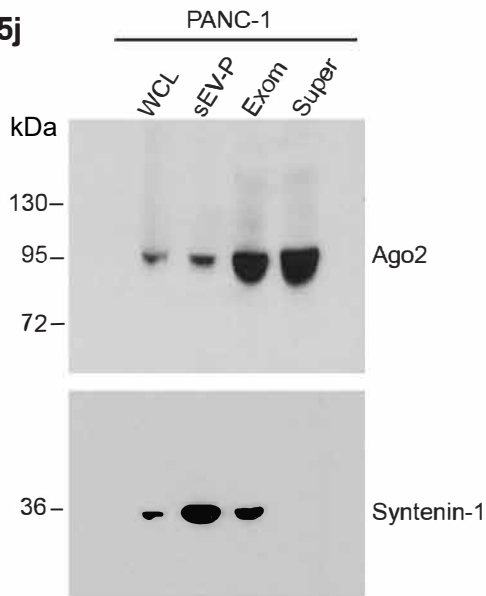

Supplement: Source Data Fig. 5 — Unprocessed western blots. [file 41556_2021_805_MOESM11_ESM.pdf]

Fig. 6g

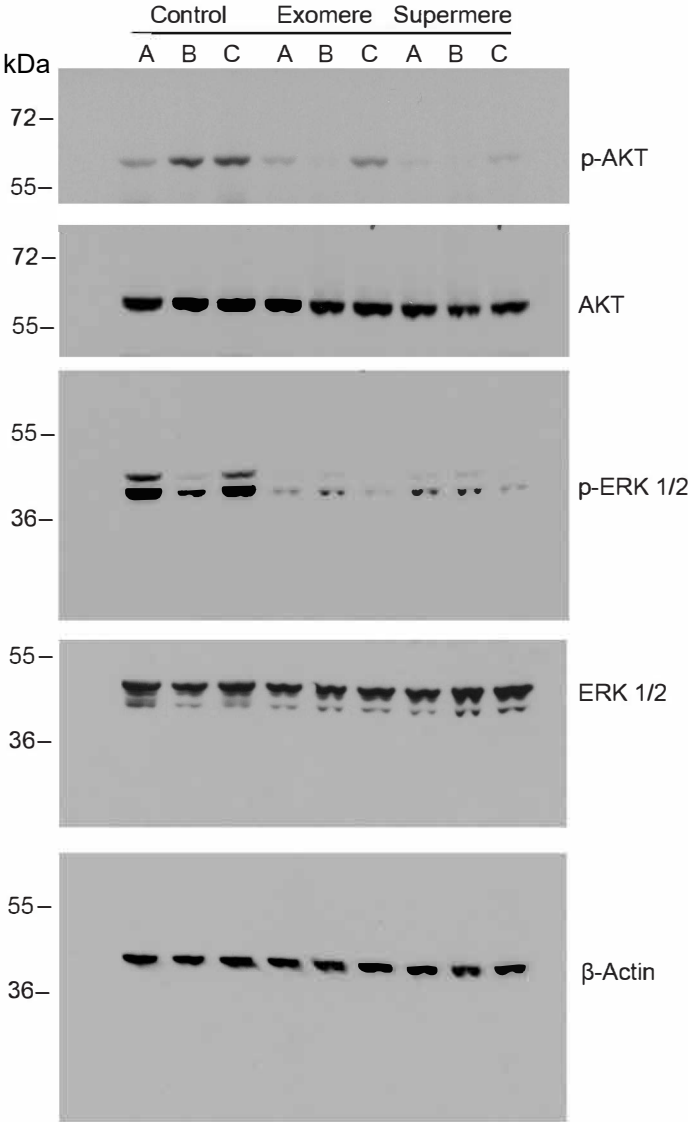

Supplement: Source Data Fig. 6 — Unprocessed western blots. [file 41556_2021_805_MOESM13_ESM.pdf]

**Fig. 7a**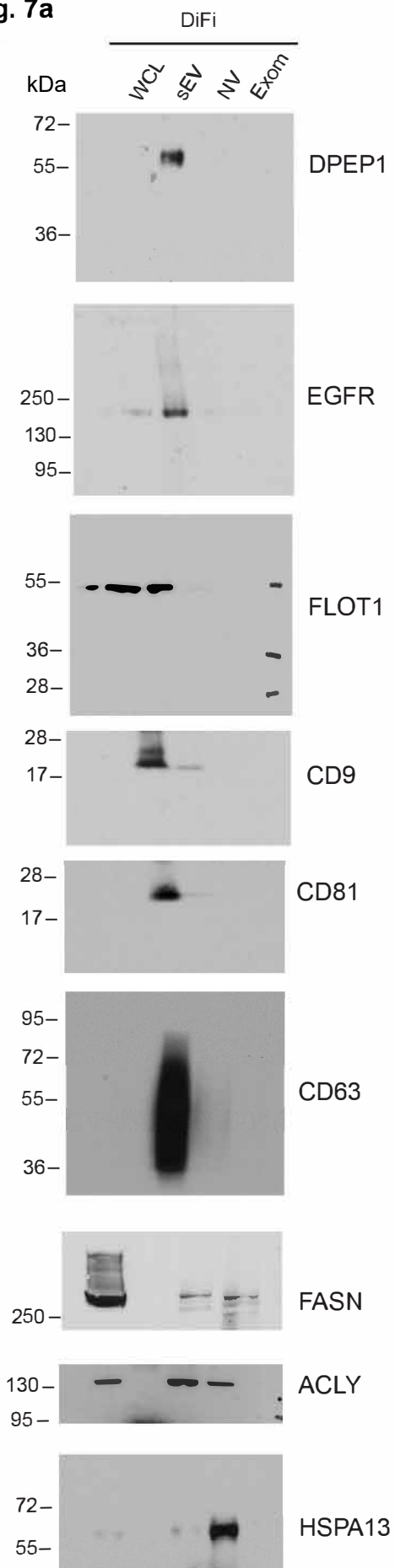**Fig. 7b**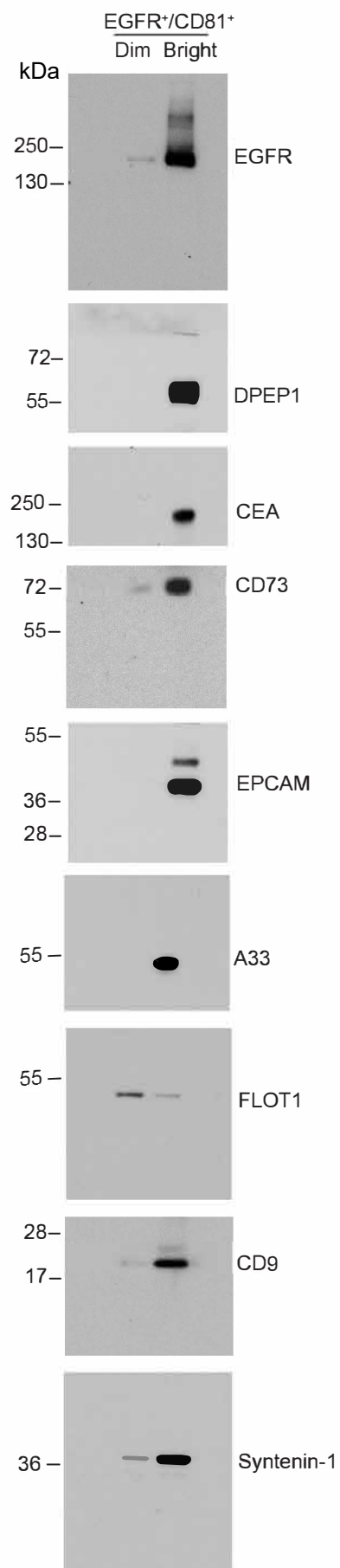

**Fig. 7d**

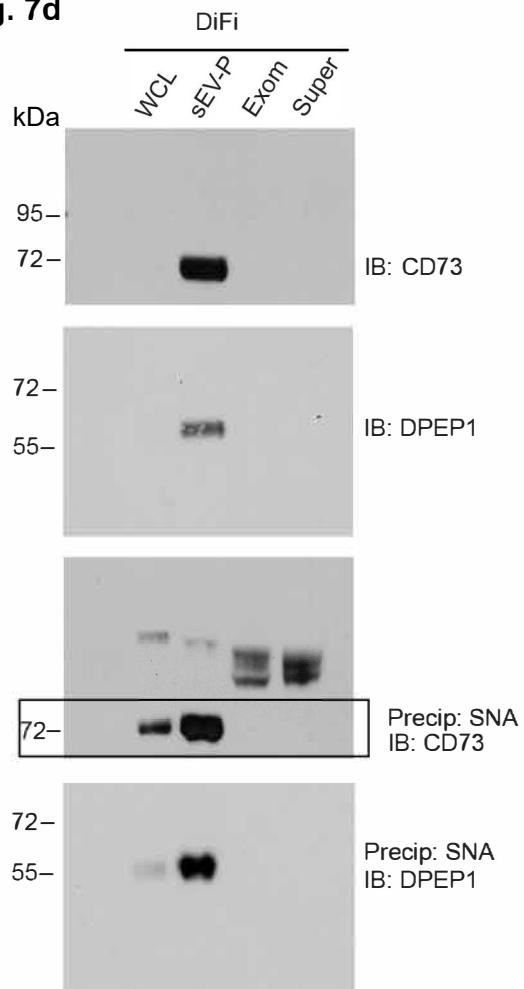

**Fig. 7h**

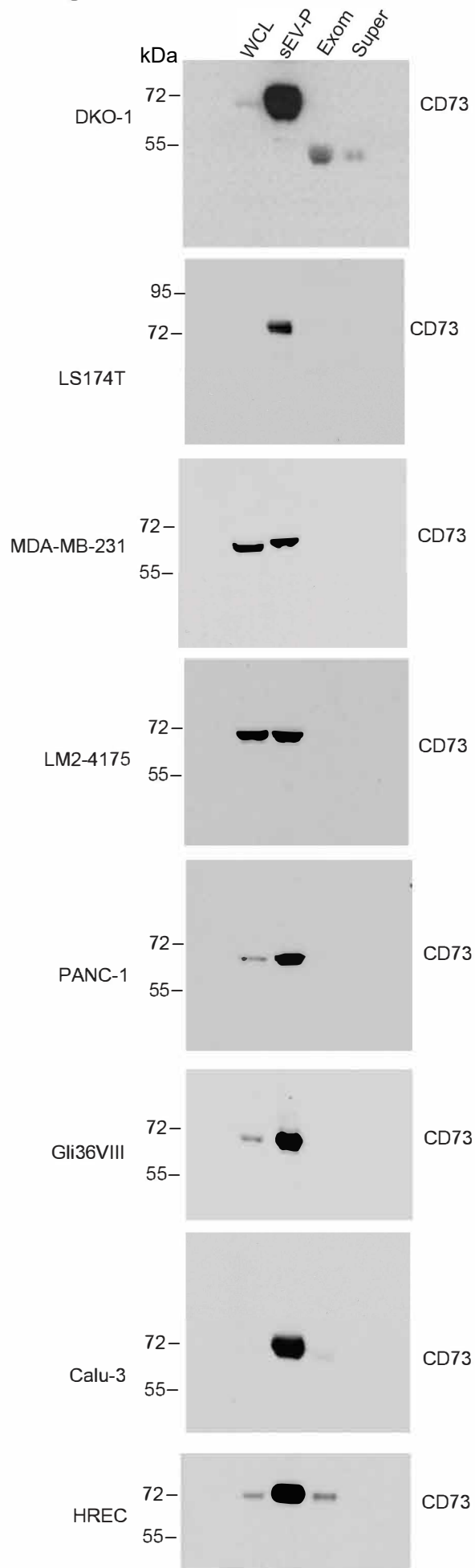

**Fig. 7j**

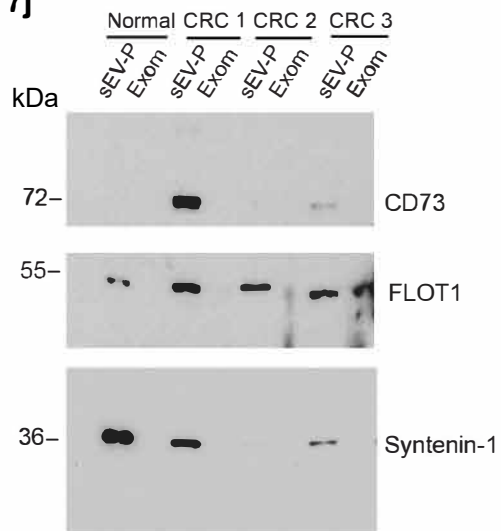

Supplement: Source Data Fig. 7 — Unprocessed western blots. [file 41556_2021_805_MOESM14_ESM.pdf]

Extended Data Fig. 1g

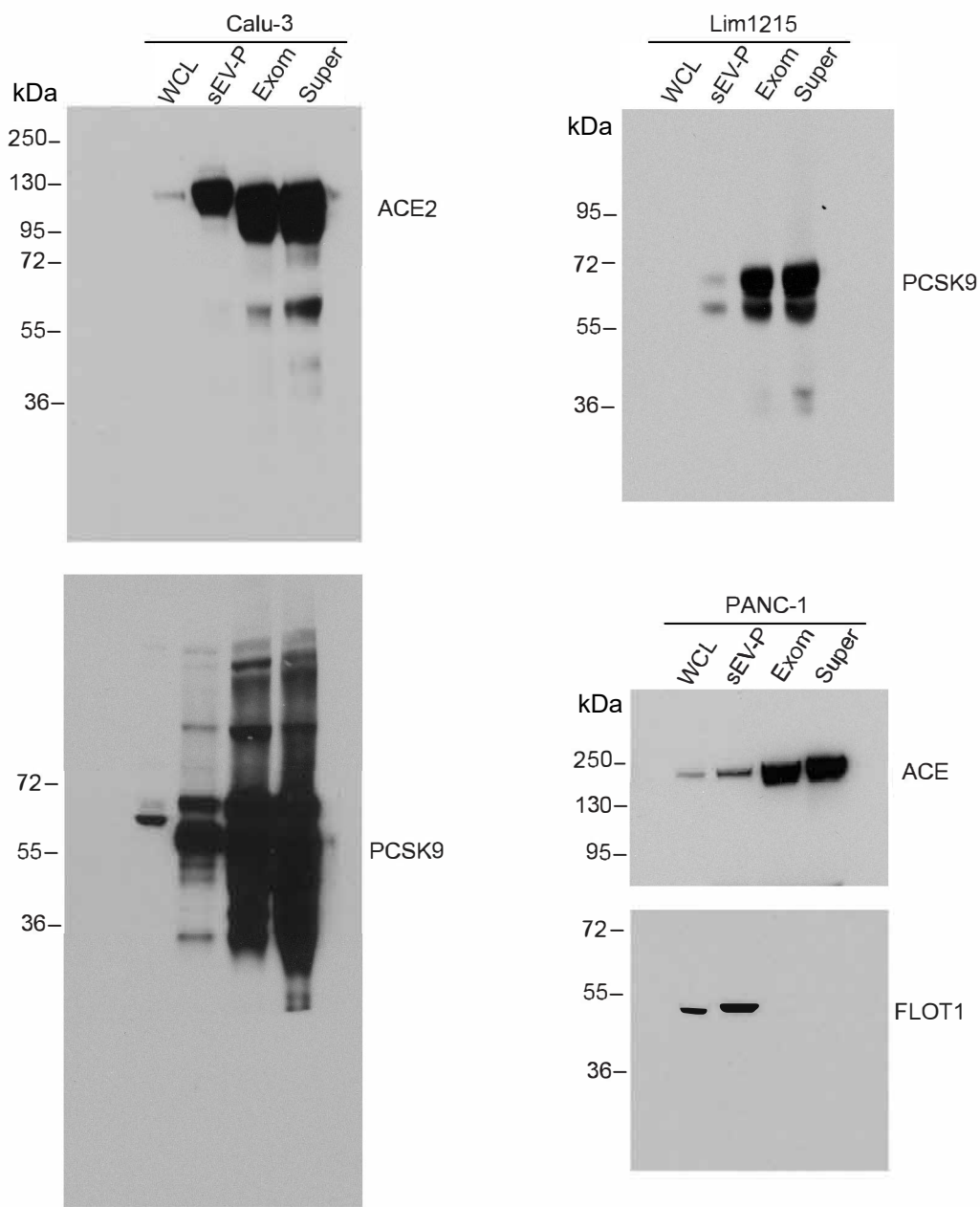

Supplement: Source Data Extended Data Fig. 1 — Unprocessed western blots. [file 41556_2021_805_MOESM15_ESM.pdf]

Extended data Fig. 2d

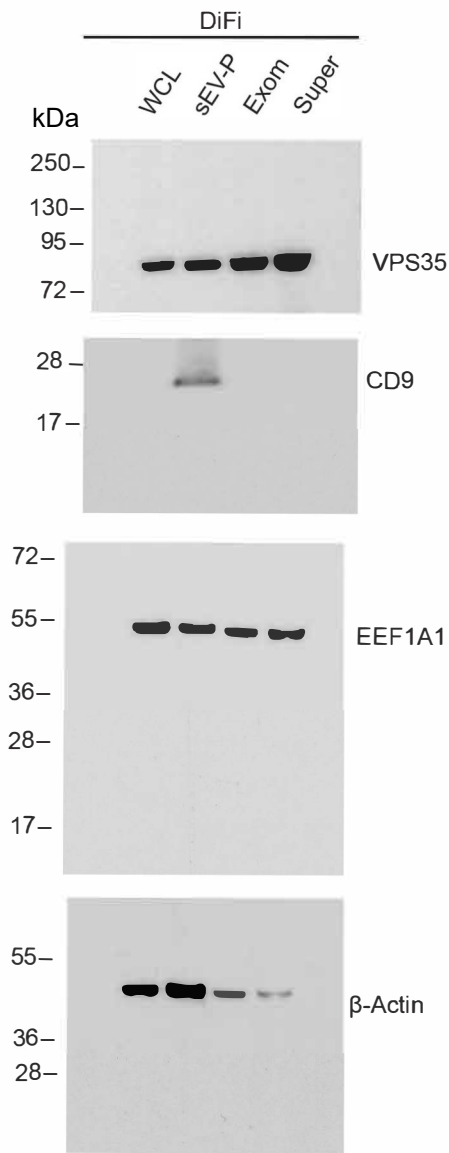

Extended data Fig. 2f

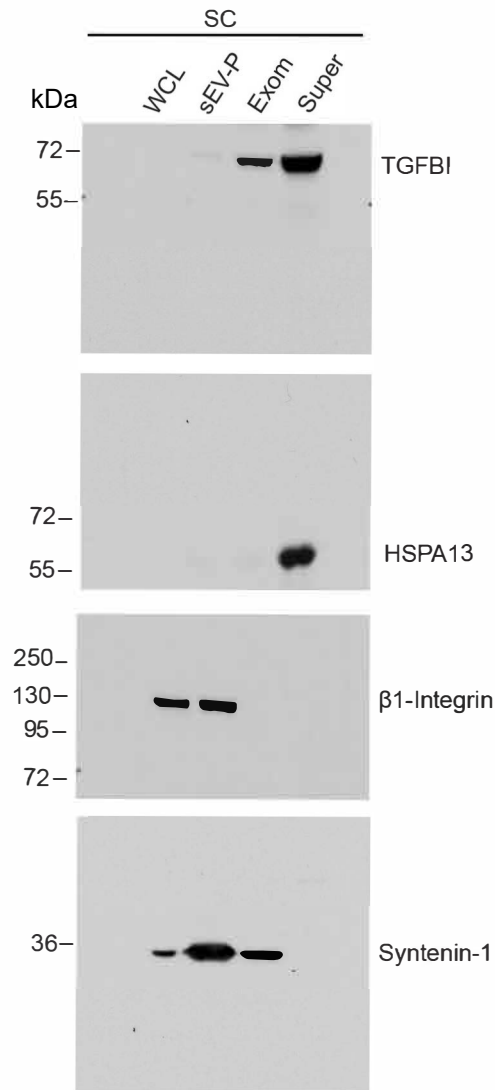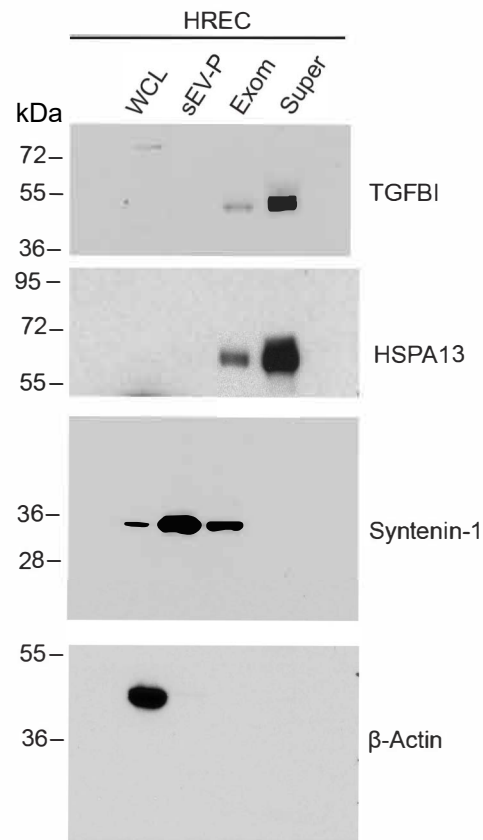

Supplement: Source Data Extended Data Fig. 2 — Unprocessed western blots. [file 41556_2021_805_MOESM17_ESM.pdf]

Extended Data Fig. 5k

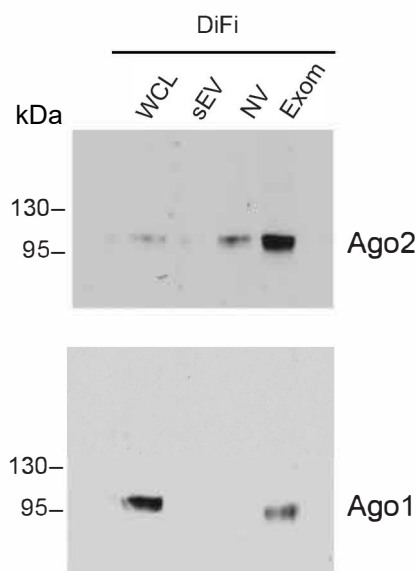

Extended Data Fig. 5m

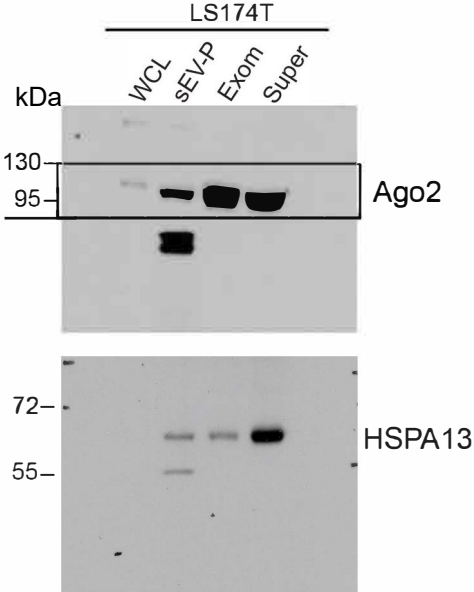

Supplement: Source Data Extended Data Fig. 5 — Unprocessed western blots. [file 41556_2021_805_MOESM21_ESM.pdf]

Extended data Fig. 7a

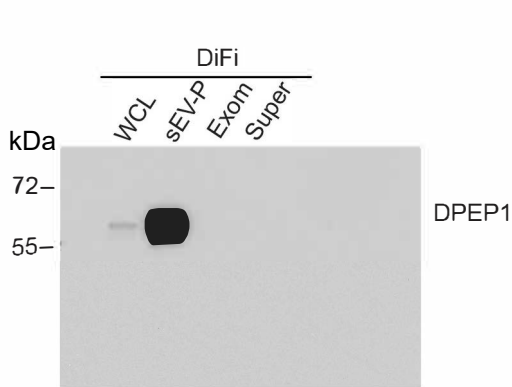

Extended data Fig. 7g

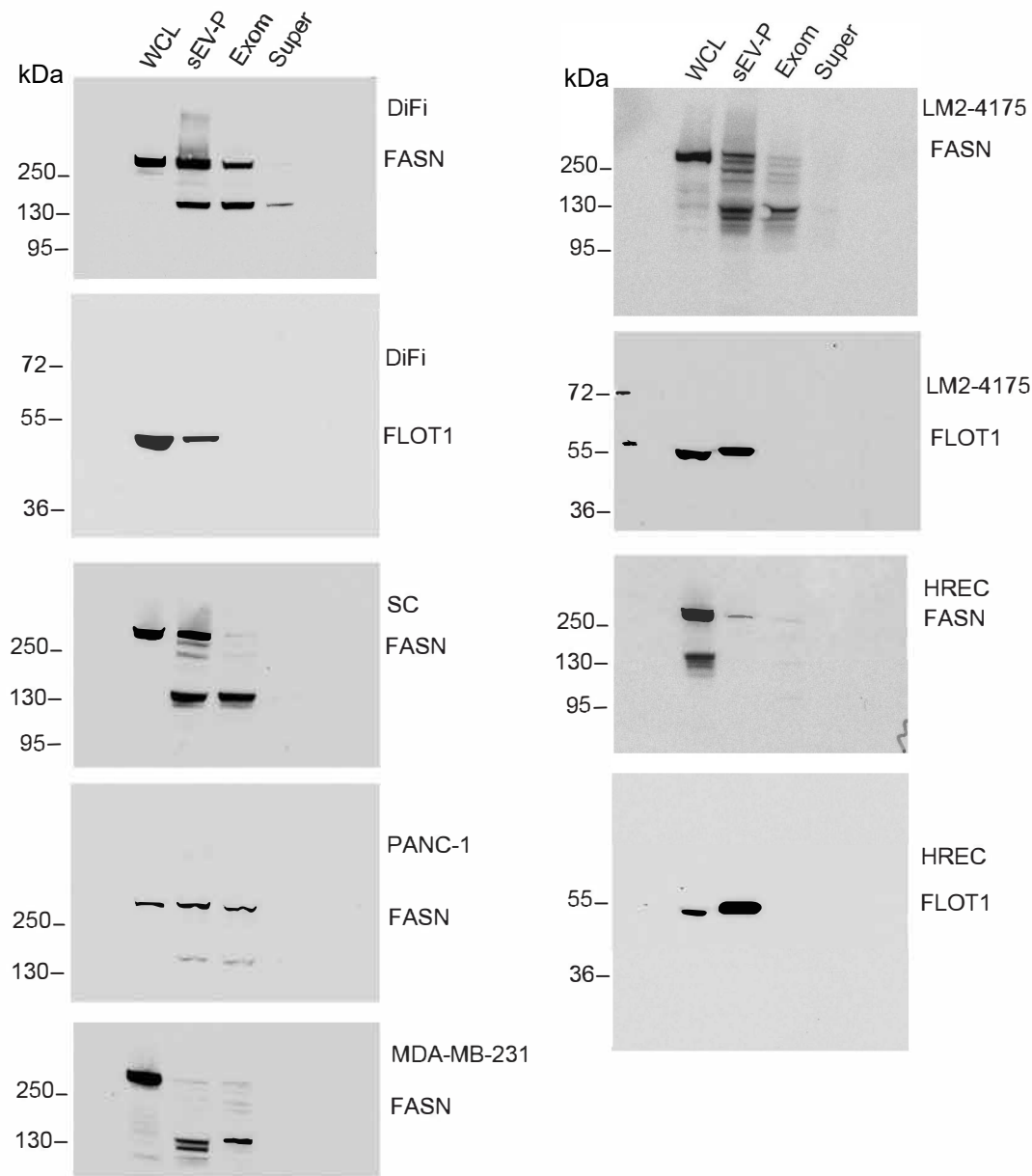

Extended data Fig. 7b

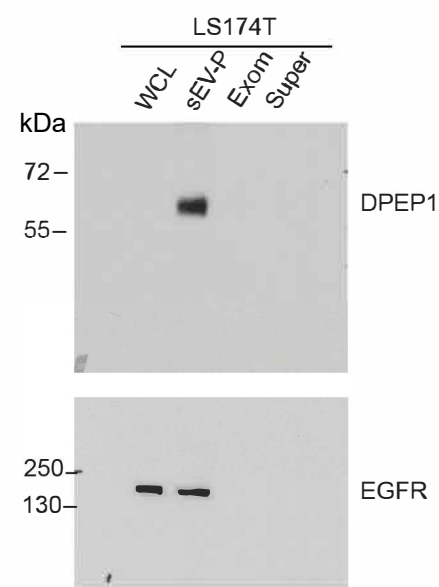

Extended data Fig. 7j

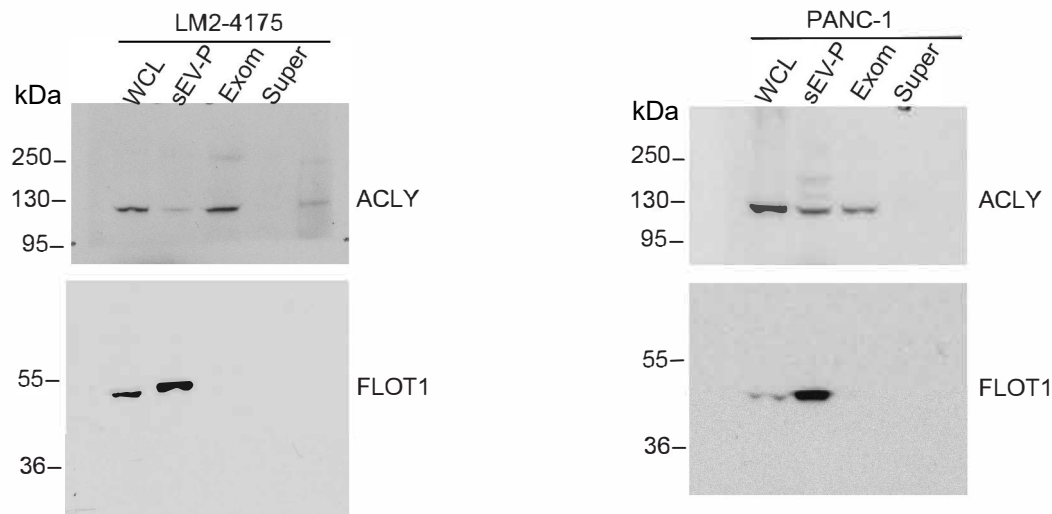

Supplement: Source Data Extended Data Fig. 7 — Unprocessed western blots. [file 41556_2021_805_MOESM23_ESM.pdf]
