# Supplementary material for: Supermeres are functional extracellular nanoparticles replete with disease biomarkers and therapeutic targets
Source: Nat Cell Biol. 2021 Dec 9;23(12):1240–54. doi: 10.1038/s41556-021-00805-8 (PMC8656144; doi:10.1038/s41556-021-00805-8)

Extended Data Fig. 4a

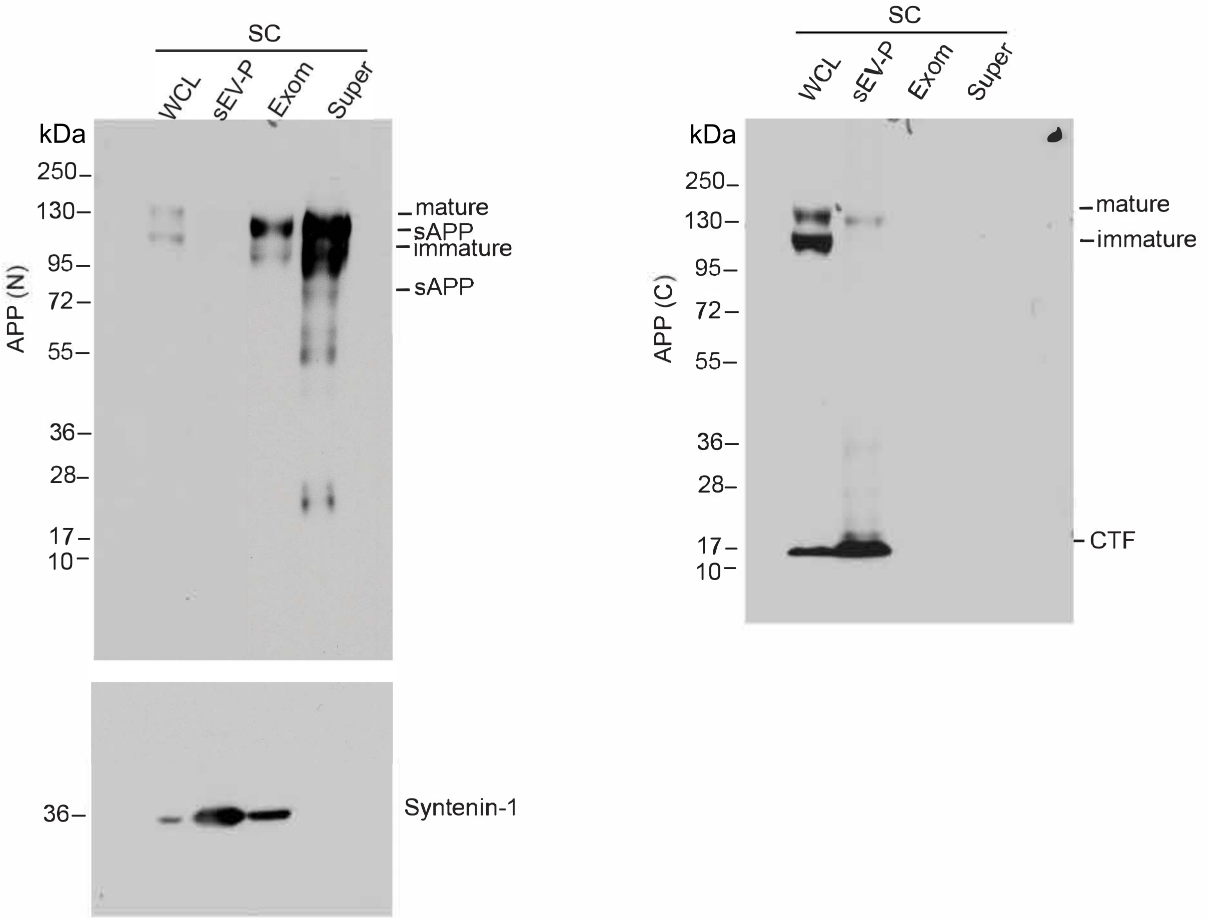

Extended Data Fig. 4b

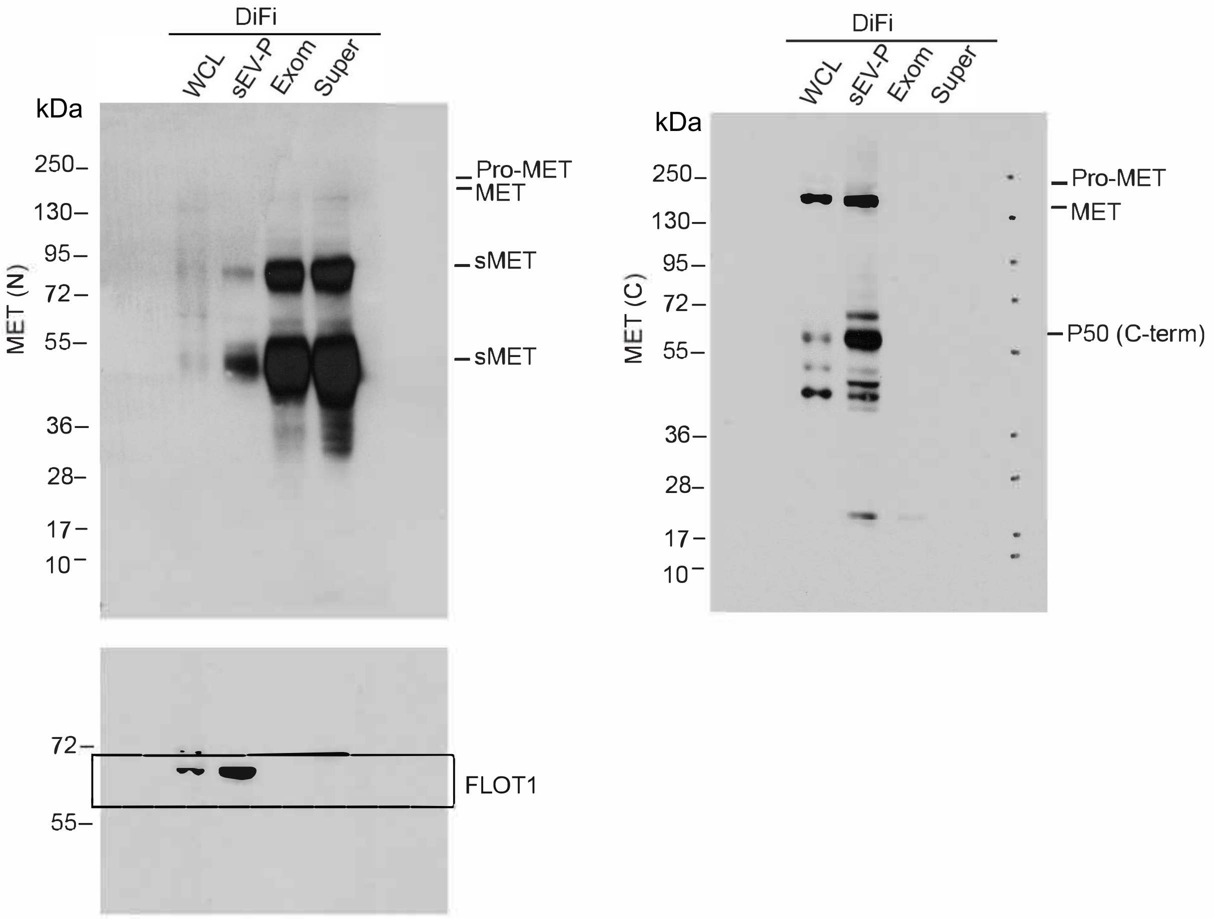

Extended Data Fig. 4c

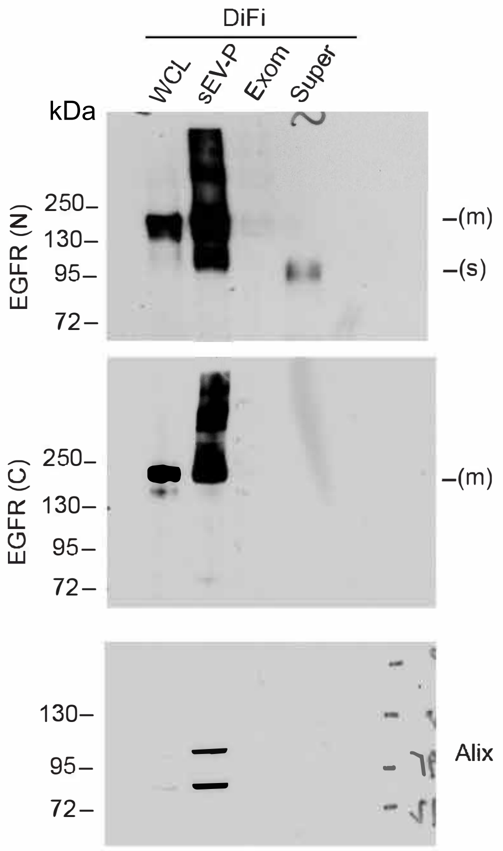

Extended Data Fig. 4e

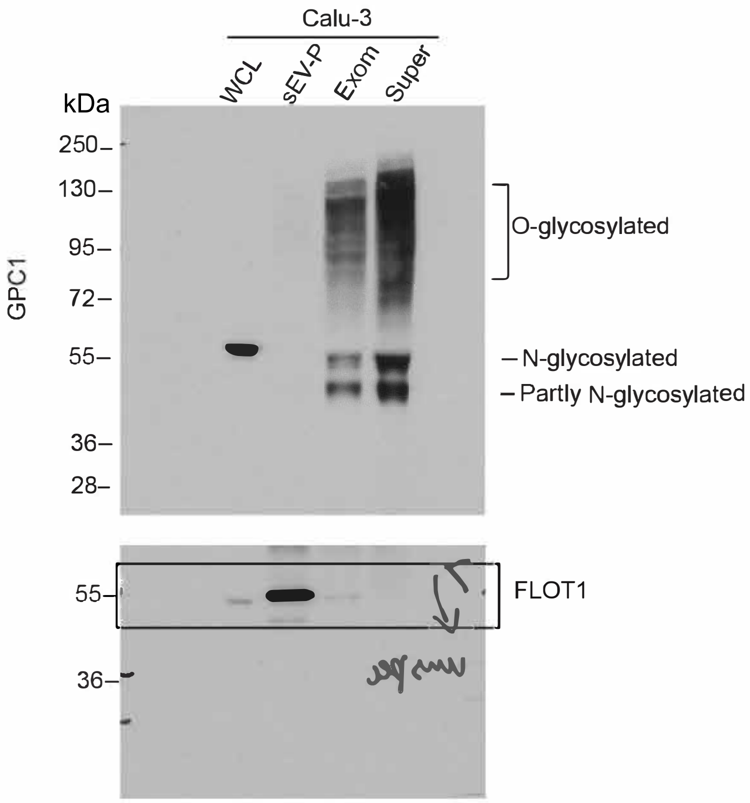

Extended Data Fig. 4d

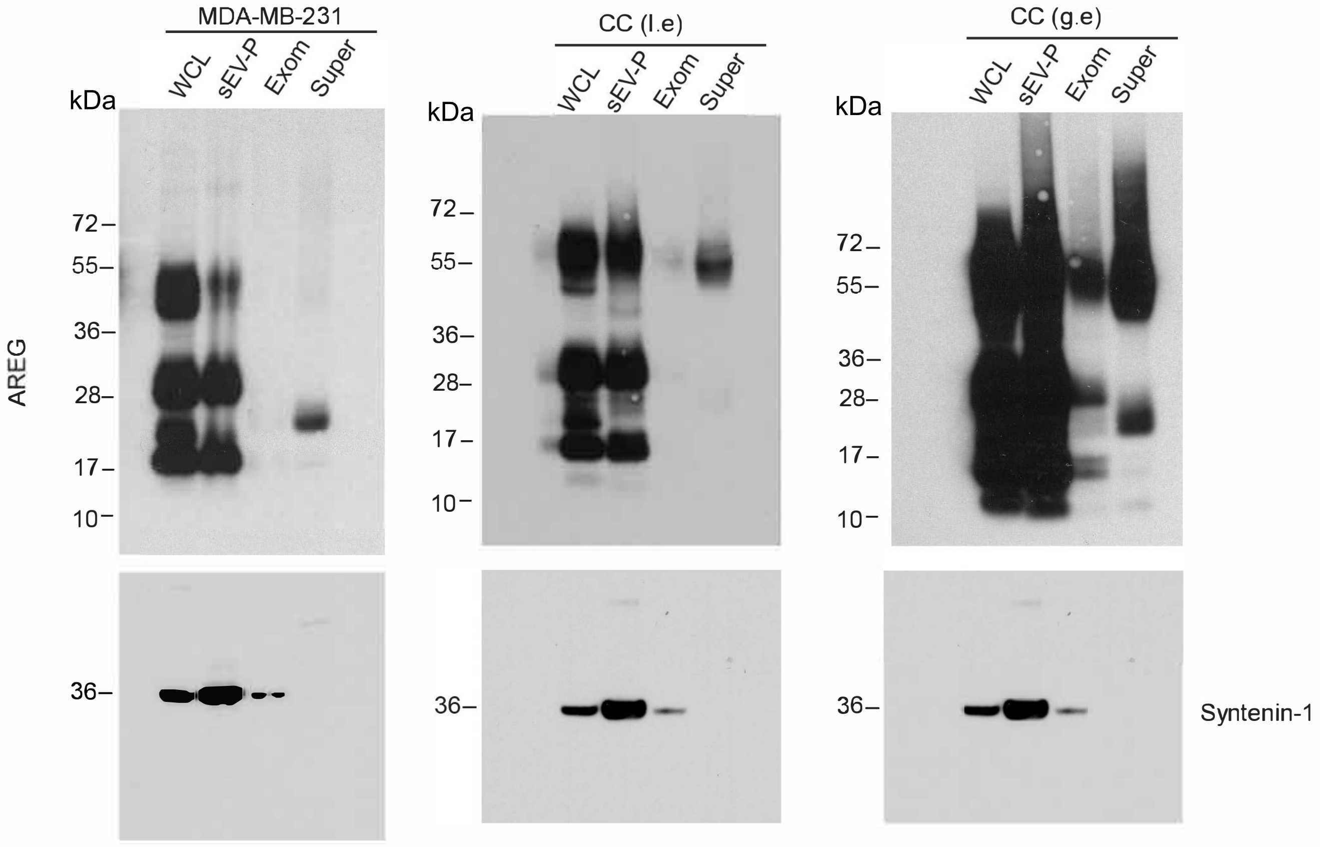

Extended Data Fig. 4f

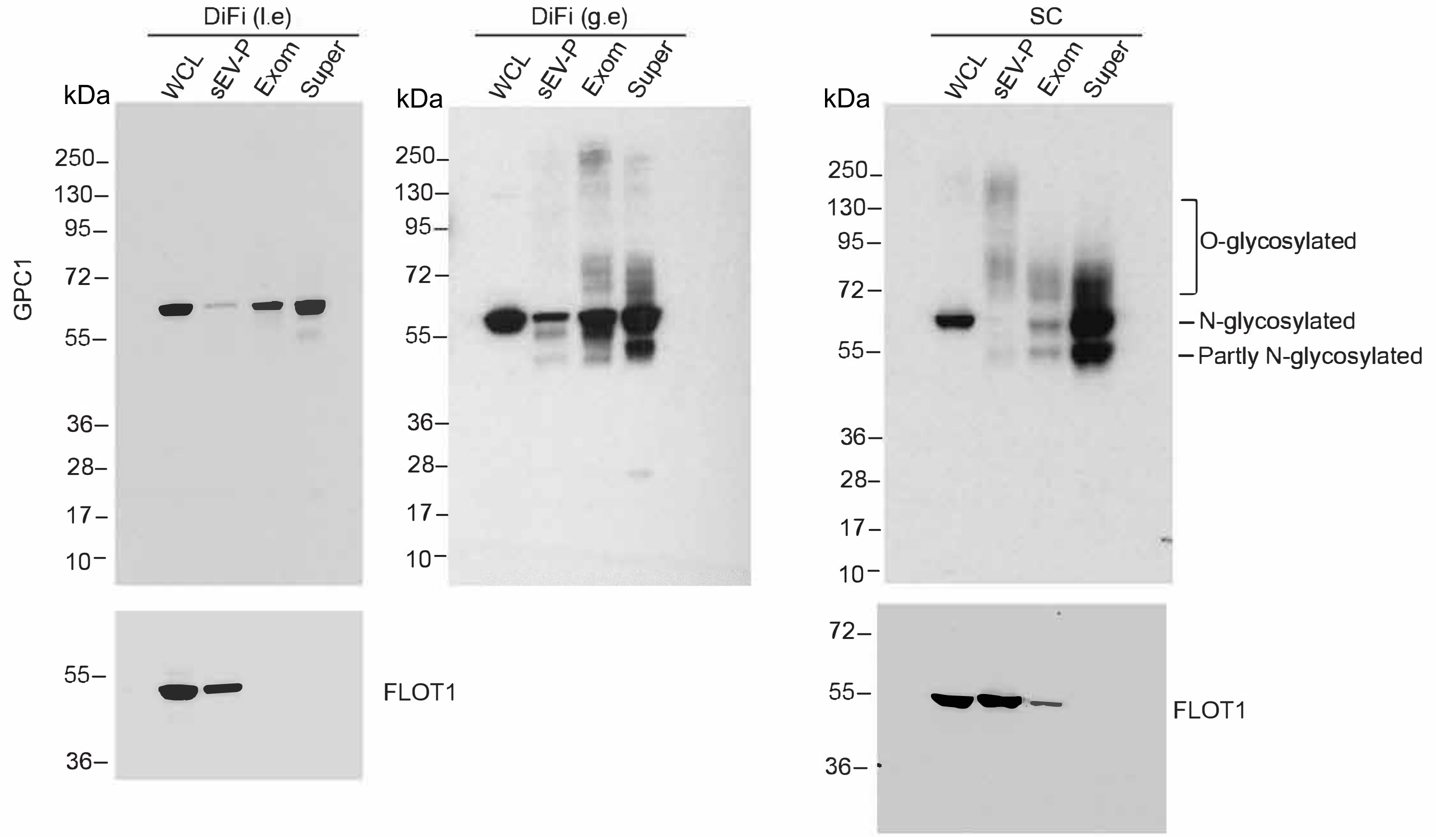

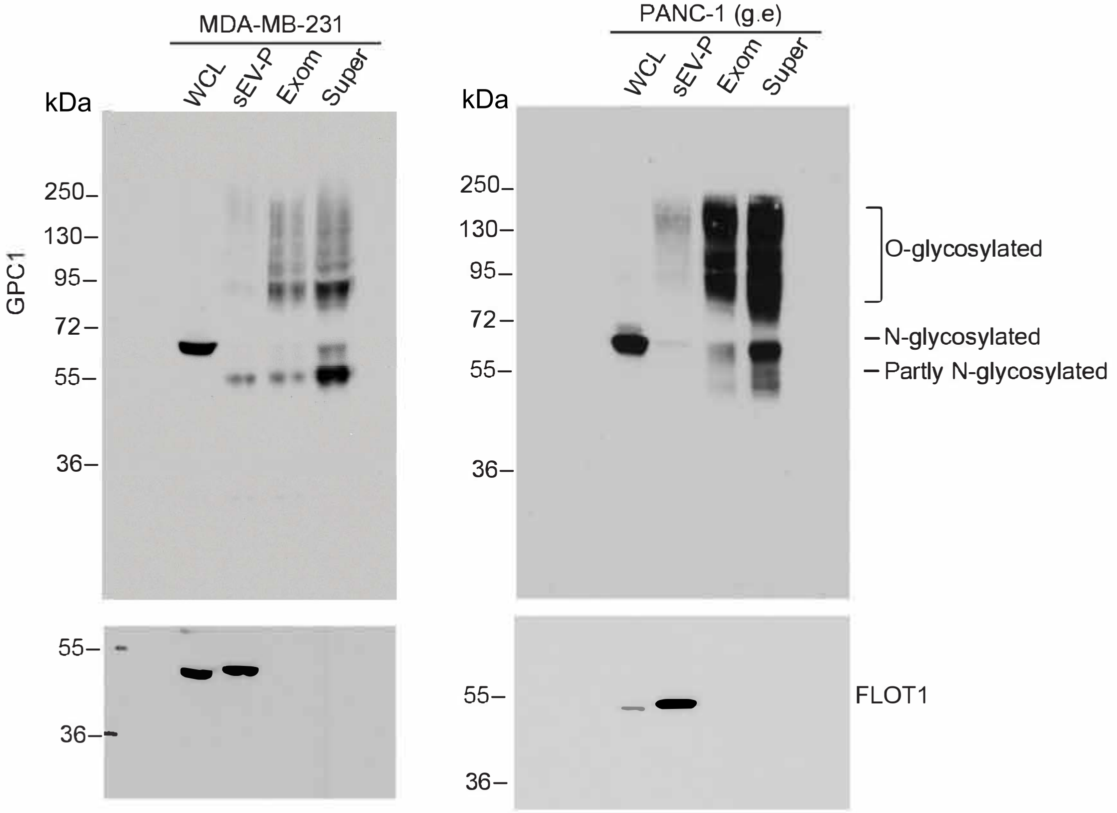

Extended Data Fig. 4g

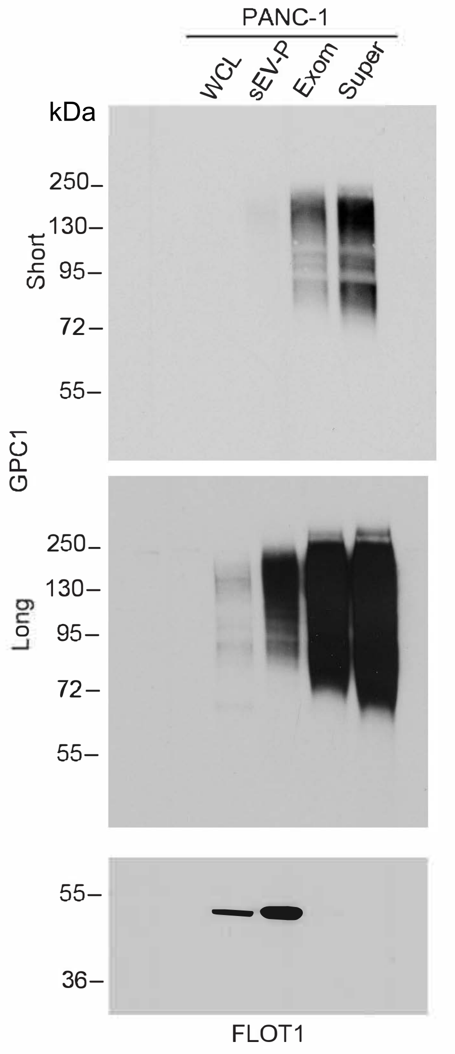

Supplement: Source Data Extended Data Fig. 4 — Unprocessed western blots. [file 41556_2021_805_MOESM19_ESM.pdf]
